# Facile Recovery and Recycling of a Soluble Dirhodium Catalyst in Asymmetric Cyclopropanation via a Catalyst-in-Bag System

**DOI:** 10.1021/acs.oprd.4c00400

**Published:** 2024-10-23

**Authors:** UnJin Ryu, Duc Ly, Kristin Shimabukuro, Huw M. L. Davies, Christopher W. Jones

**Affiliations:** † School of Chemical and Biomolecular Engineering, Georgia Institute of Technology, Atlanta, Georgia 30332, United States; ‡ Department of Chemistry, Emory University, Atlanta, Georgia 30322, United States

**Keywords:** dirhodium tetracarboxylate, homogeneous catalyst, catalyst recycling, catalyst-in-bag, cyclopropanation

## Abstract

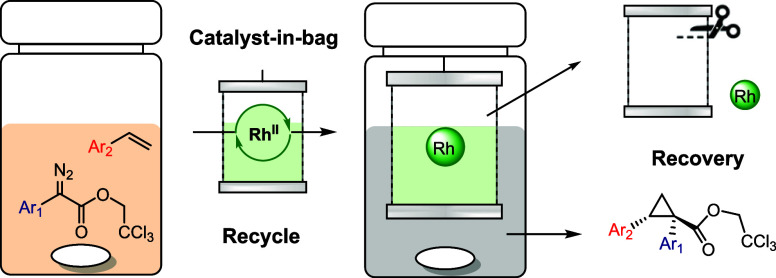

A catalyst-in-bag system facilitates the recovery and recycling of chiral dirhodium carboxylate catalysts used for enantioselective, intermolecular cyclopropanation. The catalyst-in-bag system incorporates a soluble enantioselective dirhodium complex catalyst within a reusable, commercial dialysis membrane. Dirhodium catalysts of different sizes are examined, and two catalysts with molecular weights above 2400 Da are well-retained by the membrane. The catalyst Rh_2_(*S*-TPPTTL)_4_ [TPPTTL = (1,3-dioxo-4,5,6,7-tetraphenylisoindolin-2-yl)-3,3-dimethylbutanoate] is explored in enantioselective cyclopropanation reactions under a variety of conditions. The Rh_2_(*S*-TPPTTL)_4_ catalyst, when contained in the catalyst-in-bag system, provides high yields and enantioselectivities, akin to the homogeneous catalyst in solution, with negligible rhodium permeation out of the bag over five catalytic cycles. The catalyst-in-bag approach facilitates recovery of the expensive rhodium metal and ligand, with only ppm level Rh detected in the reaction products. The flexible and expandable catalyst-in-bag system can be accommodated in vessels of different shapes and dimensions.

## Introduction

Chiral dirhodium carboxylates are highly effective catalysts for synthetically useful transformations of donor/acceptor carbenes.^1,2^ Most notably, they are capable of highly enantioselective cyclopropanations^3,4^ and display exceptional control of regio-, diastereo- and enantioselectivity in C–H functionalization reactions.^2,5,6^ Due to the versatility of the donor/acceptor carbenes, these chiral dirhodium catalysts have been utilized in many synthetic routes for the preparation of pharmaceutically relevant chiral intermediates,^7^ and cyclopropanation chemistry has been applied to three different scale-up campaigns.^7−9^ Although dirhodium carboxylate catalysts are broadly useful, a major concern with their use is the high and fluctuating cost of rhodium, leading to unpredictable production costs. As a result, effective recovery of the catalyst is a desirable and requisite practice for stable production processes.^10^ Furthermore, catalyst recovery has the added benefit of reducing contamination by residual metals in the final product or the waste streams.^10^ One approach for managing the cost of rhodium is to carry out the reactions with very low catalyst loadings.^8,11^ A complementary approach is the recovery of these precious metal catalysts through simple separations enabled by immobilization and/or heterogenization. This approach enables reuse of the rhodium in multiple ways, either by direct catalyst recycling or via high efficiency metal recovery and reprocessing.

Immobilization of chiral dirhodium catalysts has been extensively examined and several different approaches have been explored. The most common strategy is to covalently attach the dirhodium catalysts onto solid supports such as porous silica,^12−15^ monoliths,^16^ cellulose,^17^ hydroxyl resins^18,19^ or poly(styrene).^20^ This approach has been generally effective at recovery of the catalysts with minimal leaching,^12,15^ but does have a drawback as incorporation of the covalent attachments can require significant synthetic effort and the modification can influence the performance of the chiral scaffold.^15^ Another approach is to incorporate the dirhodium catalysts into porous materials such as metal organic frameworks (MOFs),^21−24^ but so far, none of the produced catalysts are capable of high levels of asymmetric induction.^22^ A simpler approach is a designed polymer with coordinating groups capable of binding to the axial sites of the dirhodium catalyst. A highly cross-linked polymer with pyridine linkers was found to be a suitable system, and could immobilize a wide range of chiral dirhodium catalysts without any further catalyst derivatization.^25−27^ Even though the catalyst bound polymer could be recovered and recycled, the system did suffer from significant levels of rhodium leaching compared to the covalently attached catalysts.

**Scheme 1:**
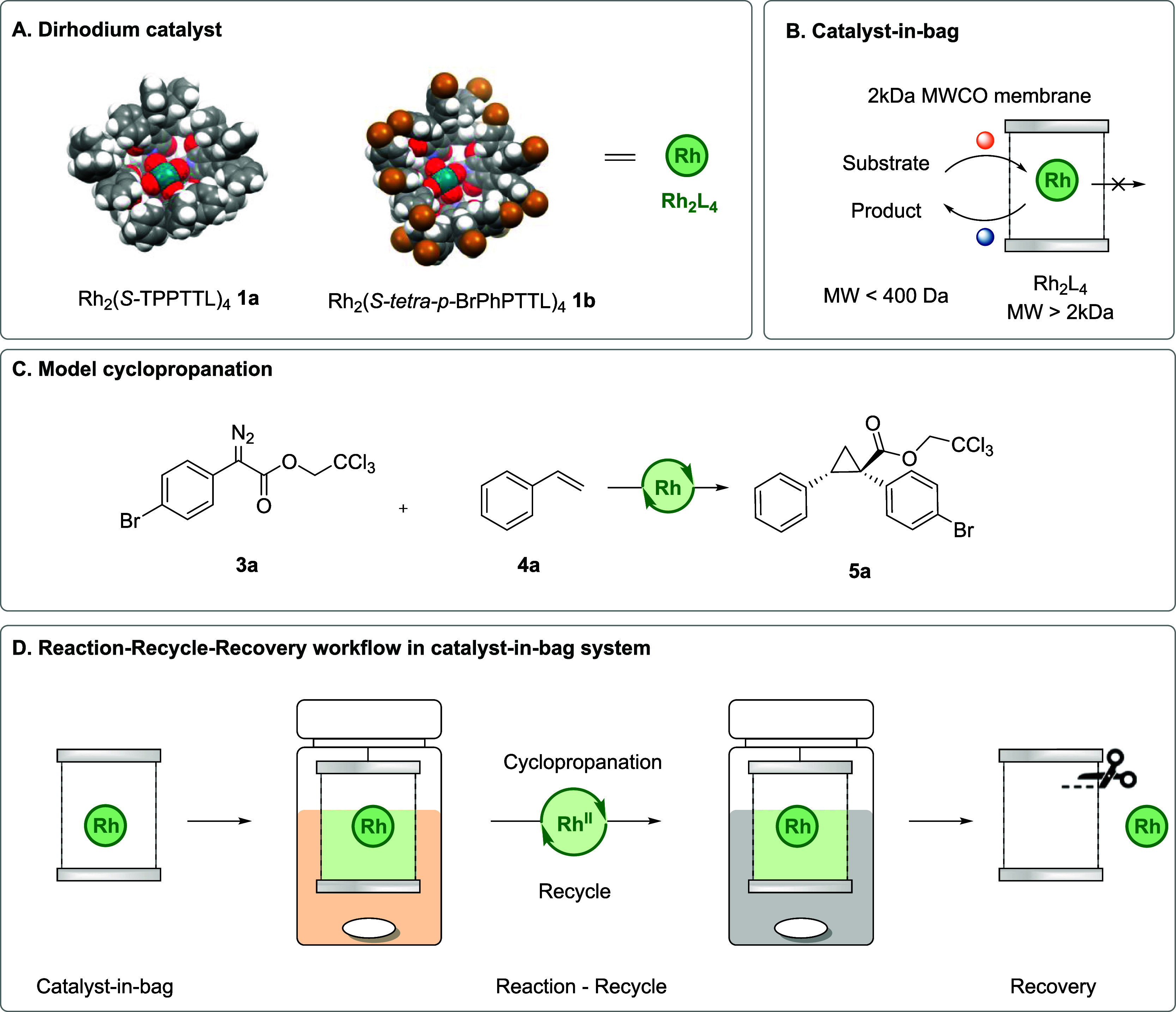
Schematic Approach of Cyclopropanation in the Catalyst-in-Bag System A. 3D X-ray structure of 1a Rh_2_(*S*-TPPTTL)_4_ and 1b Rh_2_(*S*-*tetra-p-*BrPhPTTL)_4_. B. Membrane-assisted reaction in the catalyst-in-bag system by size-dependent permeability. C. Model reaction for the catalyst-in-bag system. D. Illustration of reaction-recycle-recovery workflow in the catalyst-in-bag system

Recently, the Davies group has designed a series of very large chiral dirhodium tetracarboxylate catalysts (MW 2000–5000) to achieve site-selective carbene transformations.^28^ They are readily prepared by the self-assembly of four identical carboxylate ligands and can be further diversified by multifold Suzuki couplings on the preformed catalyst.^29^ Two catalysts that have risen to prominence are the C_4_-symmetric bowl-shaped catalysts, Rh_2_(*S*-TPPTTL)_4_**1a** (MW 2465) and Rh_2_(*S*-*tetra-p-*BrPhPTTL)_4_**1b** (MW 3727) (Scheme 1A). Their molecular weights are approximately 7–10 times larger than the typical reagents (diazo compounds and alkene substrates) used in carbene reactions. We surmised that this significant difference in molecular weight would be sufficient to promote the separation of the catalyst from the reaction mixture using semipermeable membranes (Scheme 1B). Inspired by the simplicity of this separation approach and the potential to avoid the immobilization step that often requires tremendous synthetic effort, we herein demonstrate a “catalyst-in-bag” concept for recovering and recycling homogeneous dirhodium catalysts in asymmetric cyclopropanation reactions (Scheme 1C,D). In this report, we describe our optimization studies to develop suitable membranes and conditions to allow transfer of reagents through the membrane, while retaining the catalysts in the bag, resulting in systems that maintain good yields (75%) and enantiomeric excess (90–94% ee) for up to five cycles of recycling without loss of catalytic performance.

## Results and Discussion

### Preparation and Feasibility of Catalyst-in-Bag System

Scheme 1 describes the overall catalyst-in-bag system for cyclopropanation. This system utilizes a semipermeable membrane shaped like a teabag^30^ that contains the rhodium catalyst in place of tea leaves. This catalyst-in-bag concept was initially introduced for recycling copper catalysts immobilized on dendrimers,^30^ extended to iridium,^31^ palladium,^32^ and ruthenium^33^ catalysts. However, as the reaction progressed, metal leaching was found to increase due to dendrimer instability. In comparison, our catalysts have a comparably stable structure, mitigating metal leaching issues. The catalyst-in-bag design allows free passage of solvents and small substrates through the bag while effectively trapping the larger rhodium catalyst inside the bag (Scheme 1B). This selective permeability is influenced by the polarity of the solvent and the affinity of the membrane for that solvent.^34^ Membrane encapsulation coupled with solubility differences in biphasic systems has been exploited in other transition metal catalyzed reactions, we well.^35^ Given this prior work, we initially performed a feasibility evaluation to identify an appropriate combination of membrane and solvent conditions.

We selected two types of commercially available cellulose-based membranes, regenerated cellulose (RC) and benzoylated cellulose (Bz), both sharing the same cellulose backbone structure but having different molecular weight cutoffs (MWCO) and polarity. The catalyst-in-bag fabrication initially involves the preparation of dry membranes to eliminate the risk of water contamination, which can impact the catalysis. The commercial Bz-membrane, in contrast to the dry RC-membrane, was prewetted in water containing 0.002% 1-hydroxypyridine-2-thione as a preservative. We performed a solvent exchange with methanol and hexane to remove water and remaining polar species, effectively drying the membrane while minimizing shrinkage and preserving its structural integrity.^36^ The stability of the benzoylate groups was probed via FT-IR spectra after the water and hexane treatments (Figure S1). Then, one end of the RC- or Bz-membrane tube was sealed using chemically stable Teflon tape, and catalyst powder was introduced into the bag through a glass pipet. Subsequently, the inside of the bag was purged with Ar gas to minimize air and moisture that may cause side reactions, and the opposite end of the tube was sealed with Teflon tape (Figure S2). Following preparation, we sequentially evaluated the feasibility of the catalyst-in-bag system by examining the permeability of membranes with different solvents (MW 85–88), substrates (MW 104–372), and dirhodium catalysts (MW 779–3727) in ascending order of molecular weight.

Solvent permeability was analyzed by immersing catalyst-in-bags containing Rh_2_(*S*-TPPTTL)_4_ (**1a**) in ethyl acetate (EtOAc, MW 88) or dichloromethane (CH_2_Cl_2_, MW 85), respectively (Figure S3a), as these solvents are representative reaction solvents in cyclopropanation.^8^ As shown in Figure S3b, the EtOAc and CH_2_Cl_2_ solvents diffused into both RC- and Bz-membrane bags and wetted the Rh_2_(*S*-TPPTLL)_4_ catalyst powder after 24 h. The RC-membrane bags, even with higher MWCO (3 kDa and 8 kDa MWCO), did not inflate, whereas the Bz membrane bags (2 kDa MWCO) swelled significantly.

Next, the permeability of 2,2,2-trichloroethyl 2-(4-bromophenyl)-2-diazoacetate (**3a**, MW 372) was analyzed by observing changes in the outer solvents in which membranes containing diazo **3a** were soaked for 1 day (Figure S3c). In EtOAc, 0.3% and 11% of diazoacetate **3a** diffused from through the 3 kDa and 8 kDa MWCO RC membranes, respectively, whereas no diffusion was shown in the CH_2_Cl_2_ solvent. In contrast, the Bz-membrane bag allowed 66% and 63% diffusion of diazo **3a** into both EtOAc and CH_2_Cl_2_ outer solvent, demonstrating its higher compatibility with cyclopropanation conditions (Table S1).

Finally, to assess the permeability of the Rh_2_(*S*-TPPTTL)_4_ catalyst with the Bz-membrane, we examined the rhodium leaching in EtOAc solvent. We comparatively evaluated three different sizes of dirhodium catalysts, Rh_2_(*S*-TPPTTL)_4_**1a**, Rh_2_(*S*-*tetra-p-*BrPhPTTL)_4_**1b** and Rh_2_(Oct)_4_**2,** with molecular weights of 2465, 3727, and 779 Da, respectively (Table 1). The Bz-membrane was not able to retain the smallest catalyst, Rh_2_(Oct)_4_, as 8940 ppm of rhodium was detected in the green outer solvent by ICP-MS (inductively coupled plasma mass spectrometry) (Table 1). Although the observed permeation of Rh_2_(Oct)_4_ through the 2 kDa membrane was only ∼1% of the rhodium added to the bag, presumably due to the low solubility of this catalyst in EtOAc, this rhodium would still be too significant to justify use of the membrane. By comparison, the permeation of Rh_2_(*S*-TPPTTL)_4_ was limited to only 1.3 ppm in EtOAc and 3 ppm in CH_2_Cl_2_. Rh_2_(*S*-*tetra-p-*BrPhPTTL)_4_ showed no rhodium permeation within experimental error. These results demonstrate that the Bz-membrane with 2 kDa MWCO is effective in retaining the larger sized dirhodium catalysts Rh_2_(*S*-TPPTTL)_4_ and Rh_2_(*S*-*tetra-p-*BrPhPTTL)_4_.

**Table 1:**
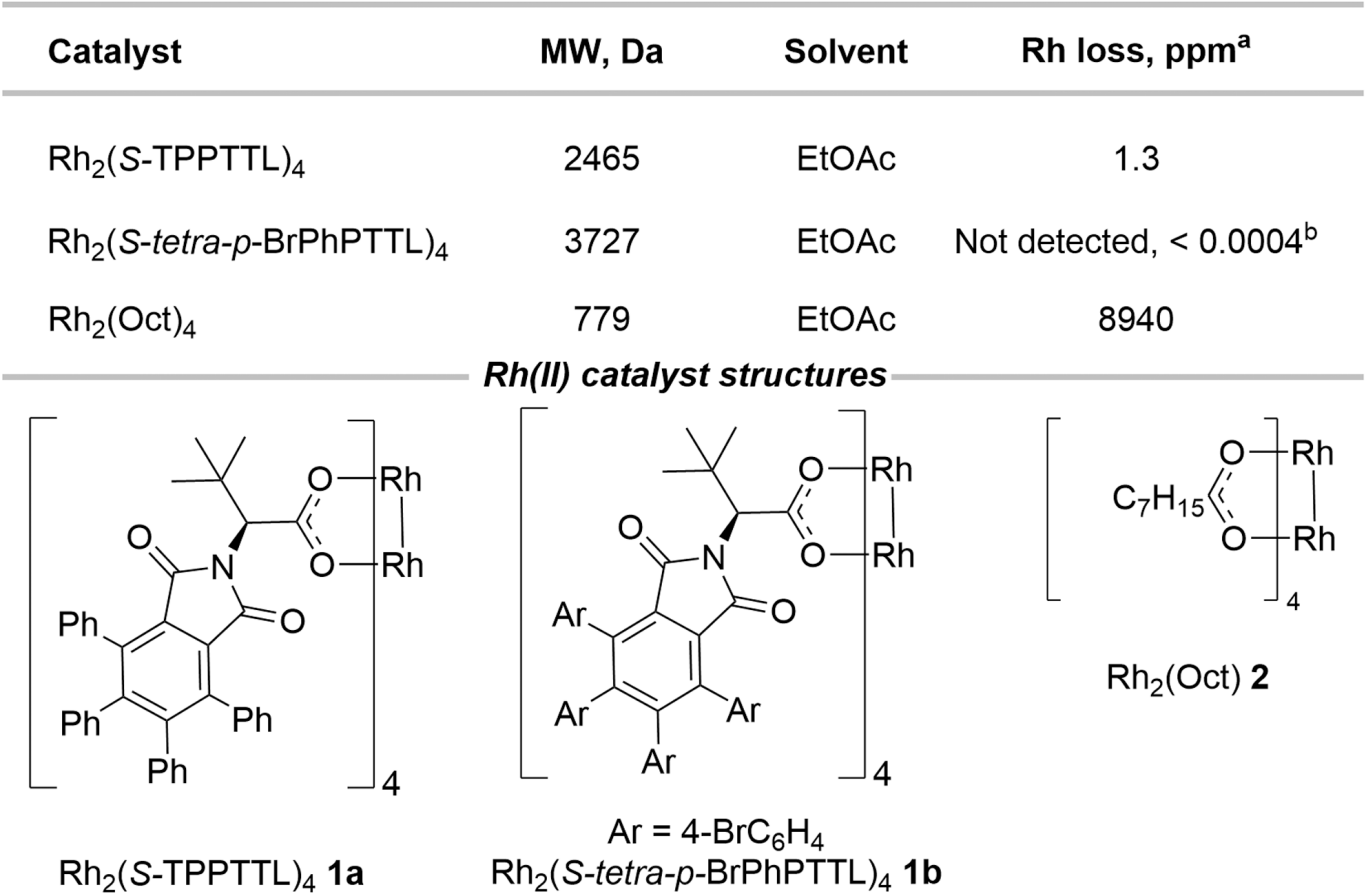
Loss of Dirhodium Catalysts with Different Sizes from the Catalyst-in-Bag^a^^b^

a Permeation levels were quantified using ICP-MS and compared to the original quantity of dirhodium catalysts initially introduced into the bag.

b The value is below the detection limit of the equipment. This value represents the measurement obtained directly from the ICP-MS, rather than indicating a Rh loss.

### Cyclopropanation with Catalyst-in-Bag System

#### Suitability of Catalyst-in-Bag System

To evaluate the efficiency of the catalyst-in-bag system for cyclopropanation, model reactions were conducted with 2,2,2-trichloroethyl 2-(4-bromophenyl)-2-diazoacetate **3a** and styrene **4a** using Rh_2_(*S*-TPPTTL)_4_**1a** at room temperature (25 °C). Before the catalytic reaction, the dry catalyst-in-bag was soaked for 1 day to wet the Bz-membrane and dissolve the catalyst. The soaked bags were then transferred to reaction vials containing a solution of diazoacetate **3a** and styrene **4a**, which were subsequently purged with argon and stirred.

A comparison between catalyst-in-bag conditions and homogeneous conditions showed that the Bz-membrane bag maintained its catalytic performance in both EtOAc and CH_2_Cl_2_ without significant changes in yield and enantioselectivity (Table 2). Enantioselectivities of the catalyst-in-bag systems reached 94% ee in EtOAc and 83% ee in CH_2_Cl_2_ (entries 2 and 4). However, yields were slightly reduced because some products remained inside the bag even after washing twice for product recovery. ^1^H NMR studies also showed that cyclopropanation using the catalyst-in-bag design occurred cleanly without side reactions such as OH-insertion or dimerization. The gradual diffusion of diazo acetate into the bag resembles the conventional slow incremental addition of a semibatch reactor, which helps minimize the risk of dimerization. These results indicate that the Bz-membrane was compatible with the reagents and did not affect the catalytic reaction.

**Table 2:**
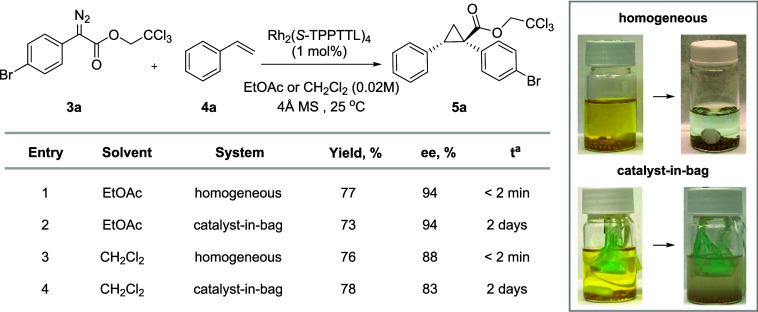
Performance Comparison of Catalyst-in-Bag and Homogeneous Conditions^a^

a Reaction time.

**Figure 1:**
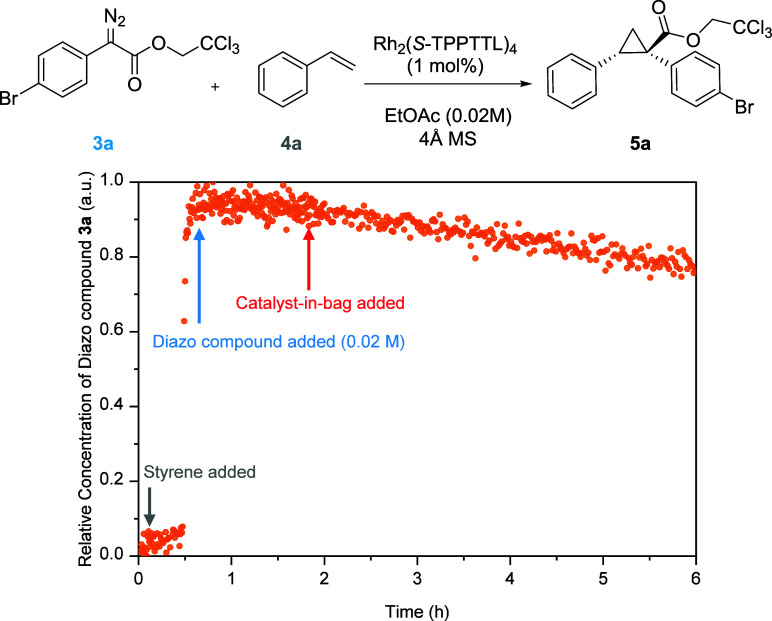
Kinetic investigation to evaluate the suitability of the catalyst-in-bag system using *in situ* FTIR spectrometer, ReactIR.

*In situ* FTIR spectrometery (ReactIR, Mettler Toledo) studies were conducted to monitor the reaction using the catalyst-in-bag system. The reaction progress was analyzed by tracking the disappearance of the distinctive diazo (-N = *N*-) signal at 2096 cm^–1^ from the diazoacetate **3a** (Figure S5).^8^ As shown in Figure 1, styrene **4a** and diazoacetate **3a** were sequentially added to the EtOAc solution at 30 min intervals. As diazoacetate **3a** was added, the intensity of the IR peak increased rapidly and remained unchanged during stirring for 1 h. After the introduction of the catalyst-in-bag into the reaction vessel, the IR signal of diazoacetate began to gradually decrease (Figure 1). This demonstrates that the catalytic reaction was effectively facilitated by diffusion within the catalyst-in-bag system, as intended by our design, as reagents permeated through the membrane to react with the catalyst.

While the catalyst-in-bag design was successfully applied to the cyclopropanation of diazo acetate **3a** with styrene **4a**, the reaction rate was significantly slower than that of homogeneous conditions, with the complete conversion taking up to 2 days instead of minutes (Table 2),^8^ presumably due to diffusion limitations of the Bz-membrane. An *in situ* IR spectrometer was utilized to gain a better understanding of the kinetic behavior of the catalyst-in-bag system. To establish optimal reaction conditions for a kinetic perspective, we investigated the effects of temperature, catalyst concentration, and substrate concentration on the reaction rate.

The effect of temperature on the cyclopropanation within the catalyst-in-bag system was analyzed at 25 °C, 40 °C, and 60 °C. The reaction showed a first order relationship with diazoacetate **3a**. As the temperature increased, both the reaction time and half-life (*t*_1/2_) decreased from 47 h (*t*_1/2_ of 12.5 h) at 25 °C to 16 h (*t*_1/2_ of 3 h) at 40 °C, and further to 9 h (*t*_1/2_ of 1.5 h) at 60 °C. Consequently, the reaction rate coefficients (k) increased with temperature, as shown in Figure S6. Rhodium leaching was observed to remain constant at lower temperatures, measuring 1.9 ppm at 25 °C and 2.2 ppm at 40 °C, but increased slightly at 60 °C to 19 ppm. The yields of cyclopropane **5a** slightly increased with increasing temperatures to 77%, 82%, and 81%, respectively, while the enantioselectivity (ee%) decreased to 94%, 92%, and 87%, respectively. These results indicate that temperature not only improves membrane diffusion but also impacts catalyst performance. Considering that the catalyst-in-bag system showed reasonable reaction rates at 40 °C for ReactIR studies, we pursued further kinetic studies at this temperature.

**Figure 2:**
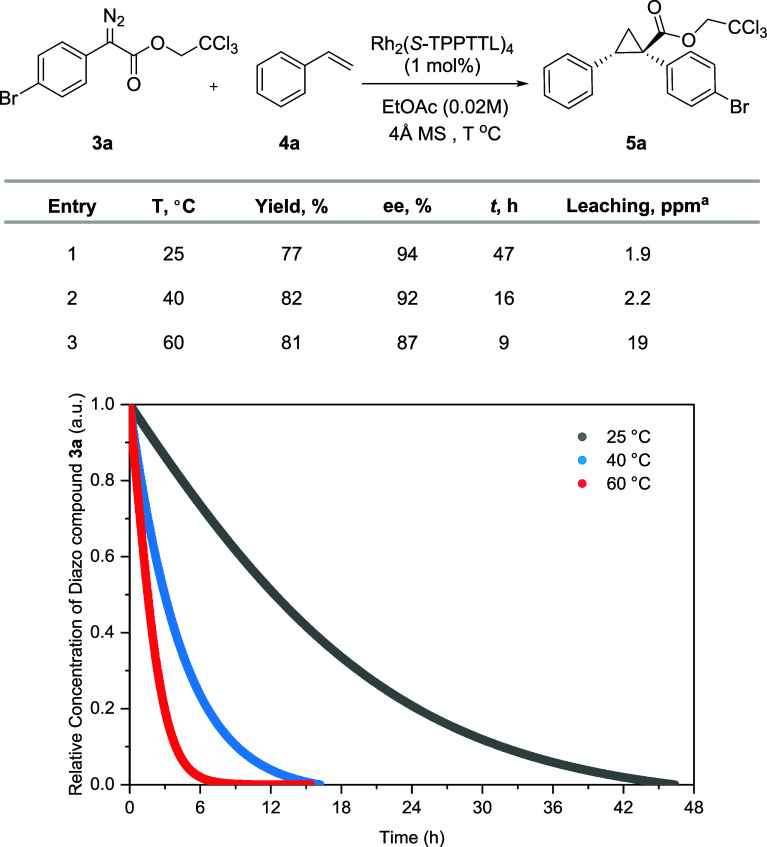
Kinetic profiles of cyclopropanation using the catalyst-in-bag system at different temperatures (25 °C, 40 °C and 60 °C). ^a^Permeation levels were quantified using ICP-MS and compared to the original quantity of dirhodium catalysts initially introduced into the bag.

**Figure 3:**
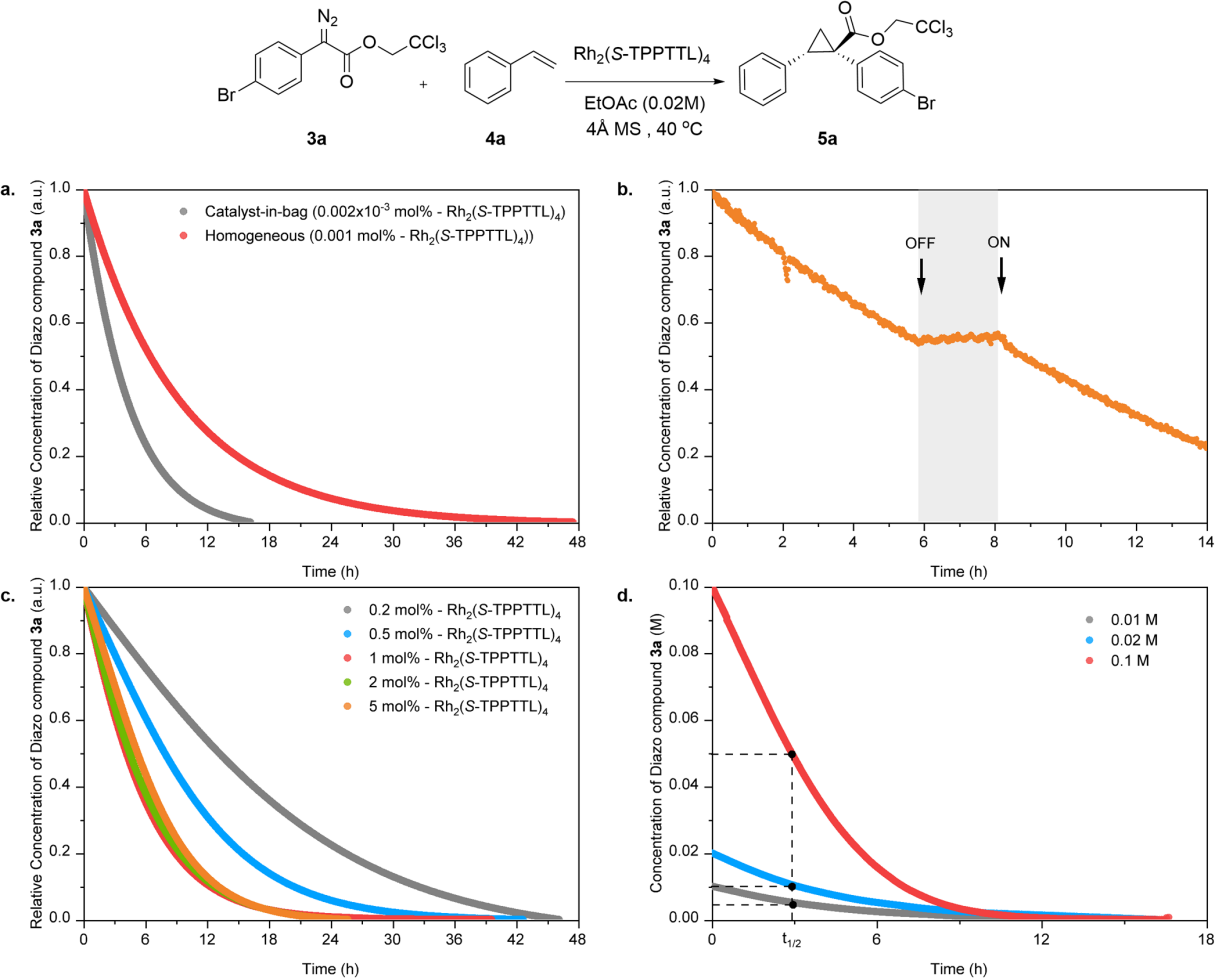
Kinetic profiles for cyclopropanation using the catalyst-in-bag system at 40 °C. (a) Comparative tests to the homogeneous catalyst at low catalyst loading, 500 times higher than the leaching amount from catalyst-in-bag. (b) On/off test based on the presence or absence of a bag. (c) Reaction rate with different catalyst loadings. (d) Reaction rate with different substrate concentration keeping 3a and 4a ratio at 1:5. Unless otherwise noted, all reactions were carried out with 1.0 mol % Rh_2_(*S*-TPPTTL)_4_.

Control experiments were conducted to evaluate the reliability of the kinetic analysis in the catalyst-in-bag system. Based on the detection of 2.2 ppm of rhodium (1.1 × 10^–5^ mg of Rh_2_(*S*-TPPTTL)_4_, 0.002 × 10^–3^ mol % catalyst loading) in the external solvent after reaction at 40 °C (Figure 2), we evaluated whether the observed catalytic activity could be due solely to rhodium that permeated through the membrane. In comparative tests shown in Figure 3a, a homogeneous catalytic reaction using 0.001 mol % catalyst loading (0.5 × 10^–2^ mg of Rh_2_(*S*-TPPTTL)_4_) required more than 42 h to complete the reaction, significantly longer than the catalyst-in-bag system, suggesting that the minimal rhodium permeation could not be the sole source of the observed reactivity with the catalyst-in-bag system and has an overall negligible impact on the observed rate. Additionally, an on/off experiment confirmed the direct influence of the catalyst-in-bag on the reaction progress; removal of the bag halted the reaction, which resumed upon reintroduction (Figure 3b). This demonstrates that the kinetic evaluation via *in situ* IR of the catalyst-in-bag system is not only reliable but also maintains the integrity of the system.

The catalyst loading was varied at 0.2, 0.5, 1, 2, and 5 mol % with constant concentrations of styrene and diazoacetate (Figure 3c). The reaction rate remained consistent at catalyst loadings between 1 and 5 mol %, but gradually decreased as the catalyst loading was reduced to 0.5 and 0.2 mol %. Although the rate-determining step is diffusion of substrate through the membrane, these results suggest that the activity of the catalyst can influence the diffusion process due to its effect on the concentration gradient. In a previous study, the variable time normalization analysis (VTNA) method demonstrated that the Rh_2_(*S*-TPPTTL)_4_ catalyst has first-order kinetics.^8^ The rate law for the cyclopropanation using rhodium(II) is known as follows:
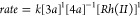
1

The reaction profiles were plotted against a normalized time scale Time[cat]_T_^n^ (([cat]_T_ = Total catalyst concentration, *n* = reaction order of the catalyst to be determined) with *n* = 1, and it showed a similar kinetic profile from 0.2 to 1 mol % catalyst loading, but gradually slowed down at 2 mol % and 5 mol % (Figure S7b). This indicates that diffusion limitations hinder the full utilization of the catalyst at higher concentrations, such as 2 mol % and 5 mol %. The optimal catalyst concentration that balances both diffusion and reaction rates at 0.02 M substrate concentration is 1 mol %.

Cyclopropanation reactions were then conducted using different concentrations of diazoacetate **3a** styrene **4a** and at 0.01 M, 0.02 and 0.1 M, keeping the catalyst loading at 1 mol %. As shown in Figure 3d, the reaction under the higher concentration diazoacetate **3a** and styrene **4a** conditions converted more to cyclopropane in the same amount of time, but the half-life (*t*_1/2_) for all conditions is the same, indicating that the catalyst-in-bag is a diffusion-limited system. To mitigate the impact of concentration differences, we normalized the cyclopropanation kinetics based on [**3a**]. The results showed that conversion rates remained constant across all tested concentrations (Figure S8). This consistency suggests the catalyst-in-bag system is diffusion-dominated and exhibits pseudo-first-order behavior (eq 1). However, after the first half-life (*t*_1/2_), the reaction kinetics at 0.01 and 0.02 M became slower compared to the 0.1 M condition. This appears to be a result of the reduced diffusion rate due to the lower absolute concentrations of **3a** and **4a** in the 0.01 and 0.02 M conditions. Thus, the catalyst-in-bag systems have reaction kinetics in which an additional diffusion element is introduced compared to the homogeneous system. To improve conversion rates to get more product within the same time frame, increasing the mass of substrates can overcome the diffusion limitations, highlighting the importance of substrate concentration in catalyst-in-bag systems.

#### Compatibility of Catalyst-in-Bag System

**Figure 4:**
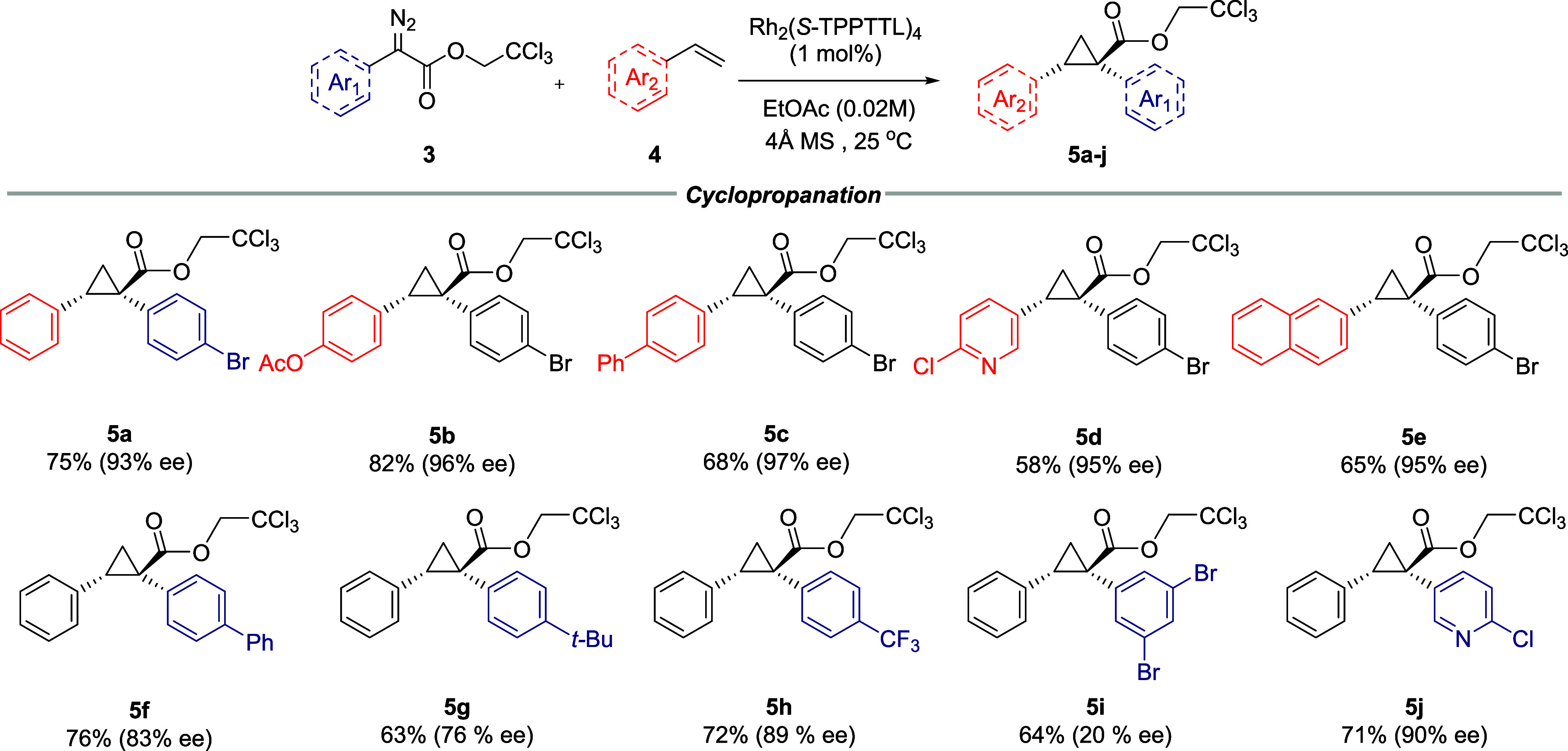
Compatibility of benzylated cellulose membranes with different diazo and alkene compounds. Reaction conditions: **3** (0.2 mmol, 1.0 equiv), **4** (5 equiv), Rh_2_(*S*-TPPTTL)_4_ (1 mol %, in membrane), and 4 Å molecular sieves (100 wt %) in ethyl acetate (10 mL, 0.02 M) at 25 °C for 2 days.

The catalyst-in-bag system utilizing benzoylated cellulose membranes works well using the standard reagents, diazoacetate **3a** and styrene **4a**. To further evaluate the scalability and compatibility of the catalyst-in-bag system, we conducted 9 different cyclopropanation reactions using 5 diazoacetates and 4 alkenes, each with diverse functional groups (Figures 4 and S9). The addition of functional groups to the substrate did not interfere with its ability to pass through the 2 kDa Bz-membrane, and the cyclopropanations of the 9 different substrate combinations were completed successfully. The reaction of product **5a** served as the standard. Moreover, the resulting products (**5b**–**5j**) were produced with good yields and enantioselectivities. These data show that the catalyst-in-bag system can be broadly applied to various cyclopropanation reactions.

#### Recyclability of the Catalyst-in-Bag System

To investigate the recyclability and stability of the catalyst-in-bag system, the bags were reused multiple times in both EtOAc and CH_2_Cl_2_ at 25 °C. The recycling experiments were conducted under identical conditions for each cycle. After completion of each reaction, the catalyst-in-bag was washed twice with fresh solvent before it was introduced into a new batch of reaction solvent. This process was repeated to achieve 5 cycles of reactions (Figure S10).

**Figure 5:**
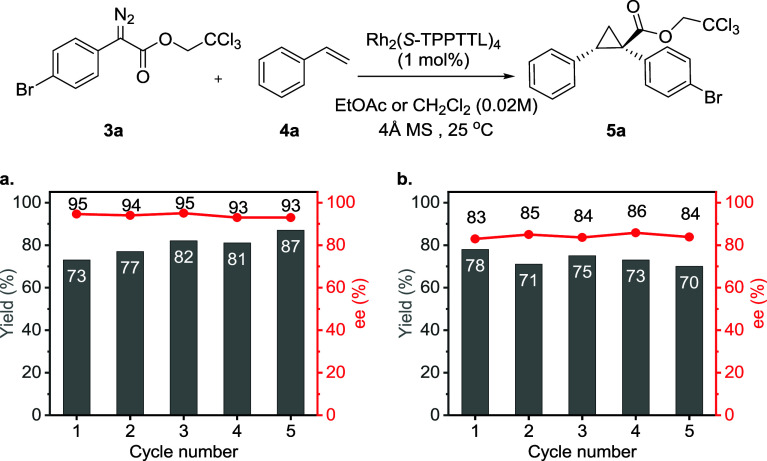
Recycling studies of the catalyst-in-bag system (a) using EtOAc as solvent with average reaction times of 1–2 days, and (b) using CH_2_Cl_2_ as solvent with average reaction times 1–1.5 days.

With both solvents, EtOAc (Figure 5a) or CH_2_Cl_2_ (Figure 5b), the membrane showed reproducible performance and that it could be reused for up to 5 cycles without any significant changes in yields and enantioselectivity compared the first catalyst-in-bag cycle. These values are consistent with the results obtained under homogeneous conditions (Table 2). As the bag was reused, the apparent yield slightly increased (EtOAc) or fluctuated (CH_2_Cl_2_). The increasing yield is because the products were not completely recovered during the washing process in the prior cycle. We also observed that the yield without washing gradually increased with each cycle (Figure S11), eventually approaching the yield obtained after washing (Figure 5).

**Figure 6:**
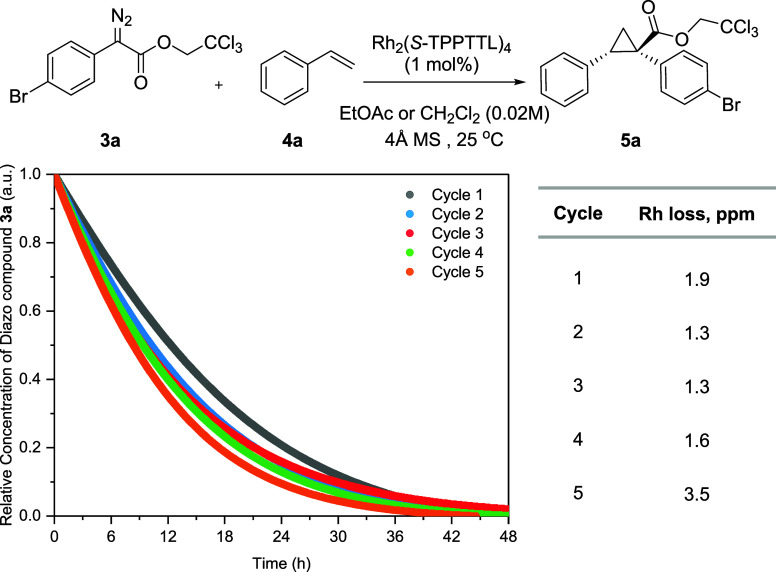
Kinetic studies for recycling reactions using the catalyst-in-bag system. ^a^Rhodium loss via permeation through the bag was determined by ICP-MS with internal standardization.

The robustness of the Bz-membrane during continuous use was evaluated by measuring the rhodium permeation and the reaction kinetics in EtOAc solvent (Figure 6). The rhodium content in the external reaction solutions in each of 5 cycles was measured using ICP-MS spectroscopy to detect the rhodium permeation from the bag to the outer solution surrounding the bag. From the first cycle to fifth cycle, similar rhodium permeation occurred, yielding rhodium concentrations of 1.9 to 3.5 ppm in the external solution. The kinetic profile measured via ReactIR also showed nearly identical reaction rates over the 5 cycles. These results demonstrate that the Bz-membrane maintains a robust catalyst-in-bag structure during the 5 cycles of reaction, allowing the catalyst to be stored and reused.

Catalyst recovery was carried out simply by cutting the used bag with scissors (Scheme 1D) and collecting the internal catalyst solution without further purification processes. The soluble rhodium recovery from the catalyst-in-bag system was measured at 91% in EtOAc as determined by H^1^ NMR experiments based on the initial catalyst loading after 5 cycles, and 9% of the rhodium loss was estimated to be retained on the membrane ends bound to the Teflon tape or lost during sample handling.

#### Deformability of the Catalyst-in-Bag System

**Figure 7:**
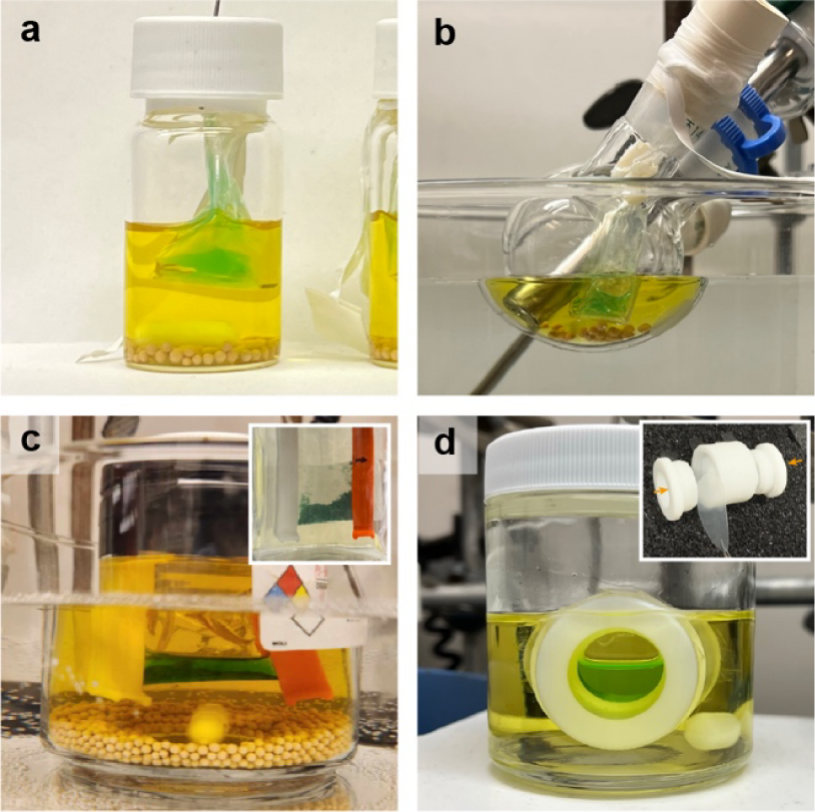
Deformability of catalyst-in-bag system. Reaction in (a) a 20 mL scale vial and (b) a 50 mL round-bottomed flask. (c) Reactions in 200 mL jar with enlarged catalyst-in-bag and (inset) the preparation of catalyst-in-bag with dialysis tubing clips as tube closures. (d) Reactions in catalyst-in-vessel as capsule reactor with disc-shaped membrane windows and (inset) unassembled reactor with arrows pointing to the windows.

The flexible nature of the Bz-membrane allows the customization and scaling of the chemical synthesis processes to suit specific reaction conditions by adjusting the bag size or converting the catalyst-in-bag system into various structures. As shown in Figure 7a,b, reactions proceed efficiently in different vessels using the same type of catalyst-in-bag, demonstrating its adaptability to various reaction environments. Scaling up the reaction is straightforward by simply increasing the size and enclosed volume of the membrane (Figures 7c and S13). In gram-scale experiments, the system maintained 83% yield and 92% ee in the cyclopropanation with no significant impact of scale on the performance. Additionally, after four washing steps, the rhodium catalyst was recovered in 99% yield with 92% of purity without further purification (Figure S14). Rhodium catalyst was also successfully deployed in a cylindrical, capsule reactor with disc-shaped membrane windows, demonstrating the versatility and adaptability of the system, allowing the use of a variety of structural morphologies, including catalysts-in-vessels beyond the traditional tube configuration that was the focus here (Figure 7d). However, cyclopropanation produces N_2_ gas as a byproduct, so for truly large-scale applications it will be necessary to engineer rapid gas permeation out of the catalyst-in-bag system to prevent membrane bursting during the initial fastest reaction rate period. Although an overall slow reaction rate is a limitation of this catalyst-in-bag design, this problem could be addressed by designing a suitable flow system in future work.

## Conclusions

In conclusion, we have developed a simplified method for the recycling and recovery of dirhodium catalysts in cyclopropanation reactions utilizing a catalyst-in-bag system. The kinetic analysis identified that substrate/product diffusion is the critical rate-limiting step in the Bz-membrane system. Although the use of nonoptimized commercial membranes slowed down the reaction rates, this catalyst-in-bag system with Rh_2_(*S*-TPPTTL)_4_ catalyst demonstrated compatibility with a variety of substrates and achieved comparable yields and enantioselectivities. Moreover, the catalyst-in-bag system is an interesting framework for enhancing precious metal and ligand recovery, as the bag is flexible, easy to store, and scalable. Further studies may advance this catalyst-in-bag system with improved reaction efficiency by developing more optimized membranes for specific target reactions like cyclopropanation.

## Experimental Section

### General Procedure for Membrane Preparation

The Bz-membrane (Sigma-Aldrich), cut to a length of 14 cm, was rinsed with distilled water and then underwent solvent exchange using distilled water for 30 min two times. This was followed by two 30 min immersions in methanol and hexane each. After these treatments, the membrane was dried under an argon atmosphere, sealed, and stored for further use.

### Representative Procedure for Cyclopropanation with Catalyst-in-Bag Design

One end of the dry membrane was sealed using chemically stable Teflon tape, and catalyst powder was introduced into the bag through a glass pipet. Then, the inside of the bag was purged with Ar gas to minimize air and moisture content that could cause side reactions. After sealing the other end, the prepared bag was stored in an Ar atmosphere to maintain its integrity. Before running a reaction, the dialysis membrane containing catalyst was soaked in 10 mL of dry ethyl acetate. In a flame-dried, 20 mL vial, equipped with a magnetic stir bar and 4 Å activated molecular sieves (∼100 mg, 100 wt %), styrene (1.0 mmol, 5.0 equiv, filter through silica plug to remove preservative) and diazo compound (0.200 mmol, 1.0 equiv) were added. After vacuuming and backfilling the vial with N_2_, 10 mL of dry ethyl acetate was added. The mixture was then stirred for 5 min before a dialysis bag from a separate soaking vial containing Rh_2_(*S*-TPPTTL)_4_ (5.00 mg, 1.0 mol %) catalyst was introduced into the first vial. The solution was stirred at 300 rpm overnight at room temperature. Upon reaction completion, while the solution was passed through a Celite filter to remove the molecular sieves, the dialysis membrane was immersed in another vial containing 10 mL ethyl acetate for 2 h to extract the products remaining inside the bag. The reaction mixture and washing mixture were then combined and concentrated under vacuum. The crude residue was purified by flash column chromatography.

## Supporting Information Available

Complete experimental procedures and compound characterization (PDF)

## References

[ref1] Davies H. M. L.; Manning J. R. Catalytic C–H functionalization by metal carbenoid and nitrenoid insertion. Nature 2008, 451 (7177), 417–424. 10.1038/nature06485. 18216847 PMC3033428

[ref2] Davies H. M. L.; Liao K. Dirhodium tetracarboxylates as catalysts for selective intermolecular C–H functionalization. Nat. Rev. Chem. 2019, 3 (6), 347–360. 10.1038/s41570-019-0099-x. 32995499 PMC7521778

[ref3] Davies H. M. L.; Nagashima T.; Klino J. L. Stereoselectivity of Methyl Aryldiazoacetate Cyclopropanations of 1,1-Diarylethylene. Asymmetric Synthesis of a Cyclopropyl Analogue of Tamoxifen. Org. Lett. 2000, 2 (6), 823–826. 10.1021/ol005563u. 10754686

[ref4] Ly D.; Bacsa J.; Davies H. M. L. Rhodium(II)-Catalyzed Asymmetric Cyclopropanation and Desymmetrization of [2.2]Paracyclophanes. ACS Catal. 2024, 14, 6423–6431. 10.1021/acscatal.4c01292. 38721377 PMC11075029

[ref5] Davies H. M. L.; Beckwith R. E. J. Catalytic Enantioselective C–H Activation by Means of Metal–Carbenoid-Induced C–H Insertion. Chem. Rev. 2003, 103 (8), 2861–2904. 10.1021/cr0200217. 12914484

[ref6] Nguyen T.-T. H.; Bosse A. T.; Ly D.; Suarez C. A.; Fu J.; Shimabukuro K.; Musaev D. G.; Davies H. M. L. Diaryldiazoketones as Effective Carbene Sources for Highly Selective Rh(II)-Catalyzed Intermolecular C–H Functionalization. J. Am. Chem. Soc. 2024, 146 (12), 8447–8455. 10.1021/jacs.3c14552. 38478893 PMC10979447

[ref7] Bien J.; Davulcu A.; DelMonte A. J.; Fraunhoffer K. J.; Gao Z.; Hang C.; Hsiao Y.; Hu W.; Katipally K.; Littke A.; et al. The First Kilogram Synthesis of Beclabuvir, an HCV NS5B Polymerase Inhibitor. Org. Process Res. Dev. 2018, 22 (10), 1393–1408. 10.1021/acs.oprd.8b00214.

[ref8] Wei B.; Sharland J. C.; Lin P.; Wilkerson-Hill S. M.; Fullilove F. A.; McKinnon S.; Blackmond D. G.; Davies H. M. L. In Situ Kinetic Studies of Rh(II)-Catalyzed Asymmetric Cyclopropanation with Low Catalyst Loadings. ACS Catal. 2020, 10 (2), 1161–1170. 10.1021/acscatal.9b04595.

[ref9] Sharland J. C.; Wei B.; Hardee D. J.; Hodges T. R.; Gong W.; Voight E. A.; Davies H. M. L. Asymmetric synthesis of pharmaceutically relevant 1-aryl-2-heteroaryl- and 1,2-diheteroarylcyclopropane-1-carboxylates. Chem. Sci. 2021, 12 (33), 11181–11190. 10.1039/D1SC02474D. 34522315 PMC8386643

[ref10] Toczko J. F. 9.9 Catalyst Recovery and Recycle: Metal Removal Techniques. In Comprehensive Chirality. Carreira E. M.; Yamamoto H., Eds.; Elsevier, 2012, pp 209–227.

[ref11] Nguyen T.-T. H.; Navarro A.; Ruble J. C.; Davies H. M. L. Stereoselective Synthesis of Either Exo- or Endo-3-Azabicyclo[3.1.0]hexane-6-carboxylates by Dirhodium(II)-Catalyzed Cyclopropanation with Ethyl Diazoacetate under Low Catalyst Loadings. Org. Lett. 2024, 26 (14), 2832–2836. 10.1021/acs.orglett.3c03652. 38166395 PMC11020159

[ref12] Chepiga K. M.; Feng Y.; Brunelli N. A.; Jones C. W.; Davies H. M. L. Silica-Immobilized Chiral Dirhodium(II) Catalyst for Enantioselective Carbenoid Reactions. Org. Lett. 2013, 15 (24), 6136–6139. 10.1021/ol403006r. 24251986

[ref13] Li Z.; Rösler L.; Wissel T.; Breitzke H.; Gutmann T.; Buntkowsky G. Immobilization of a chiral dirhodium catalyst on SBA-15 via click-chemistry: Application in the asymmetric cyclopropanation of 3-diazooxindole with aryl alkenes. J. CO2 Util. 2021, 52, 101682 10.1016/j.jcou.2021.101682.

[ref14] Yoo C.-J.; Rackl D.; Liu W.; Hoyt C. B.; Pimentel B.; Lively R. P.; Davies H. M. L.; Jones C. W. An Immobilized-Dirhodium Hollow-Fiber Flow Reactor for Scalable and Sustainable C–H Functionalization in Continuous Flow. Angew. Chem., Int. Ed. 2018, 57 (34), 10923–10927. 10.1002/anie.201805528. 29952054

[ref15] Hatridge T. A.; Liu W.; Yoo C.-J.; Davies H. M. L.; Jones C. W. Optimized Immobilization Strategy for Dirhodium(II) Carboxylate Catalysts for C–H Functionalization and Their Implementation in a Packed Bed Flow Reactor. Angew. Chem., Int. Ed. 2020, 59 (44), 19525–19531. 10.1002/anie.202005381. 32483912

[ref16] Adly F. G.; Ghanem A. Polymer monolith-supported dirhodium(II)-catalyzed continuous flow cyclopropanation in capillary format†. Tetrahedron Lett. 2016, 57 (8), 852–857. 10.1016/j.tetlet.2016.01.010.

[ref17] Liu J.; Plog A.; Groszewicz P.; Zhao L.; Xu Y.; Breitzke H.; Stark A.; Hoffmann R.; Gutmann T.; Zhang K.; et al. Design of a Heterogeneous Catalyst Based on Cellulose Nanocrystals for Cyclopropanation: Synthesis and Solid-State NMR Characterization. Chem. −Eur. J. 2015, 21 (35), 12414–12420. 10.1002/chem.201501151. 26179865

[ref18] Doyle M. P.; Yan M.; Gau H.-M.; Blossey E. C. Catalysts with Mixed Ligands on Immobilized Supports. Electronic and Steric Advantages. Org. Lett. 2003, 5 (4), 561–563. 10.1021/ol027475a. 12583769

[ref19] Doyle M. P.; Timmons D. J.; Tumonis J. S.; Gau H.-M.; Blossey E. C. Preparation and Catalytic Properties of Immobilized Chiral Dirhodium(II) Carboxamidates. Organometallics 2002, 21 (9), 1747–1749. 10.1021/om011082i.

[ref20] Takeda K.; Oohara T.; Anada M.; Nambu H.; Hashimoto S. A Polymer-Supported Chiral Dirhodium(II) Complex: Highly Durable and Recyclable Catalyst for Asymmetric Intramolecular C–H Insertion Reactions. Angew. Chem., Int. Ed. 2010, 49 (39), 6979–6983. 10.1002/anie.201003730. 20718063

[ref21] Li Z.; Rösler L.; Herr K.; Brodrecht M.; Breitzke H.; Hofmann K.; Limbach H.-H.; Gutmann T.; Buntkowsky G. Dirhodium Coordination Polymers for Asymmetric Cyclopropanation of Diazooxindoles with Olefins: Synthesis and Spectroscopic Analysis. ChemPluschem 2020, 85 (8), 1737–1746. 10.1002/cplu.202000421. 32790226

[ref22] Li Z.; Rösler L.; Wissel T.; Breitzke H.; Hofmann K.; Limbach H.-H.; Gutmann T.; Buntkowsky G. Design and characterization of novel dirhodium coordination polymers – the impact of ligand size on selectivity in asymmetric cyclopropanation. Catal. Sci. Technol. 2021, 11 (10), 3481–3492. 10.1039/D1CY00109D.

[ref23] Empel C.; Fetzer M. N. A.; Sasmal S.; Strothmann T.; Janiak C.; Koenigs R. M. Unlocking catalytic potential: A rhodium(ii)-based coordination polymer for efficient carbene transfer reactions with donor/acceptor diazoalkanes. Chem. Commun. 2024, 60 (57), 7327–7330. 10.1039/D4CC01386G. 38913109

[ref24] Li Z.; Jiang H.; Zhu M.; Zhang F. Self-Supported Chiral Dirhodium Organic Frameworks Enables Efficient Asymmetric Cyclopropanation. ACS Appl. Mater. Interfaces 2024, 16 (15), 19003–19013. 10.1021/acsami.4c02215. 38566322

[ref25] Davies H. M. L.; Walji A. M.; Nagashima T. Simple Strategy for the Immobilization of Dirhodium Tetraprolinate Catalysts Using a Pyridine-Linked Solid Support. J. Am. Chem. Soc. 2004, 126 (13), 4271–4280. 10.1021/ja0393067. 15053617

[ref26] Rösler L.; Höfler M. V.; Breitzke H.; Wissel T.; Herr K.; Heise H.; Gutmann T.; Buntkowsky G. Dirhodium complex immobilization on modified cellulose for highly selective heterogeneous cyclopropanation reactions. Cellulose 2022, 29 (11), 6283–6299. 10.1007/s10570-022-04654-y.

[ref27] Davies H. M. L.; Walji A. M. Asymmetric Intermolecular C–H Activation, Using Immobilized Dirhodium Tetrakis((S)-N-(dodecylbenzenesulfonyl)- prolinate) as a Recoverable Catalyst. Org. Lett. 2003, 5 (4), 479–482. 10.1021/ol0273506. 12583748

[ref28] Garlets Z. J.; Boni Y. T.; Sharland J. C.; Kirby R. P.; Fu J.; Bacsa J.; Davies H. M. L. Design, Synthesis, and Evaluation of Extended C 4 −Symmetric Dirhodium Tetracarboxylate Catalysts. ACS Catal. 2022, 12 (17), 10841–10848. 10.1021/acscatal.2c03041. 37274599 PMC10237630

[ref29] Liao K.; Yang Y.-F.; Li Y.; Sanders J. N.; Houk K. N.; Musaev D. G.; Davies H. M. L. Design of catalysts for site-selective and enantioselective functionalization of non-activated primary C–H bonds. Nat. Chem. 2018, 10 (10), 1048–1055. 10.1038/s41557-018-0087-7. 30082883 PMC6650386

[ref30] Gaab M.; Bellemin-Laponnaz S.; Gade L. H. ″Catalysis in a tea bag″: Synthesis, catalytic performance and recycling of dendrimer-immobilised bis- and trisoxazoline copper catalysts. Chemistry 2009, 15 (22), 5450–5462. 10.1002/chem.200900504. 19388035

[ref31] Willemsen J. S.; van Hest J. C. M.; Rutjes F. P. J. T. Aqueous reductive amination using a dendritic metal catalyst in a dialysis bag. Beilstein J. Org. Chem. 2013, 9, 960–965. 10.3762/bjoc.9.110. 23766812 PMC3678615

[ref32] Pijnenburg N. J. M.; Dijkstra H. P.; van Koten G.; Gebbink R. J. M. K. SCS-pincer palladium-catalyzed auto-tandem catalysis using dendritic catalysts in semi-permeable compartments. Dalton Trans. 2011, 40 (35), 8896–8905. 10.1039/c1dt10502g. 21731937

[ref33] Larsen C. R.; Paulson E. R.; Erdogan G.; Grotjahn D. B. A. Facile, Convenient, and Green Route to (E)-Propenylbenzene Flavors and Fragrances by Alkene Isomerization. Synlett 2015, 26 (17), 2462–2466. 10.1055/s-0035-1560205.

[ref34] Krupková A.; Müllerová M.; Petrickovic R.; Strašák T. On the edge between organic solvent nanofiltration and ultrafiltration: Characterization of regenerated cellulose membrane with aspect on dendrimer purification and recycling. Sep. Purif. Technol. 2023, 310, 123141 10.1016/j.seppur.2023.123141.

[ref35] MillerIi A. L.; Bowden N. B. Site-Isolation and Recycling of PdCl2 using PDMS Thimbles. J. Org. Chem. 2009, 74 (13), 4834–4840. 10.1021/jo900570y. 19558181

[ref36] Clausi D. T.; Koros W. J. Formation of defect-free polyimide hollow fiber membranes for gas separations. J. Membr. Sci. 2000, 167 (1), 79–89. 10.1016/S0376-7388(99)00276-8.

