# Supplementary material for: Facile Recovery and Recycling of a Soluble Dirhodium Catalyst in Asymmetric Cyclopropanation via a Catalyst-in-Bag System
Source: Org Process Res Dev. 2024 Oct 23;28(11):4146–55. doi: 10.1021/acs.oprd.4c00400 (PMC11574847; doi:10.1021/acs.oprd.4c00400)
Supplement: Supplementary file 1 — op4c00400_si_001.pdf [file op4c00400_si_001.pdf]

Supporting Information for:

Facile Recovery and Recycling of a Soluble Dirhodium Catalyst in Asymmetric Cyclopropanation via a Catalyst-in-Bag System

UnJin Ryu,[‡,1] *Duc Ly,*[‡,2] *Kristin Shimabukuro,*[2] *Huw M. L. Davies**[,2] *and Christopher W. Jones**[,1]

[1]School of Chemical & Biomolecular Engineering, Georgia Institute of Technology, Atlanta, GA 30332, United States

[2]Department of Chemistry, Emory University, Atlanta, Georgia 30322, United States

Email: hmdavie@emory.edu; cjones@chbe.gatech.edu

Table of Contents

1. Materials and general methods 3
2. Preparation of the catalyst-in-bag system 5
   2.1. Bz-membrane preparation 5
   2.2. Catalyst-in-bag fabrication 6
   2.3. Feasibility of catalyst-in-bag system 7
   2.4. General procedure A: Cyclopropanation with Catalyst-in-bag 9
3. Preparation of ReactIR for kinetic studies 10
   3.1. General procedure B: ReactIR setup and cyclopropanation with the catalyst-in bag system 10
   3.2. Effect of Reaction Temperature 12
   3.3. Effect of Different Catalyst Loadings 15
   3.4. Effect of Different Substrate Concentrations 20
4. Compatibility Tests 23
5. Recycle procedure with the Catalyst-in-Bag 24
   5.1. Ethyl acetate 26
   5.2. Dichloromethane 28
6. Scale-up Experiments 31
7. Preparation of substrates 34
8. Characterization of cyclopropanation products 35
9. Copies of NMR spectra 52

1. Materials and general methods

1.1. Materials

Unless otherwise noted, all other reagents were obtained from commercial sources (Sigma Aldrich, Fisher, TCI Chemicals, AK Scientific, Combi Blocks, Oakwood Chemicals, Ambeed) and used as received without purification. The benzoylated tubing with 2k MWCO and 32 mm flat width, was purchased from Sigma Aldrich. The regenerated cellulose dry dialysis tubing with 3 kDa, 8 kDa MWCO was purchased from Spectrum™ Labs.

1.2. Methods

All experiments were carried out in flame-dried glassware under an argon atmosphere unless otherwise stated. Flash column chromatography was performed on silica gel. Unless otherwise noted, all other reagents were obtained from commercial sources (Sigma Aldrich, Fisher, TCI Chemicals, AK Scientific, Combi Blocks, Oakwood Chemicals, Ambeed) and used as received without purification. ^{1}H and ^{13}C NMR spectra were recorded at either 400 MHz (^{13}C at 100 MHz) on Bruker 400 spectrometer or 600 MHz (^{13}C at 151 MHz) on INOVA 600 or Bruker 600 spectrometers. NMR spectra were run in solutions of deuterated chloroform ($CDCl_3$) with residual chloroform taken as an internal standard (7.26 ppm for ^{1}H, and 77.16 ppm for ^{13}C), and were reported in parts per million (ppm). The abbreviations for multiplicity are as follows: s = singlet, d = doublet, t = triplet, q = quartet, p = pentet, m = multiplet, dd = doublet of doublet, etc. Coupling constants (J values) were obtained from the spectra. Thin layer chromatography was performed on aluminum-back silica gel plates with UV light and cerium aluminum molybdate (CAM) stain to visualize. Mass spectra were taken on a Thermo Finnigan LTQ-FTMS spectrometer with APCI or ESI. Melting points (mp) were measured in open capillary tubes with a Mel-Temp Electrothermal melting points apparatus and are uncorrected. ICP-MS analyses were carried out by Center for

Applied Isotope Studies (University of Georgia) with Thermo Scientific X Series II ICP-MS. IR spectra were collected on a Nicolet iS10 FT-IR spectrometer from Thermo Scientific and reported in unit of cm^{-1}. Enantiomeric excess (% ee) data were obtained on an Agilent 1100 HPLC, eluting the purified products using a mixed solution of HPLC-grade 2-propanol (i-PrOH) and n-hexane. HPLC traces are reported based on the racemic retention times. The HPLC instruments used isopropanol/hexane gradient and commercial ChiralPak/ChiralCel columns from Daicel Chemical Industries, notably ChiralPak AD-H (5 µm particle size, 4.6 mm vs. 250 mm), ChiralCel OZ-H (5 µm particle size, 4.6 mm vs. 250 mm), and ChiralCel OD-H (5 µm particle size, 4.6 mm vs. 250 mm), ChiralCel AS-H (5 µm particle size, 4.6 mm vs. 250 mm), ChiralCel OJ-H (5 µm particle size, 4.6 mm vs. 250 mm), and Regis (S,S) Whelk-O1 5/100 Kromasil.

2. Preparation of the catalyst-in-bag system

2.1. Bz-membrane preparation

The Bz-membrane (Sigma-Aldrich), cut to a length of 14 cm, was rinsed with distilled water and then underwent solvent exchange using distilled water for 30 min two times. This was followed by two 30 min immersions in methanol and hexane each. After these treatments, the membrane was dried under an argon atmosphere, sealed, and stored for further use.

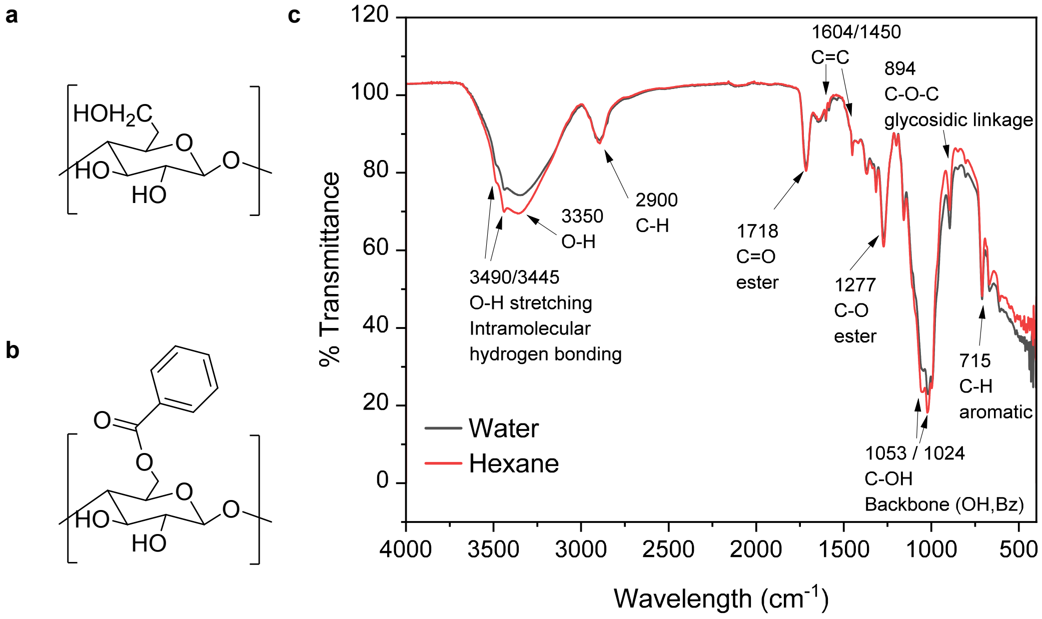

Figure S1. Cellulose structure of RC-and Bz-membranes. Glucose unit of (a) regenerated cellulose (RC) structure and (b) benzoylated cellulose (Bz). (c) FT-IR spectra of dried Bz-membrane after solvent exchange with water and hexane.

2.2. Catalyst-in-bag fabrication

One end of the dry membrane was sealed using chemically stable Teflon tape, and catalyst powder was introduced into the bag through a glass pipette. Then, the inside of the bag was purged with Ar gas to minimize air and moisture content that could cause side reactions. After sealing the other end, the prepared bag was stored in an Ar atmosphere to maintain its integrity.

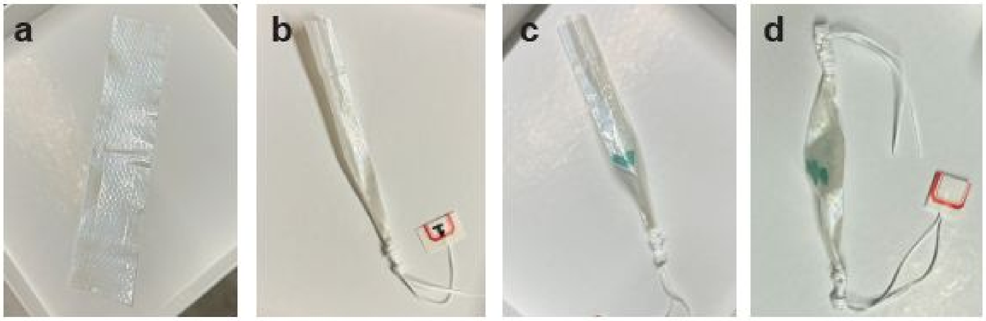

Figure S2. The process of catalyst-in-bag fabrication. (a) Dried membrane. (b) Membrane bag sealed at one end. (c) Bz-membrane with catalyst powder introduced through a glass pipette. (d) Completed catalyst-in-bag by sealing the other end using Teflon tape.

2.3. Feasibility of catalyst-in-bag system

Solvent permeability tests were performed using a catalyst-in-bag containing 5 mg of Rh_2(*S*-TPPTTL)$_4$. The catalyst-in-bag system was prepared using three types of membranes: 3 kDa and 8 kDa MWCO regenerated cellulose (RC) membranes, and a 2 kDa MWCO benzoylated (Bz) cellulose membrane. After soaking in 12 mL of ethyl acetate (EtOAc) and dichloromethane (CH_2Cl_2) for 24 hours, we assessed the degree of wetness of the catalyst within the membrane (**Figure S3a-b**).

Permeability tests for diazoacetate were conducted using a diazo-in-bag setup, each containing 0.2 mL of a diazo acetate solution with 24 mg of diazoacetate in either EtOAc or CH_2Cl_2. After soaking in 12 mL of EtOAc and CH_2Cl_2 for 1 day, the amount of diazoacetate that diffused into the outer solvent was analyzed (**Figure S3c-d**). The diffusion (%) relative to the original amount (24 mg) was calculated using ^{1}H NMR with n-dodecane as an internal standard.

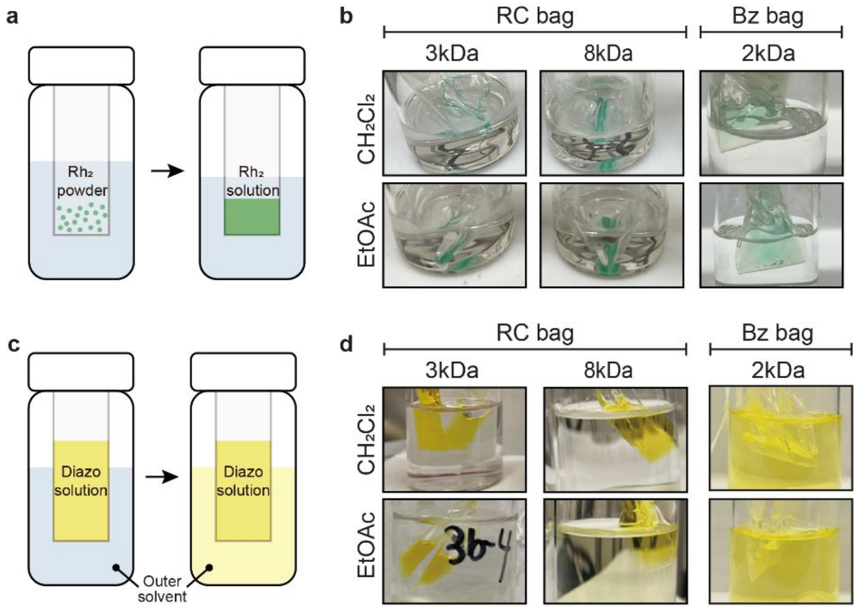

Figure S3. Feasibility experiments of bag system by (a-b) solvent permeability and (c-d) substrate permeability.

Table S1. Summary of permeability experiments with diazo compound 3a.

Solvent	Membrane	Color change[1]	Diffusion (%)[2]
	RC 3kDa	n/a	n/a
Dichloromethane (CH_2Cl_2)	RC 8kDa	n/a	n/a
	Bz 2kDa	Yellow	63
	RC 3kDa	n/a	0.3
Ethyl Acetate (EtOAc)	RC 8kDa	Yellow	11
	Bz 2kDa	Yellow	66

[1] Color change of outer solvent after 1 day soaking of diazo-in-bag. [2] Diffusion (%) of diazo acetate from the bag to outer solvent, as analyzed by ^1H NMR. The theoretical chemical equilibrium of diffusion is expected to be 92% outside the bag.

2.4. General procedure A: Cyclopropanation with Catalyst-in-bag

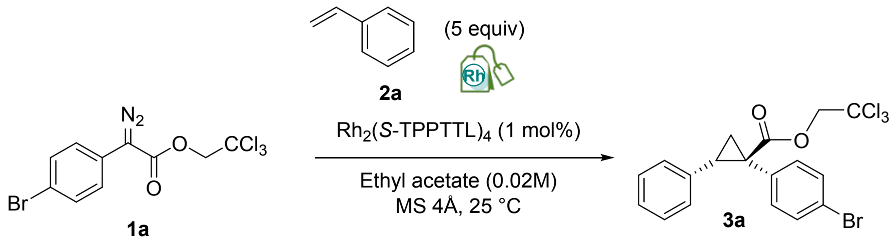

Before running a reaction, the dialysis membrane containing catalyst was soaked in 10 mL of dry ethyl acetate. In a flame-dried, 20 mL vial, equipped with a magnetic stir bar and 4Å activated molecular sieves (~100 mg, 100 wt%), styrene (115 μL, 1.0 mmol, 5.0 equiv, filter through silica plug to remove preservative) and 2,2-trichloroethyl 2-(4-bromophenyl)-2-diazoacetate (74.5 mg, 0.200 mmol, 1.0 equiv.) were added. After vacuuming and back-filling the vial with N_2, 10 mL of dry ethyl acetate (distilled over CaH_2, stored with MS 4Å) was added. The mixture was then stirred for 5 min before a dialysis bag from a separate soaking vial containing Rh_2(*S*-TPPTTL)$_4$ (5.00 mg, 1.0 mol%) catalyst was introduced into the first vial. The stirring rate was maintained at 300 rpm throughout the experiment. The solution was stirred overnight at room temperature. Upon reaction completion, while the solution was passed through a celite filter to remove the molecular sieves, the dialysis membrane was immersed in another vial containing 10 mL ethyl acetate for 2 hours to extract the products remaining inside the bag. The reaction mixture and washing mixture were then combined and concentrated under vacuum. The crude residue was purified by flash column chromatography.

3. Preparation of ReactIR for kinetic studies

3.1. General procedure B: ReactIR setup and cyclopropanation with the catalyst-in bag system

The ReactIR instrument was filled with liquid nitrogen and allowed to equilibrate while the reaction flask was being set-up. An oven-dried 25 mL 3-neck round-bottom flask with 0.2 g 4 Å molecular sieves was fitted with a rubber septum (left neck, 14/20), ReactIR probe (center neck, 24/40 to 19/25 adapter, 19/25 neck), and a rubber septum (right neck, 14/20) (**Figure S4**).

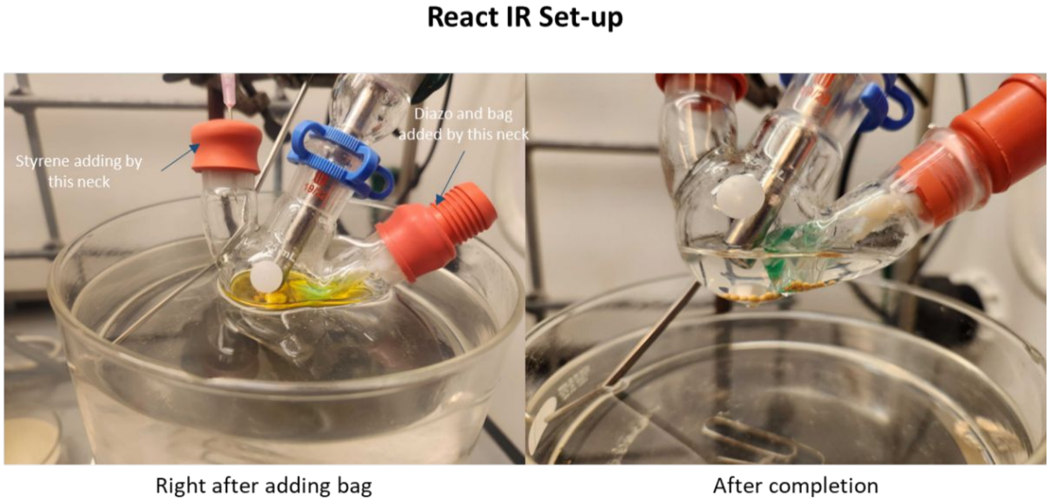

Figure S4. Illustration of the ReactIR apparatus.

An oven dried three-neck round bottom flask equipped with a stir bar and 4Å molecular sieves was fitted to the React IR 45m probe, cooled under vacuum, then backfilled with nitrogen 3 times and placed in an oil bath **(Figure S4**). The flask was then heated to the desired temperature (room temperature, 40 ^{o}C, or 60 ^{o}C). After the oil bath reached the desired temperature, the background and water vapor spectrum were taken via the ReactIR instrument. Ethyl acetate (10 mL) was added to the flask by a syringe through a rubber septum, the stirring rate was maintained at 300 rpm

throughout the experiment then the flask was left to equilibrate for 15 min before starting data collection. After 10 min, styrene (115 μL, 1.0 mmol, 5 equiv) was injected into the flask. 2,2,2-trichloroethyl 2-(4-bromophenyl)-2-diazoacetate (0.200 mmol) was also added to the flask sequentially after the addition of styrene for 5 min, and the flask was left to equilibrate for 1 hour. The React IR was set to monitor the diazo stretching vibration at 2096 cm^{-1}. After the equilibration, the dialysis membrane (containing Rh_2(*S*-TPPTTL)$_4$ at 0.1 to 5 mol %) that was soaked a day before the reaction in distilled ethyl acetate, was placed inside in the flask in a way that the stir bar could not touch the membrane. After completion of the reaction (monitored by the disappearance of the yellow color of the solution and of the peak at 2196 cm^{-1}), the reaction media were concentrated and purified through flash chromatography (0% hexanes/diethyl ether, 0-10% hexanes/diethyl ether) to afford a colorless oil, which gradually turned into a crystalline solid.

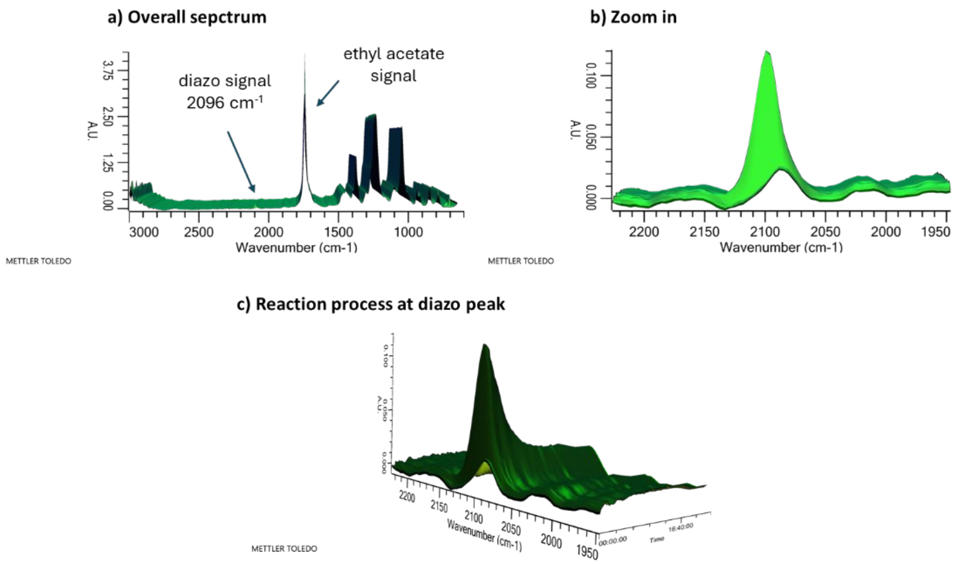

Figure S5. The characteristic peak of a diazo compound (2096 cm^{-1}) monitored by ReactIR in the reaction process. (a) Overall IR spectrum of the reaction mixture. (b) Zoomed-in spectra for the diazo signal. (c) Illustration of the disappearance of the diazo signal at 2096 cm^{-1}.

3.2. Effect of Reaction Temperature

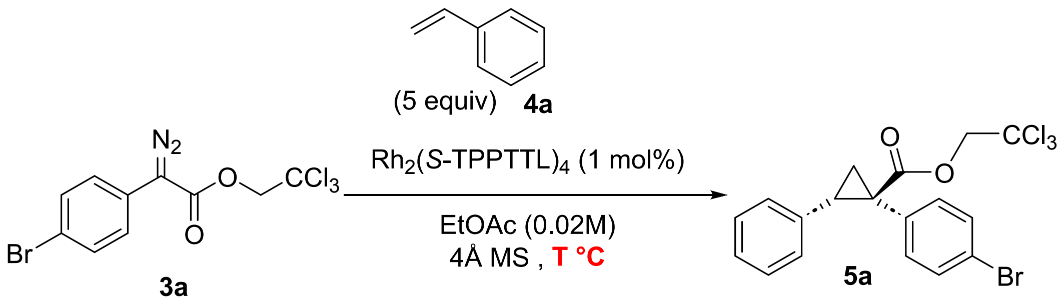

The experiments were conducted according to general procedure B for ReactIR studies. The reaction was carried out using styrene (115 μL, 1.0 mmol, 5 equiv), 2,2,2-trichloroethyl 2-(4-bromophenyl)-2-diazoacetate (0.200 mmol), Rh_2(*S*-TPPTTL)$_4$ (5.0 mg, 1.0 mol%) and 4Å molecular sieves (100 wt%) in 10 mL of dry ethyl acetate (0.02 M) at room temperature, 40 ^{o}C, or 60 ^{o}C.

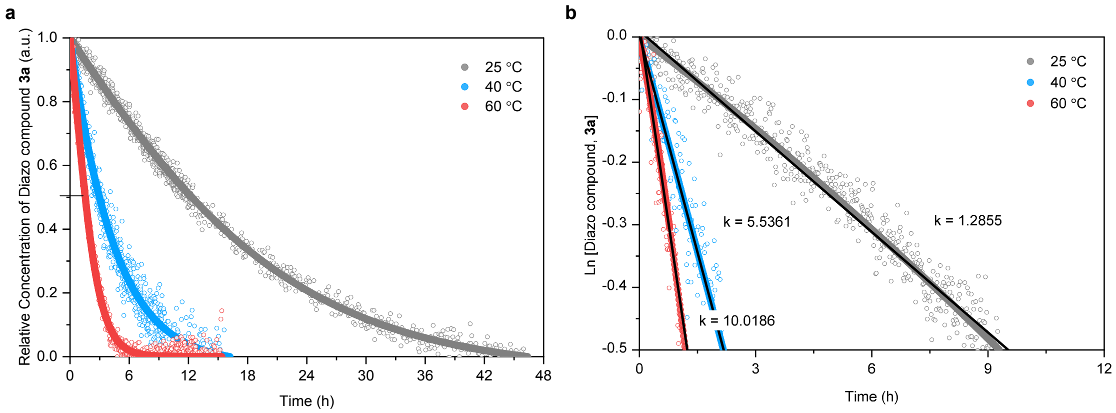

Figure S6. ReactIR study on the effect of temperature. (a) Kinetic cyclopropanation profiles using the catalyst-in-bag system at different temperatures (25 °C, 40 °C and 60 °C). (b) Comparison of the reaction rate coefficient at 25 °C, 40 °C and 60 °C. **(**The empty circles represent the raw data, while the solid circles display the Boltzmann fitting results, which are utilized in the main text. Black line is a linear fitting.)

HPLC traces

3.2.1. 25 ^{o}C (94% ee)

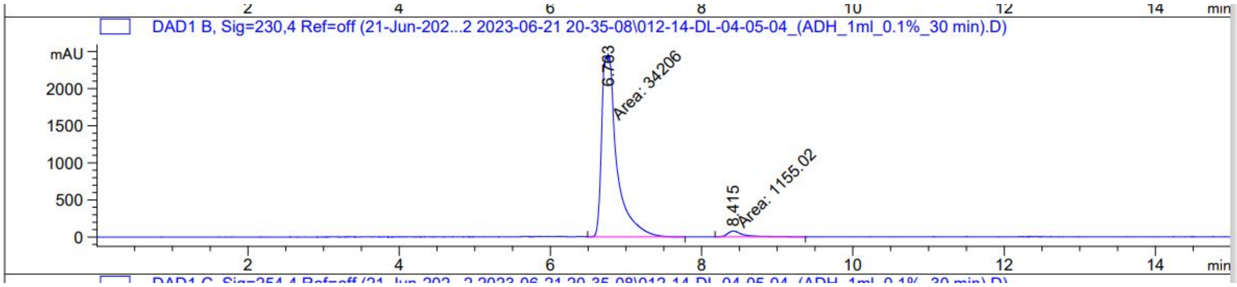

3.2.2. 40 ^{o}C (92% ee)

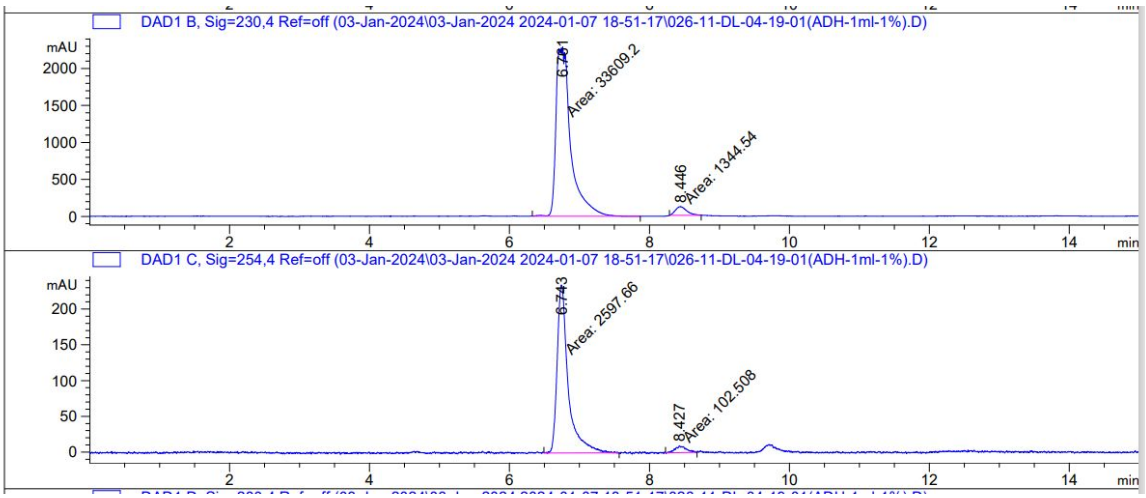

Signal 2: DAD1 B, Sig=230,4 Ref=off

Peak #	RetTime [min]	Type	Width [min]	Area [mAU*s]	Height [mAU]	Area %
1	6.761	MM	0.2445	3.36092e4	2290.80786	96.1534
2	8.446	MM	0.1895	1344.54358	118.24394	3.8466
Totals :				3.49538e4	2409.05180	

3.2.3. 60 °C (87% ee)

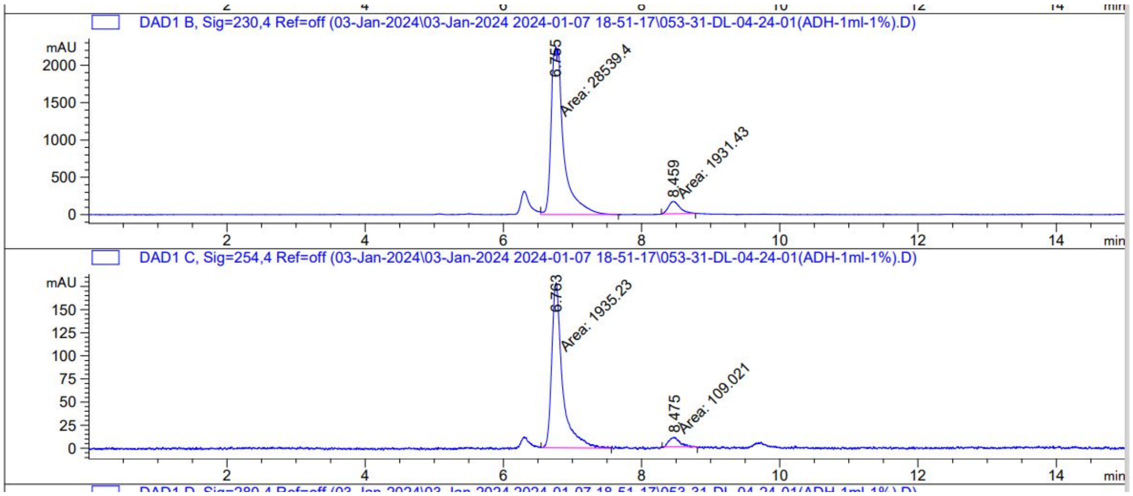

Signal 2: DAD1 B, Sig=230,4 Ref=off

Peak #	RetTime [min]	Type	Width [min]	Area [mAU*s]	Height [mAU]	Area %
1	6.755	MM	0.2121	2.85394e4	2242.32129	93.6614
2	8.459	MM	0.1950	1931.42566	165.12073	6.3386

Totals : 3.04708e4 2407.44202

3.3. Effect of Different Catalyst Loadings

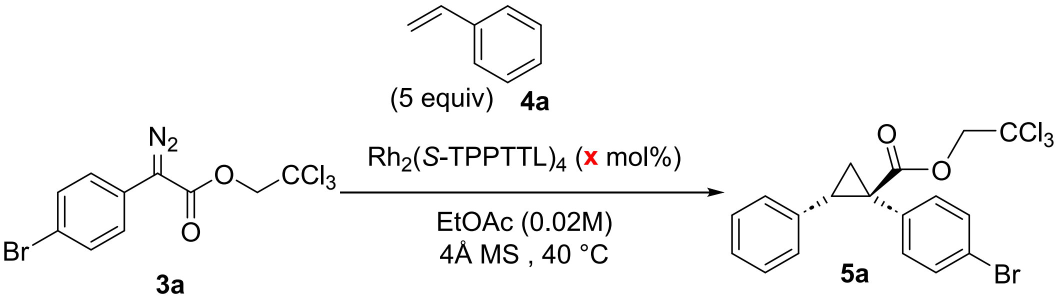

The experiments were conducted according to general procedure B for ReactIR studies. The reaction was carried out using styrene (115 μL, 1.0 mmol, 5 equiv), 2,2,2-trichloroethyl 2-(4-bromophenyl)-2-diazoacetate (0.200 mmol), and 4Å molecular sieve (100 wt%) in 10 mL of dried ethyl acetate (0.02 M) at 40 °C with varied Rh_2(*S*-TPPTTL)$_4$ loadings ranging from 0.1 to 5 mol%.

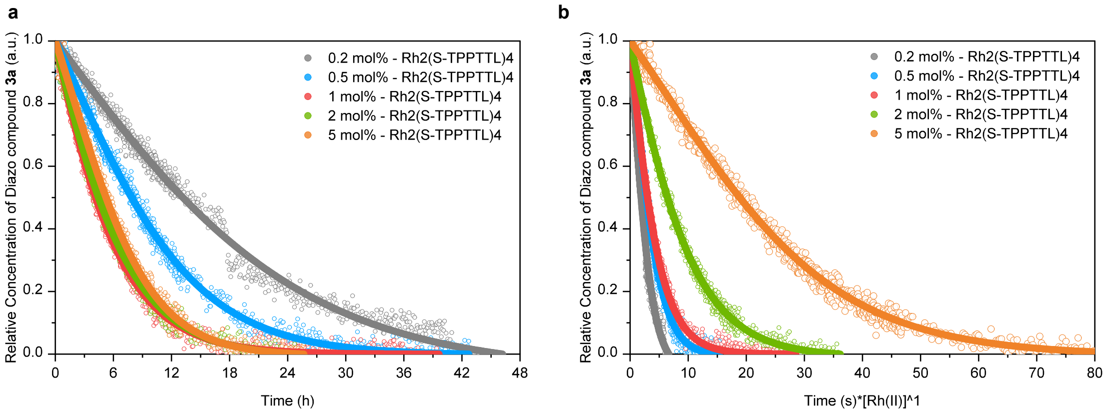

Figure S7. ReactIR study on the effect of catalyst loading. (a) Kinetic cyclopropanation profiles using the catalyst-in-bag system with different catalyst loading (0.2 mol%, 0.5 mol%, 1 mol%, 2 mol% and 5 mol%). (b) The variable time normalization analysis (VTNA) experiments when n=1. (The empty circles represent the raw data, while the solid circles display the Boltzmann fitting results, which are utilized in the main text.)

3.3.1. 0.1 mol% (87% ee)

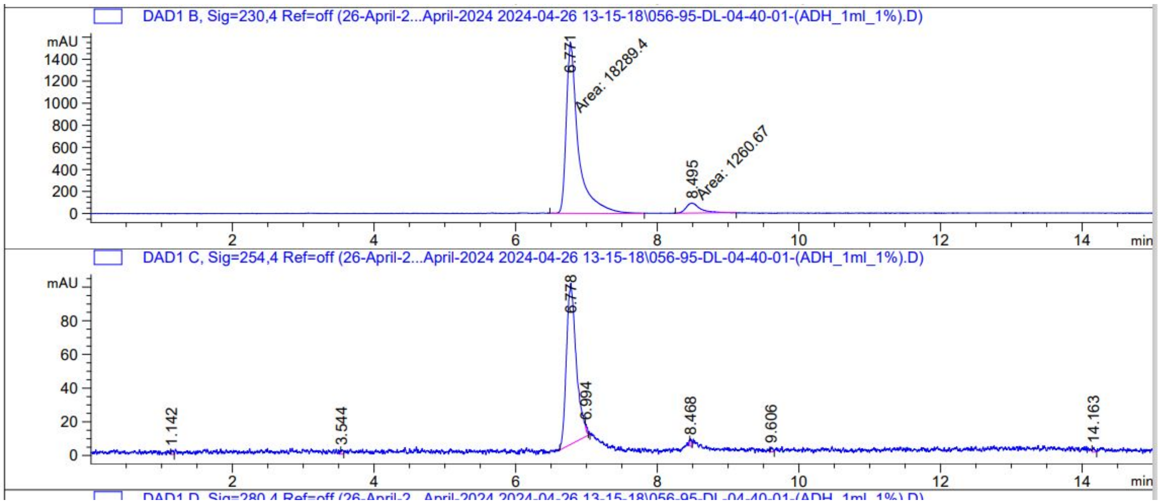

Signal 2: DAD1 B, Sig=230,4 Ref=off

Peak #	RetTime [min]	Type	Width [min]	Area [mAU*s]	Height [mAU]	Area %
1	6.771	MM	0.1960	1.82894e4	1555.20496	93.5516
2	8.495	MM	0.2304	1260.66858	91.17696	6.4484

Totals : 1.95500e4 1646.38191

3.3.2. 0.5 mol% (92% ee)

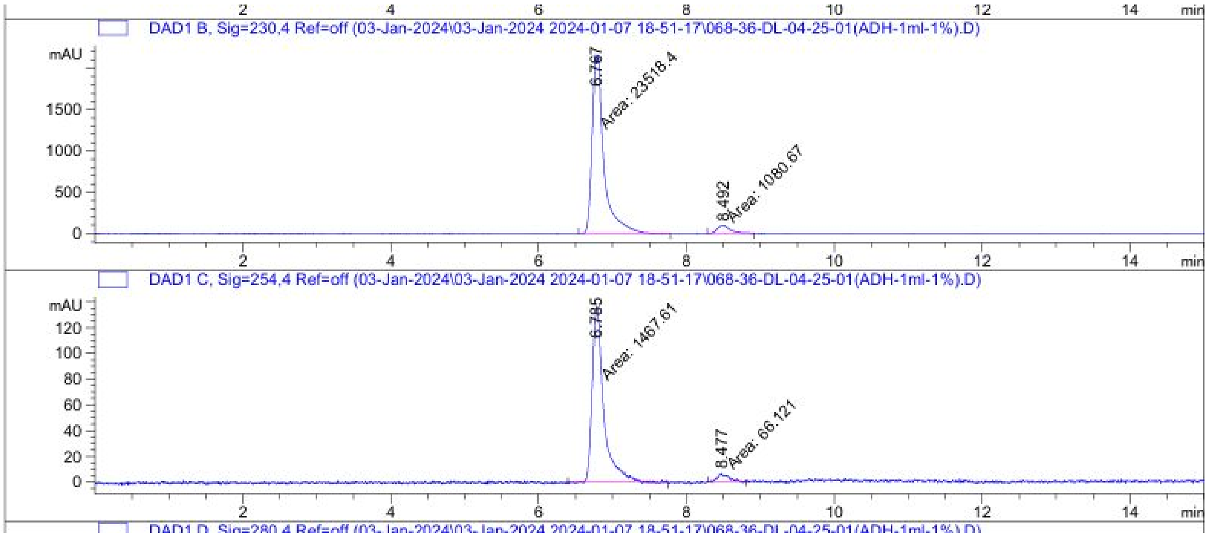

Signal 2: DAD1 B, Sig=230,4 Ref=off

Peak #	RetTime [min]	Type	Width [min]	Area [mAU*s]	Height [mAU]	Area %
1	6.767	MM	0.1810	2.35184e4	2165.04712	95.6068
2	8.492	MM	0.1999	1080.67480	90.10506	4.3932
Totals :				2.45991e4	2255.15218	

3.3.3. 1 mol% (92% ee)

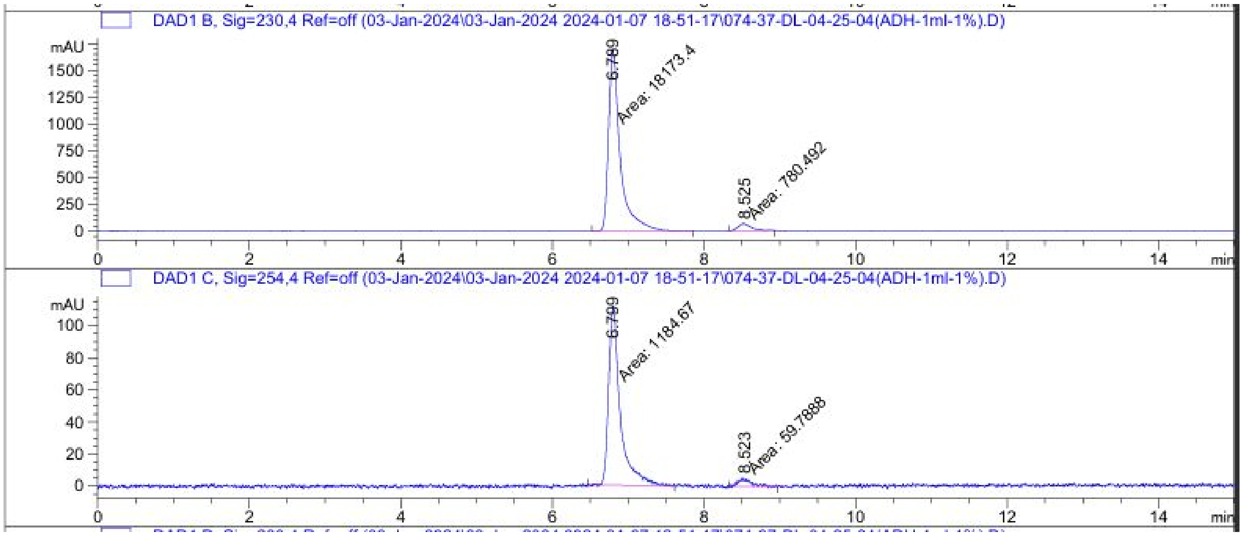

Signal 2: DAD1 B, Sig=230,4 Ref=off

Peak #	RetTime [min]	Type	Width [min]	Area [mAU*s]	Height [mAU]	Area %
1	6.789	MM	0.1769	1.81734e4	1712.12048	95.8822
2	8.525	MM	0.1969	780.49152	66.05424	4.1178
Totals :				1.89539e4	1778.17473	

3.3.4. 2.0 mol% (91% ee)

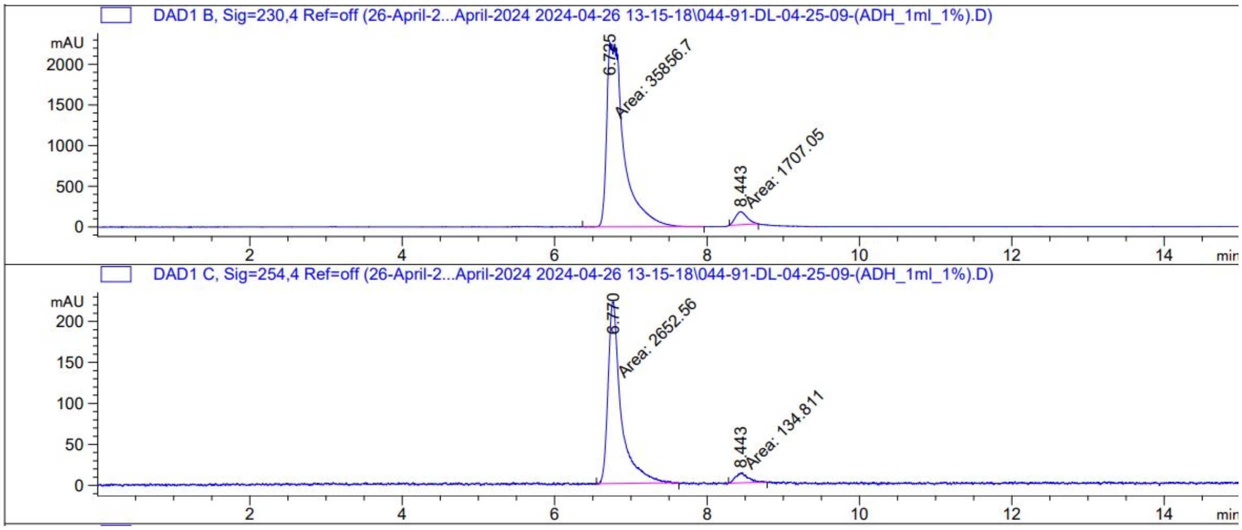

Signal 2: DAD1 B, Sig=230,4 Ref=off

Peak #	RetTime [min]	Type	Width [min]	Area [mAU*s]	Height [mAU]	Area %
1	6.725	MM	0.2636	3.58567e4	2267.09692	95.4556
2	8.443	MM	0.1779	1707.04675	159.94730	4.5444
Totals :				3.75638e4	2427.04422	

3.3.5. 5.0 mol% (92% ee)

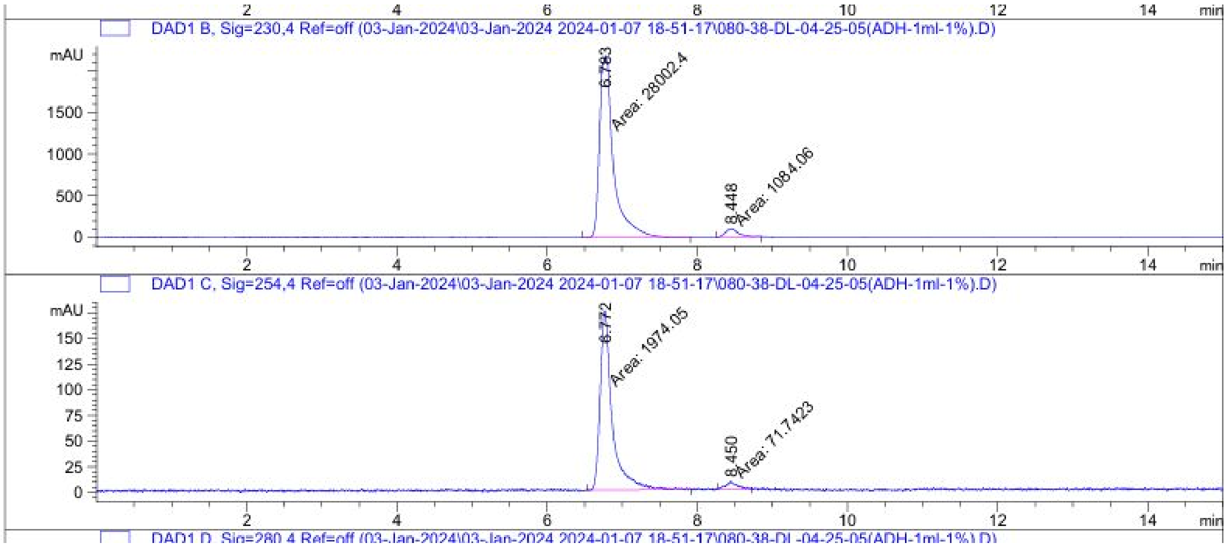

Signal 2: DAD1 B, Sig=230,4 Ref=off

Peak #	RetTime [min]	Type	Width [min]	Area [mAU*s]	Height [mAU]	Area %
1	6.783	MM	0.2138	2.80024e4	2182.52002	96.2730
2	8.448	MM	0.1901	1084.05884	95.04501	3.7270

Totals : 2.90865e4 2277.56503

3.4. Effect of Different Substrate Concentrations

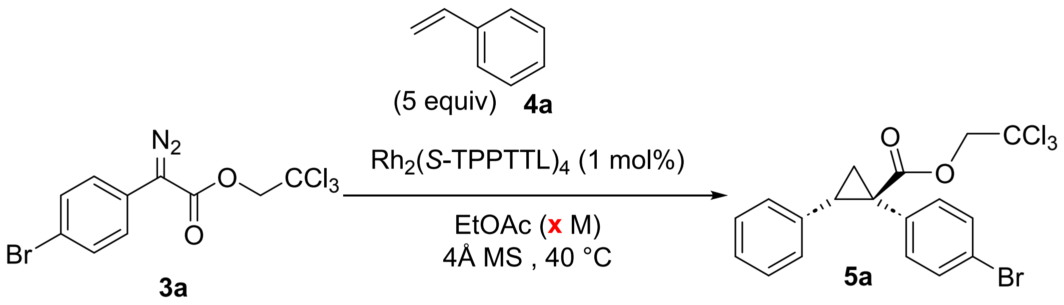

The experiments were conducted according to general procedure B for ReactIR studies. The reaction was carried out using styrene (5 equiv), 2,2,2-trichloroethyl 2-(4-bromophenyl)-2-diazoacetate (0.2 mmol), Rh_2(*S*-TPPTTL)$_4$ (1.0 mol%) and 4Å molecular sieve (100 wt%) in 10 mL of dried ethyl acetate (0.02M) at 40 ^{o}C with varied diazo concentrations ranging from 0.01 M to 0.1 M.

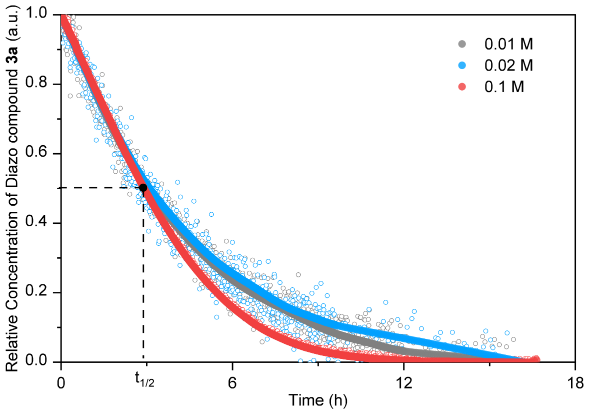

Figure S8. ReactIR study on the effect of substrates concentration. (The empty circles represent the raw data, while the solid circles display the Boltzmann fitting results, which are utilized in the main text.)

3.4.1. 0.01 M – 92% ee

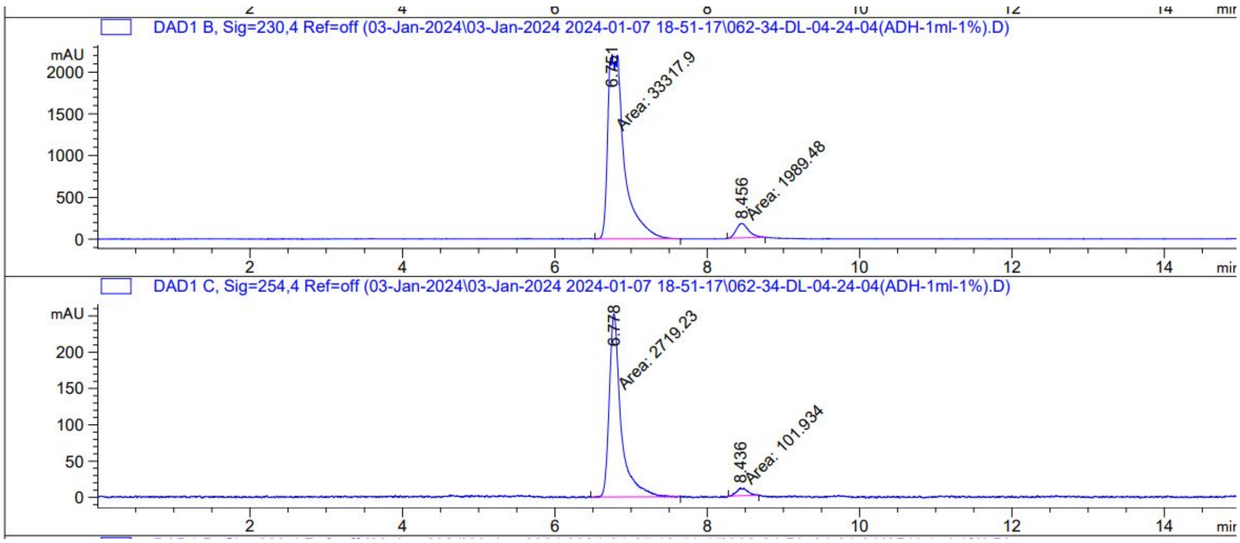

Signal 3: DAD1 C, Sig=254,4 Ref=off

Peak #	RetTime [min]	Type	Width [min]	Area [mAU*s]	Height [mAU]	Area %
1	6.778	MM	0.1795	2719.22949	252.48961	96.3868
2	8.436	MM	0.1548	101.93395	10.97344	3.6132

Totals :				2821.16344	263.46305	

3.4.2. 0.04 M – 92% ee

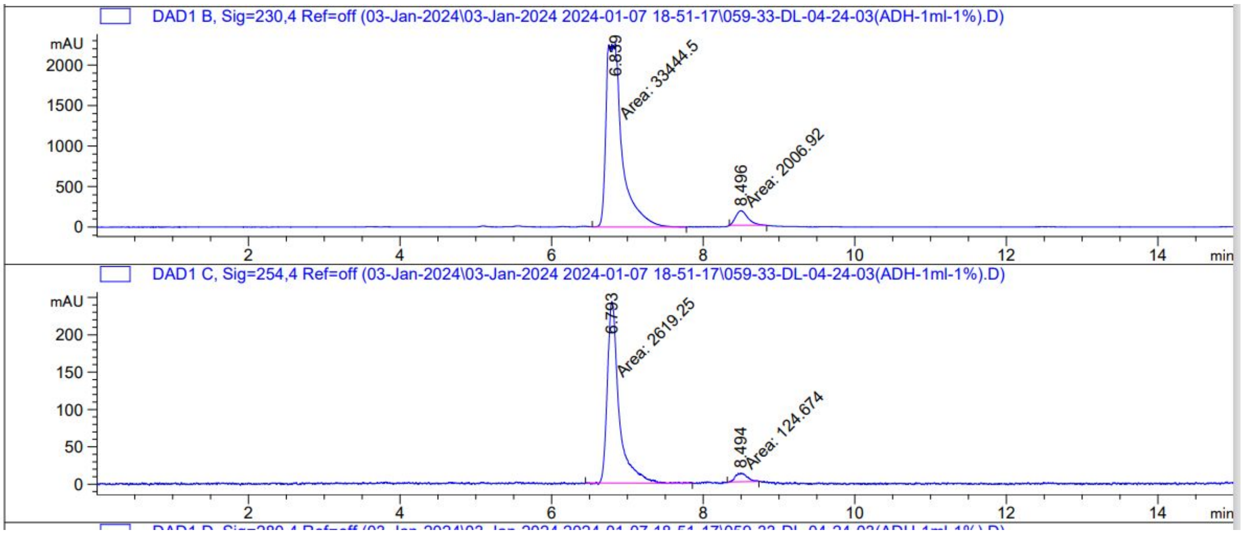

Signal 3: DAD1 C, Sig=254,4 Ref=off

Peak #	RetTime [min]	Type	Width [min]	Area [mAU*s]	Height [mAU]	Area %
1	6.793	MM	0.1795	2619.25122	243.20100	95.4563
2	8.494	MM	0.1727	124.67441	12.03369	4.5437
Totals :				2743.92563	255.23469	

3.4.3. 0.10 M – 92% ee

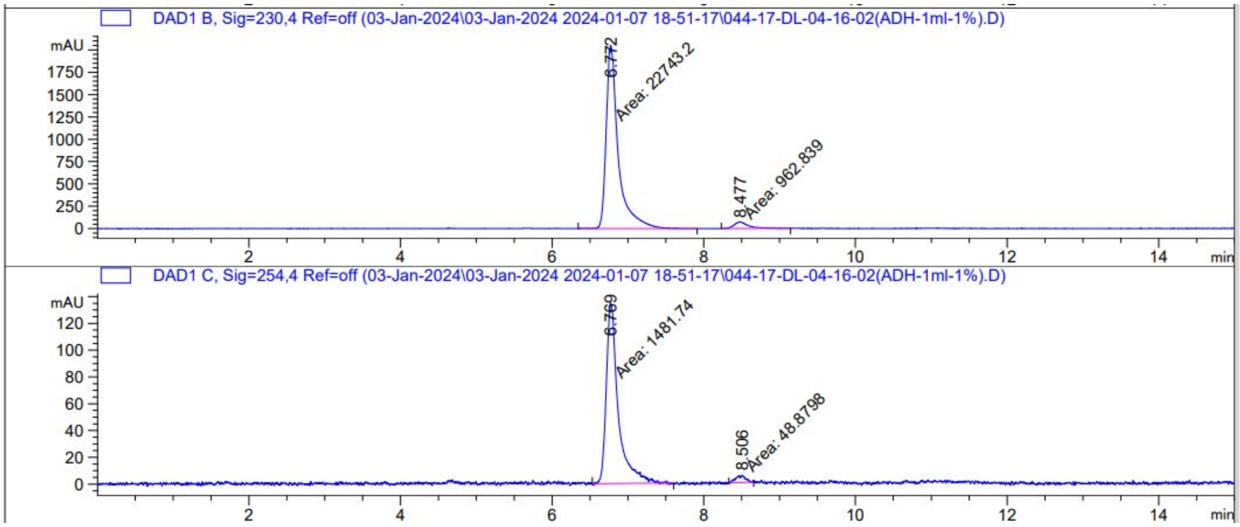

4. Compatibility Tests

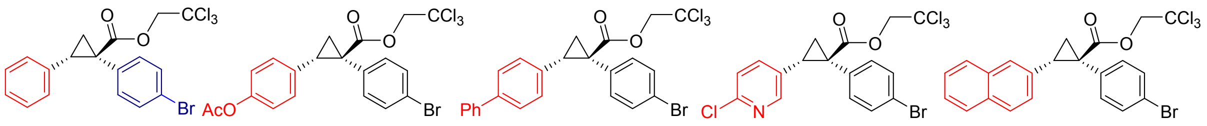

5a 75% (93% ee) | **5b** 82% (96% ee) | **5c** 68% (97% ee) | **5d** 58% (95% ee) | **5e** 65% (95% ee)

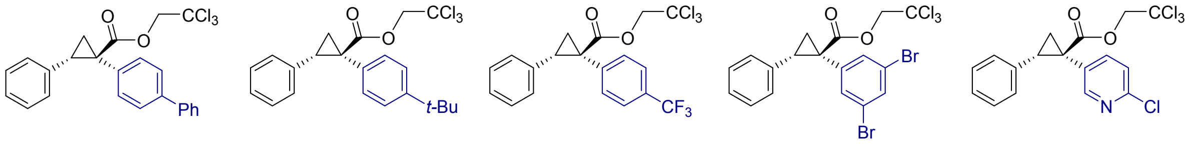

5f 76% (83% ee) | **5g** 63% (76 % ee) | **5h** 72% (89 % ee) | **5i** 64% (20 % ee) | **5j** 71% (90% ee)

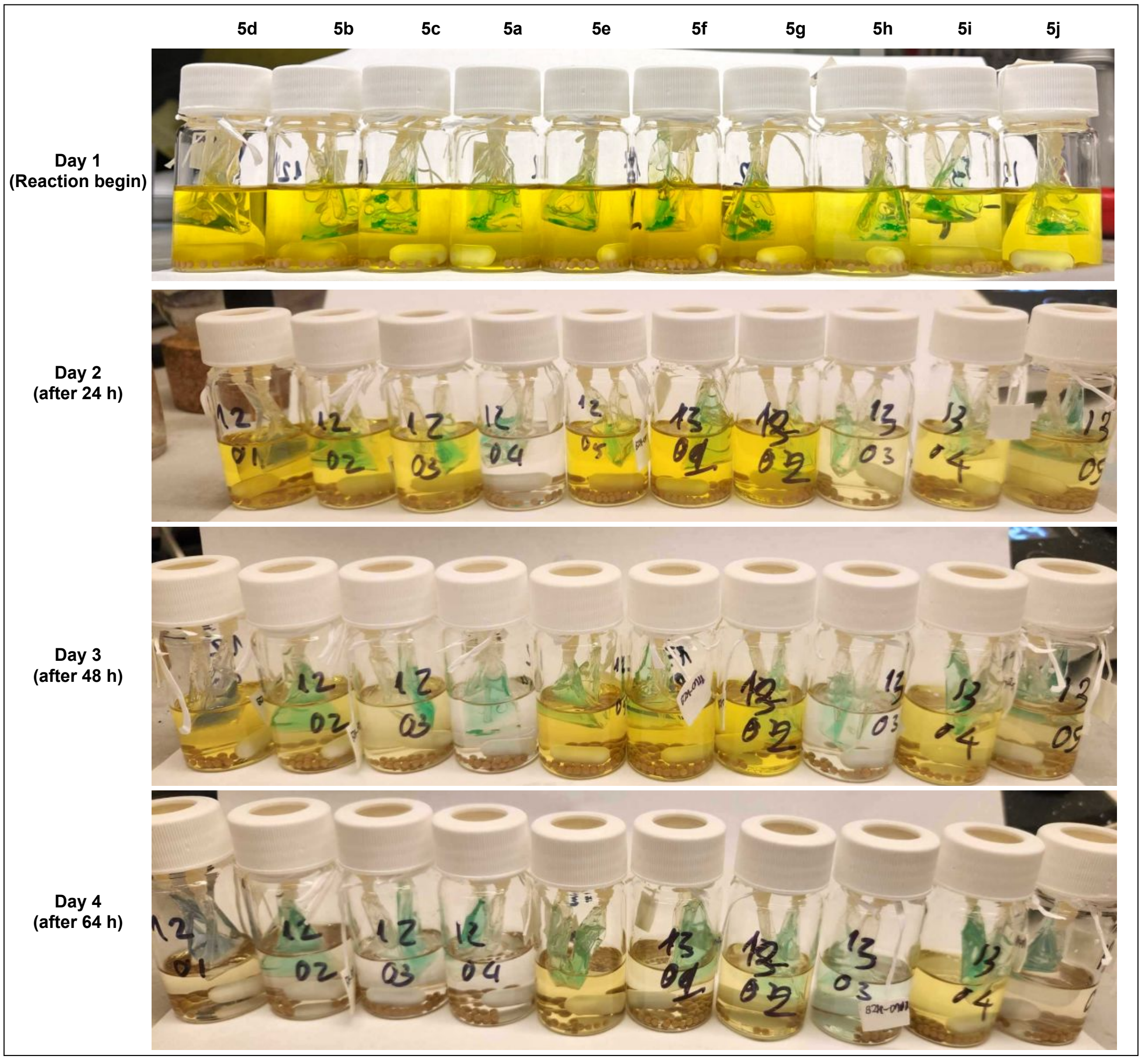

Figure S9. Reaction setups and monitoring of the disappearance of diazoacetate.

5. Recycle procedure with the Catalyst-in-Bag

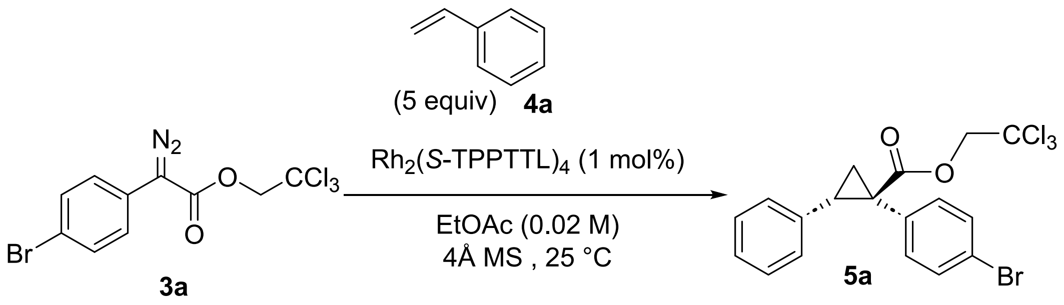

The first experiment was conducted according to general procedure A for ReactIR studies using styrene (5 equiv), 2,2,2-trichloroethyl 2-(4-bromophenyl)-2-diazoacetate (0.2 mmol), $Rh_2(S\text{-}TPPTTL)_4$ (1.0 mol%) and 4Å molecular sieve (100 wt%) in 10 mL of dried solvent (0.02 M) at room temperature. The bag separated from the reaction solution and went through a washing step in which it was soaked in a fresh EA solvent twice for 2 hours each to maximize the recovery of the product inside the bag. The bag was then subjected to another reaction mixture following general procedure A. The process was then repeated 3 more times to total 5 cycle experiments. (**Figure S10**) The experiments were conducted using ethyl acetate and DCM as solvents. *Note*: The reaction took ~1.5-2 days to reach full conversion of diazo.

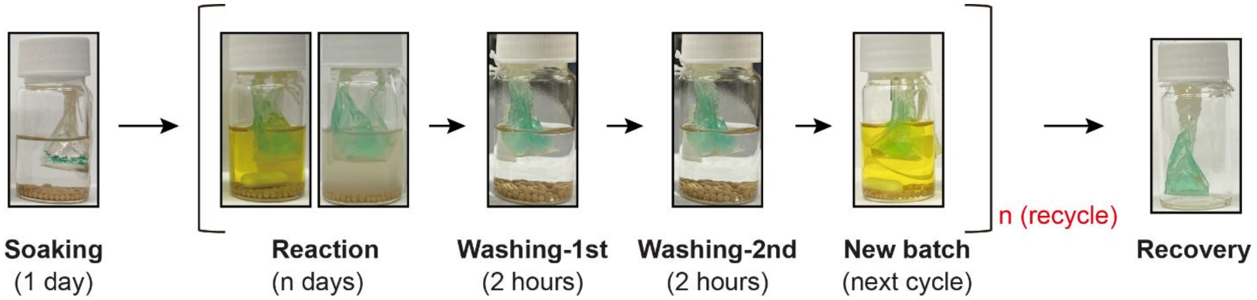

Figure S10. Schematic procedure for recycling experiments.

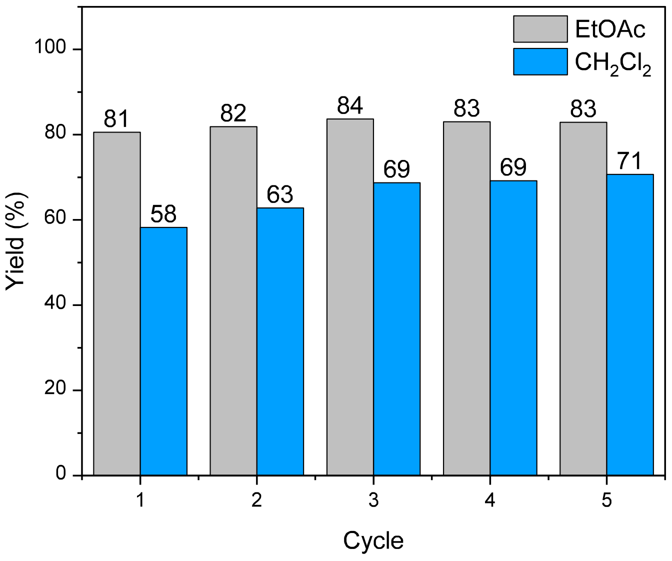

Figure S11. Recycling studies of the catalyst-in-bag system using EtOAc and CH_2Cl_2 as solvent with average reaction times of 2 days without twice washing steps. After the reaction was completed, the bag was immediately immersed in the next reaction solution. The yield (%) were calculated using H[1] NMR analysis with 1,3,5-Trimethoxybenzene as an internal standard.

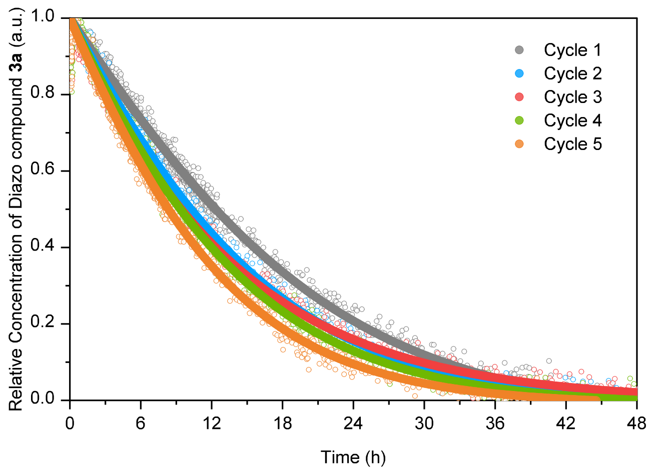

Figure S12. ReactIR study on recycling reactions. (The empty circles represent the raw data, while the solid circles display the Boltzmann fitting results, which are utilized in the main text.)

5.1. Ethyl acetate

5.1.1. EA – 1st cycle – 94.6%

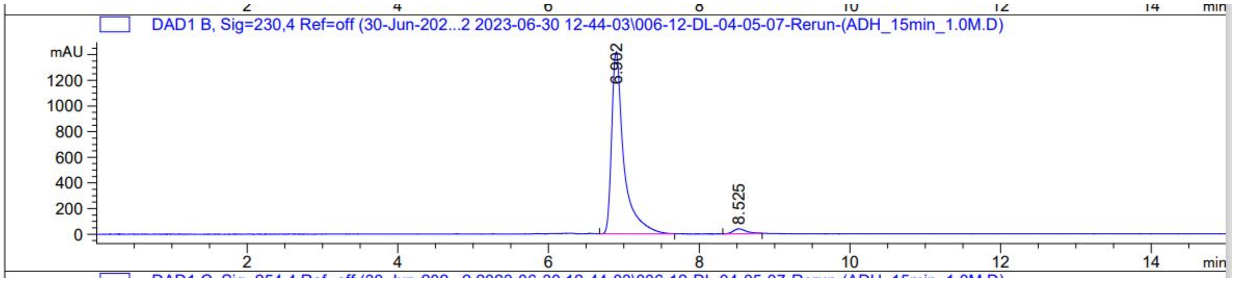

Signal 2: DAD1 B, Sig=230,4 Ref=off

Peak #	RetTime [min]	Type	Width [min]	Area [mAU*s]	Height [mAU]	Area %
1	6.902	VV R	0.1355	1.58940e4	1420.93176	97.2796
2	8.525	VV R	0.1395	444.47348	38.13410	2.7204
Totals :				1.63385e4	1459.06586	

5.1.2. EA – 2nd cycle – 93.8%

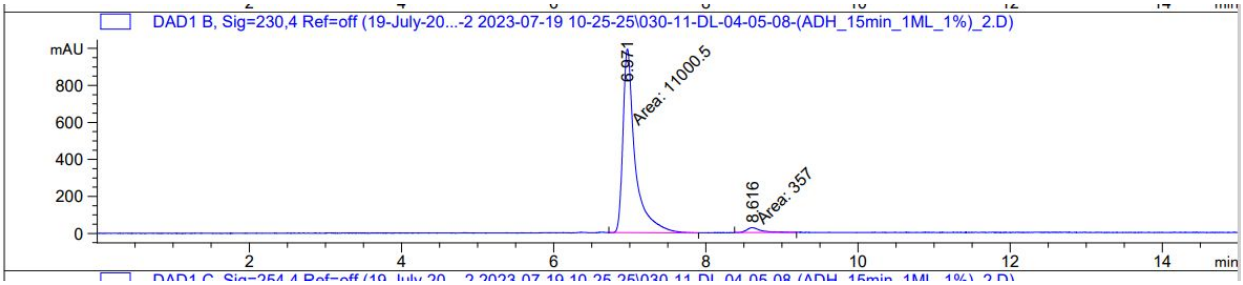

Signal 2: DAD1 B, Sig=230,4 Ref=off

Peak #	RetTime [min]	Type	Width [min]	Area [mAU*s]	Height [mAU]	Area %
1	6.971	MM	0.1849	1.10005e4	991.58142	96.8567
2	8.616	MM	0.2232	356.99960	26.66285	3.1433
Totals :				1.13575e4	1018.24427	

5.1.3. EA – 3rd cycle – 95.4% ee

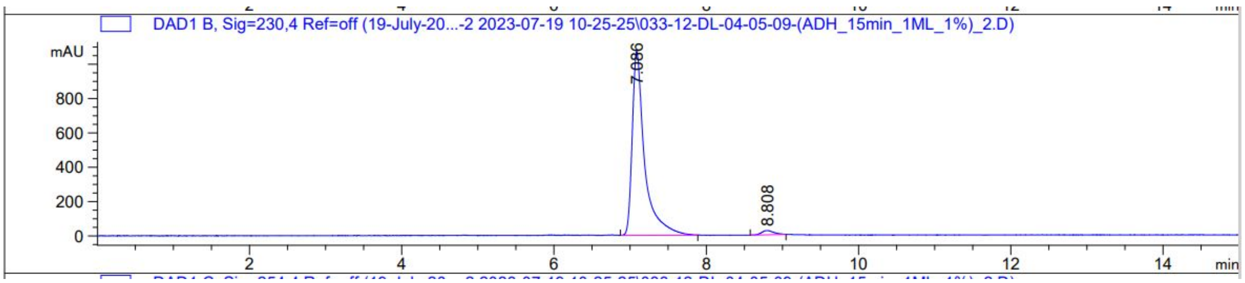

Signal 2: DAD1 B, Sig=230,4 Ref=off

Peak #	RetTime [min]	Type	Width [min]	Area [mAU*s]	Height [mAU]	Area %
1	7.086	VV R	0.1352	1.21008e4	1072.21667	97.7168
2	8.808	VV R	0.1354	282.73782	24.91269	2.2832

Totals : 1.23836e4 1097.12937

5.1.4. EA – 4th cycle – 93.4% ee

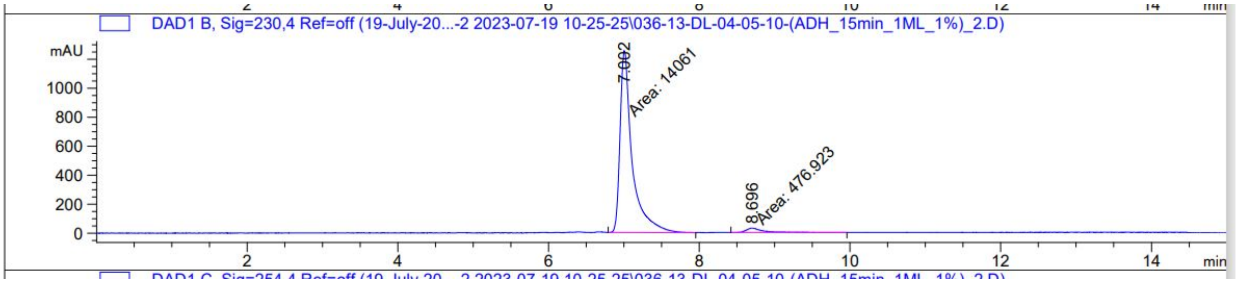

Signal 2: DAD1 B, Sig=230,4 Ref=off

Peak #	RetTime [min]	Type	Width [min]	Area [mAU*s]	Height [mAU]	Area %
1	7.002	MM	0.1866	1.40610e4	1255.92090	96.7195
2	8.696	MM	0.2635	476.92310	30.17029	3.2805

Totals : 1.45380e4 1286.09118

5.1.5. EA – 5th cycle – 93.4% ee

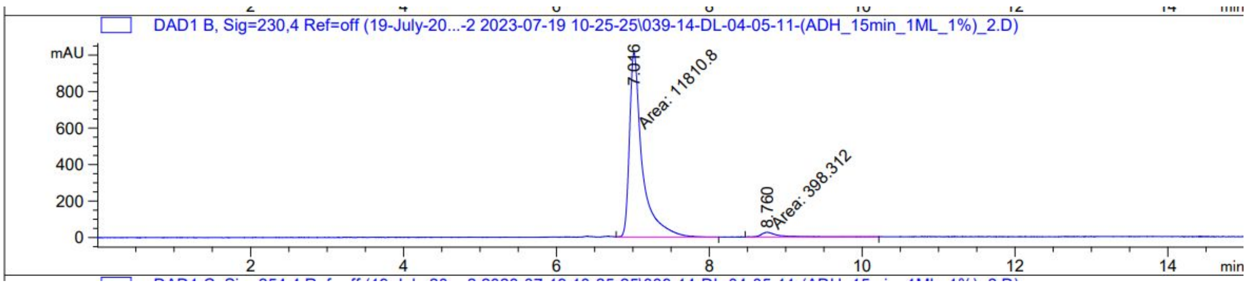


```
Signal 2: DAD1 B, Sig=230,4 Ref=off
```

Peak #	RetTime [min]	Type	Width [min]	Area [mAU*s]	Height [mAU]	Area %
1	7.016	MM	0.1947	1.18108e4	1010.90320	96.7376
2	8.760	MM	0.2604	398.31155	25.49481	3.2624
Totals :				1.22091e4	1036.39801	

5.2. Dichloromethane

5.2.1. DCM – 1st cycle – 83% ee

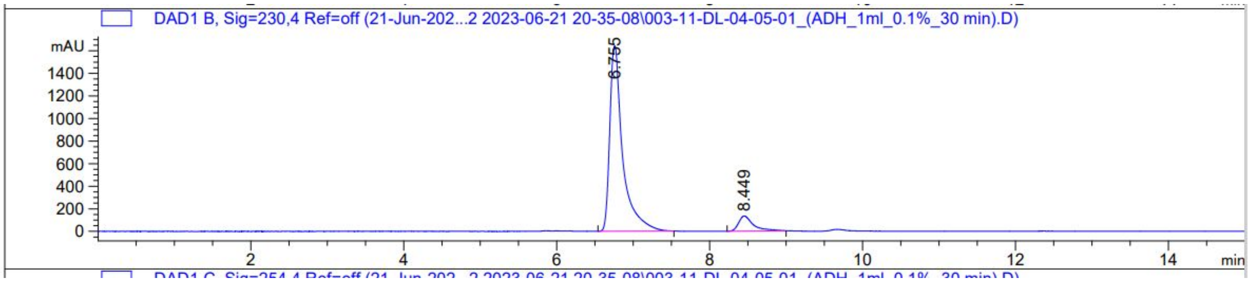


```
Signal 2: DAD1 B, Sig=230,4 Ref=off
```

Peak #	RetTime [min]	Type	Width [min]	Area [mAU*s]	Height [mAU]	Area %
1	6.755	BV R	0.1403	1.91781e4	1641.46851	91.4631
2	8.449	BV R	0.1612	1790.02808	133.78204	8.5369
Totals :				2.09681e4	1775.25055	

5.2.2. DCM – 2nd cycle – 85% ee

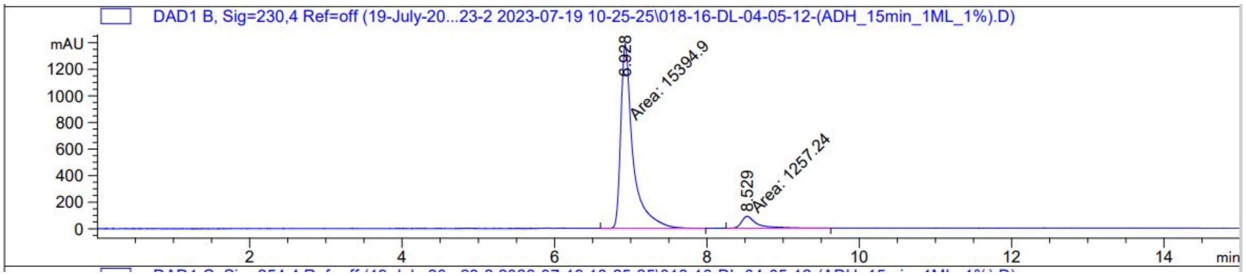

Signal 2: DAD1 B, Sig=230,4 Ref=off

Peak #	RetTime [min]	Type	Width [min]	Area [mAU*s]	Height [mAU]	Area %
1	6.928	MM	0.1846	1.53949e4	1390.29089	92.4500
2	8.529	MM	0.2328	1257.24402	90.01360	7.5500
Totals :				1.66521e4	1480.30449	

5.2.3. DCM – 3rd cycle – 84% ee

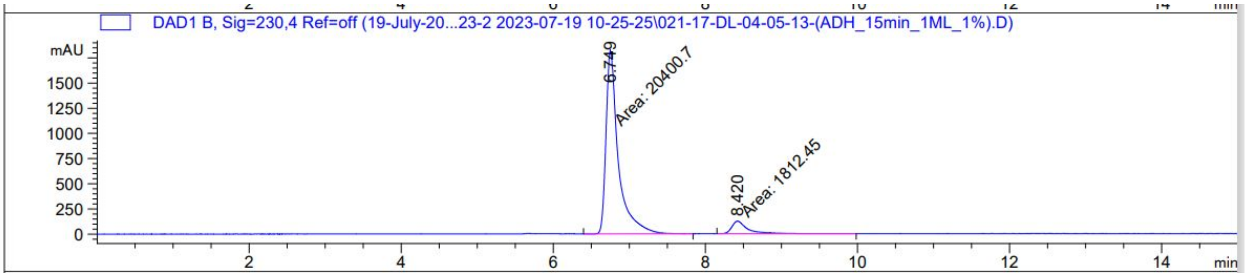

Signal 2: DAD1 B, Sig=230,4 Ref=off

Peak #	RetTime [min]	Type	Width [min]	Area [mAU*s]	Height [mAU]	Area %
1	6.749	MM	0.1861	2.04007e4	1827.24414	91.8406
2	8.420	MM	0.2409	1812.45435	125.38889	8.1594
Totals :				2.22132e4	1952.63303	

5.2.4. DCM – 4th cycle – 86% ee

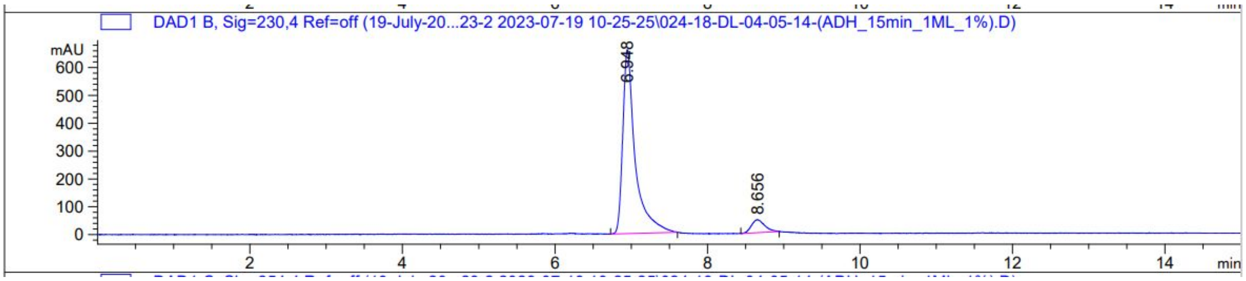

Signal 2: DAD1 B, Sig=230,4 Ref=off

Peak #	RetTime [min]	Type	Width [min]	Area [mAU*s]	Height [mAU]	Area %
1	6.948	VV R	0.1502	7489.91553	661.16919	92.9297
2	8.656	VV R	0.1454	569.85260	46.69811	7.0703
Totals :				8059.76813	707.86730	

5.2.5. DCM – 5th cycle – 84% ee

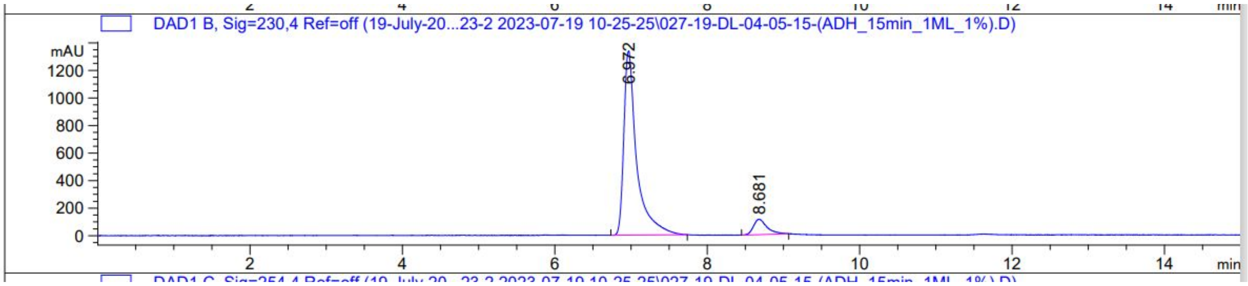

Signal 2: DAD1 B, Sig=230,4 Ref=off

Peak #	RetTime [min]	Type	Width [min]	Area [mAU*s]	Height [mAU]	Area %
1	6.972	VV R	0.1441	1.56421e4	1336.12366	91.9447
2	8.681	VV R	0.1472	1370.41492	110.90384	8.0553
Totals :				1.70125e4	1447.02750	

6. Scale-up Experiments

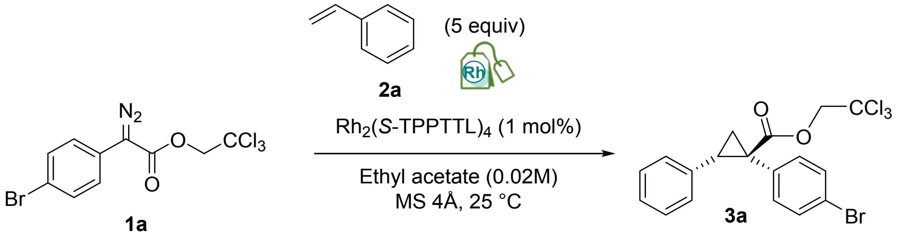

Before running a reaction, the dialysis membrane (10 cm × 30 mm) was folded and tightened on one side with a clip. Rh_2(*S*-TPTTL)$_4$ (37 mg, 0.005 mol%) was introduced into the membrane from the other, open side, which was then closed with another clip. After that, the above catalyst-in-bag was soaked in 60 mL of dry ethyl acetate for 1 day (**Figure S13a**). In an oven-dried 100 mL Pyrex round bottle equipped with a magnetic stir bar and 4Å activated molecular sieves (~1.0 g, 100 wt%), styrene (1.72 mL, 15.0 mmol, 5.0 equiv, filtered through silica plug to remove preservative) and 2,2-trichloroethyl 2-(4-bromophenyl)-2-diazoacetate (1.12 g, 3.00 mmol, 1.0 equiv.) were added. After vacuuming and back filling with N_2, 60 mL of dry ethyl acetate (distilled over CaH_2, stored with 4Å molecular sieves) was added (**Figure S13b**). The mixture was then stirred for 5 min, and subsequently the catalyst-in-bag from a separate, soaking vial containing Rh_2(*S*-TPPTTL)$_4$ (37.0 mg, 0.5 mol%) catalyst was introduced into the reaction vessel. The solution was then stirred overnight at room temperature. Upon reaction completion, while the solution was passed through a celite filter to remove molecular sieves, the catalyst-in-bag was immersed in another vial containing 60 mL ethyl acetate for 2 hours to extract the remaining reagents and products inside the bag (**Figure S13c**). At this stage, the catalyst-in-bag could either be used for another reaction or cut to recover the catalyst. The reaction mixture and washing mixture were then combined and concentrated under vacuum. The crude residue was purified by flash column chromatography (0-10% Et_2O in hexane) to obtain a white solid (1.25 g, 83 % yield, 92% ee). The

Rh_2(*S*-TPPTTL)$_4$-containing catalyst-in-bag was washed 4 more times to remove any remaining reagents, and the catalyst was recovered with 99% recovery with the purity of 92% (accessed by NMR, cyclopropane product was a major contamination). The catalyst could be further purified by flash chromatography (SiO_2, 0-20% ethyl acetate in hexane) to remove contaminated product to get >95% purity with 91% recovery (averaged of three runs).

Note: The reproducibility of recovery after purification was accessed by three parallel runs and the results for the first cycle were 88%, 92%, and 94%, respectively.

Note: Because of gas generation during the reaction, the headspace of the membrane must be adequate to accommodate the gas generation and the pressure build-up. The diffusion of gas outside the membrane is faster than the diffusion of substrates into the membrane but care should still be taken. Therefore, upon scaling up by increasing the concentrations and volume of the system, the reaction rate and related pressure buildup must be assessed and managed, otherwise the membrane may rupture.

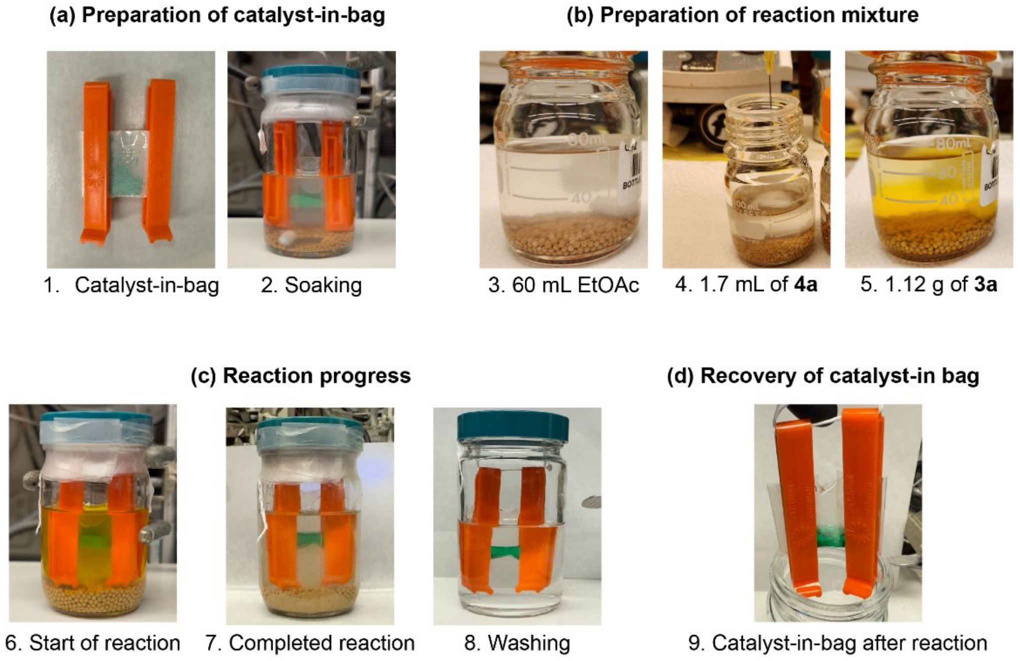

Figure S13. Illustration of scale-up experiments

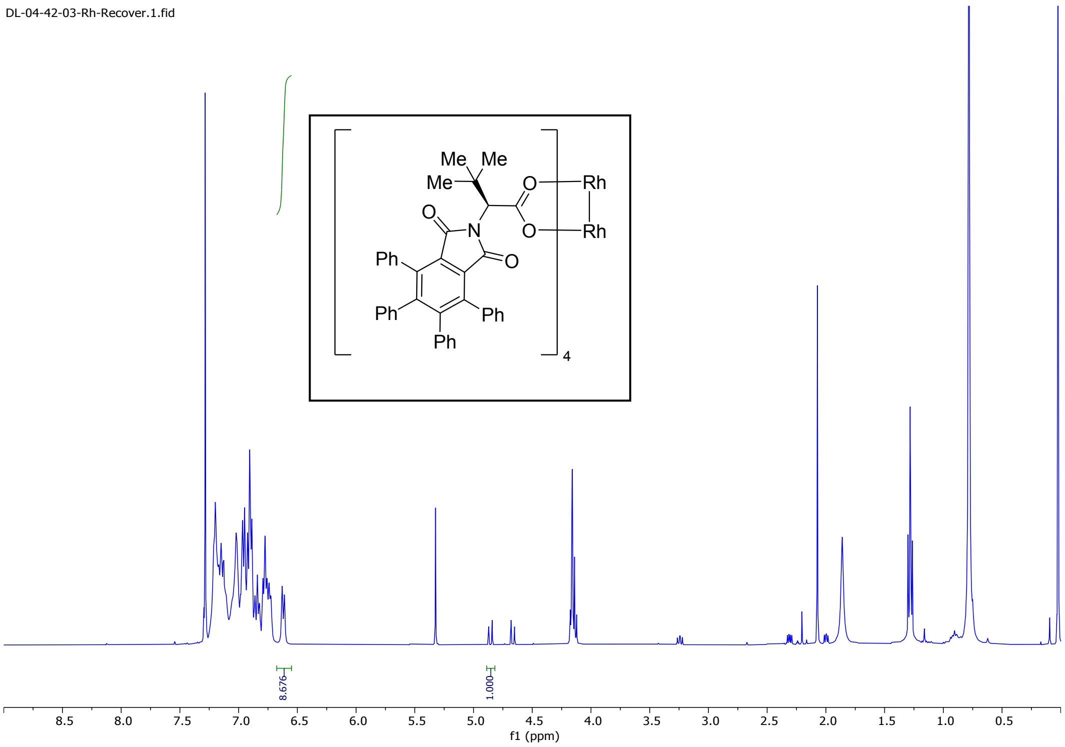

Figure S14. ^{1}H-NMR of recovered Rh_2(*S*-TPPTTL)$_4$ after washing 5 times.

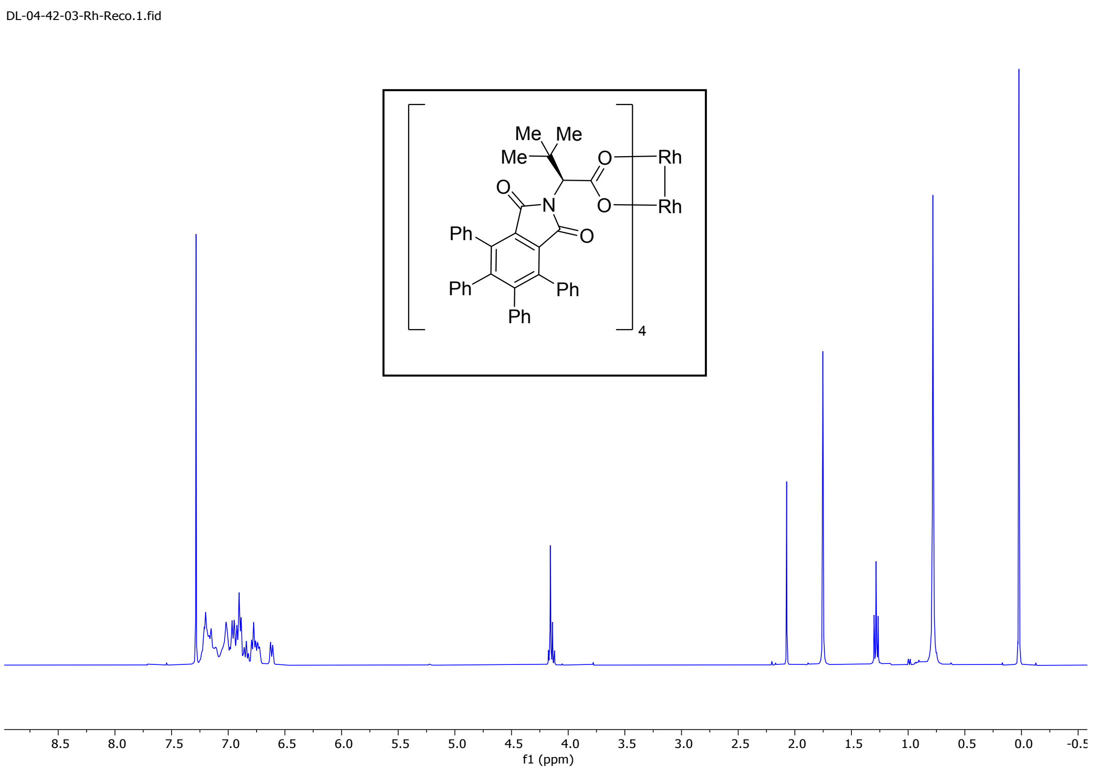

Figure S15. ^{1}H-NMR of recovered Rh_2(S-TPPTTL)$_4$ after washing 5 times and flash chromatography

7. Preparation of substrates

Diazo compounds used in this study were prepared according to reported procedures:[1, 2]

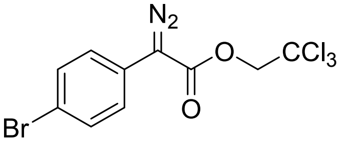

3a

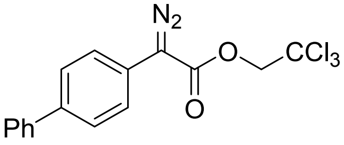

3f

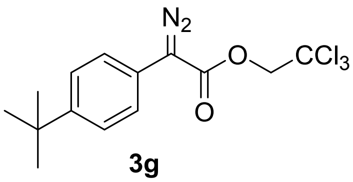

3g

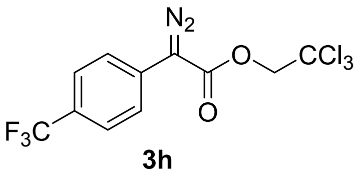

3h

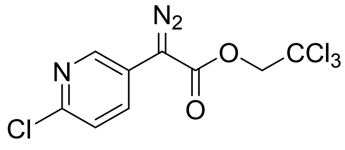

3j

8. Characterization of cyclopropanation products

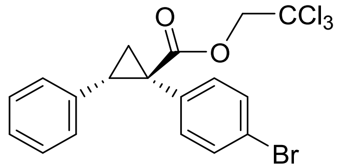

5a

2,2,2-trichloroethyl (1R,2S)-1-(4-bromophenyl)-2-phenylcyclopropane-1-carboxylate (3a)

Prepared according to general procedure A for bench-scale cyclopropanation. Styrene (115 μL, 1.0 mmol, 5.0 equiv), 2,2,2-trichloroethyl 2-(4- bromophenyl)-2-diazoacetate (74.5 mg, 0.2 mmol, 1.0 equiv), and Rh_2(*S*-TPPTTL)$_4$ (5.0 mg, 1 mol%, in benzoylated cellulose membrane) were used. Purification by flash column chromatography (0% hexanes/diethyl ether, 0-10% hexanes/diethyl ether) afforded **3a** as a crystalline solid (67.2 mg, 75%, 93% ee). Spectra matched the literature precedent.[1]

^{1}H NMR (400 MHz, $CDCl_3$) δ 7.33 – 7.24 (m, 2H), 7.18 – 7.10 (m, 3H), 7.01 – 6.92 (m, 2H), 6.91 – 6.78 (m, 2H), 4.86 (d, *J* = 11.9 Hz, 1H), 4.67 (d, *J* = 11.9 Hz, 1H), 3.25 (dd, *J* = 9.4, 7.4 Hz, 1H), 2.31 (dd, *J* = 9.4, 5.2 Hz, 1H), 2.00 (dd, *J* = 7.5, 5.2 Hz, 1H).

HPLC: The enantiopurity was determined to be 93% ee by chiral HPLC analysis (Chiracel AD-H, 1.0% IPA/Hexanes, 1.0 mL/min, λ=230 nm, retention time of 6.97 min (major) and 8.62 min (minor).

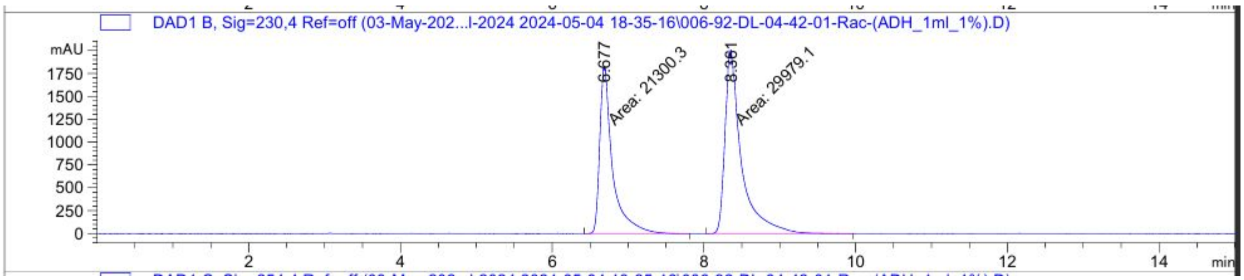

Signal 2: DAD1 B, Sig=230,4 Ref=off

Peak #	RetTime [min]	Type	Width [min]	Area [mAU*s]	Height [mAU]	Area %
1	6.677	MM	0.1958	2.13003e4	1813.00134	41.5377
2	8.361	MM	0.2487	2.99791e4	2009.23865	58.4623
Totals :				5.12793e4	3822.23999	

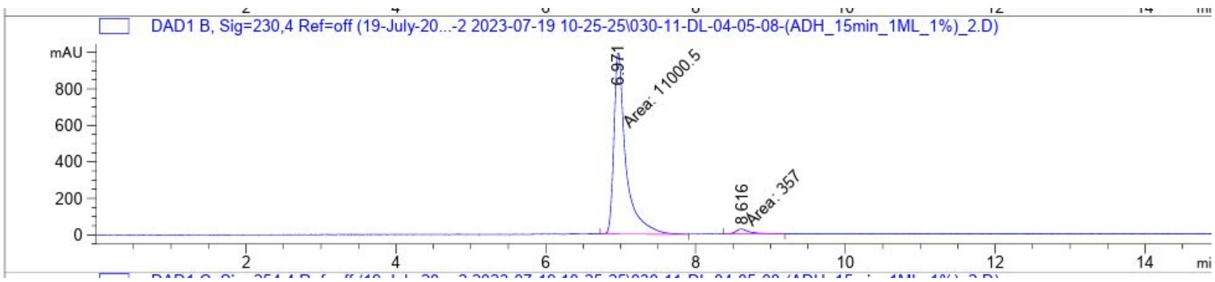

Signal 2: DAD1 B, Sig=230,4 Ref=off

Peak #	RetTime [min]	Type	Width [min]	Area [mAU*s]	Height [mAU]	Area %
1	6.971	MM	0.1849	1.10005e4	991.58142	96.8567
2	8.616	MM	0.2232	356.99960	26.66285	3.1433
Totals :				1.13575e4	1018.24427	

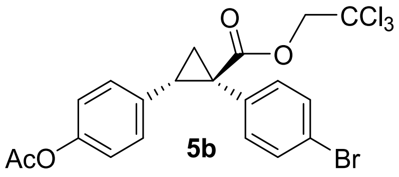

2,2,2-trichloroethyl (1R,2S)-2-(4-acetoxyphenyl)-1-(4-bromophenyl)cyclopropane-1-carboxylate (3b)

Prepared according to general procedure A for bench-scale cyclopropanation. 4-vinylphenyl acetate (162 mg, 1.0 mmol, 5.0 equiv), 2,2,2-trichloroethyl 2-(4- bromophenyl)-2-diazoacetate (74.5 mg, 0.2 mmol, 1.0 equiv), and Rh_2(*S*-TPPTTL)$_4$ (5.0 mg, 1 mol%, in benzoylated cellulose membrane) were used. Purification by flash column chromatography (0% hexanes/diethyl ether,

0-10% hexanes/diethyl ether) using afforded **3b** as a crystalline solid (83.1 mg, 82%, 96% ee). Spectra matched the literature precedent.[1]

^{1}H NMR (400 MHz, $CDCl_3$) δ 7.28 (d, J = 8.6 Hz, 2H), 6.94 (d, J = 8.5 Hz, 2H), 6.85 (d, J = 8.7 Hz, 2H), 6.79 (d, J = 8.7 Hz, 2H), 4.83 (d, J = 11.9 Hz, 1H), 4.63 (d, J = 11.9 Hz, 1H), 3.20 (dd, J = 9.4, 7.4 Hz, 1H), 2.29 (dd, J = 9.4, 5.3 Hz, 1H), 2.25 (s, 3H), 1.92 (dd, J = 7.5, 5.3 Hz, 1H).

HPLC: The enantiopurity was determined to be 96% ee by chiral HPLC analysis (Chiracel AD-H, 1.0% IPA/Hexanes, 1.0 mL/min, λ=230 nm, retention time of 16.38 min (major) and 31.45 min (minor).

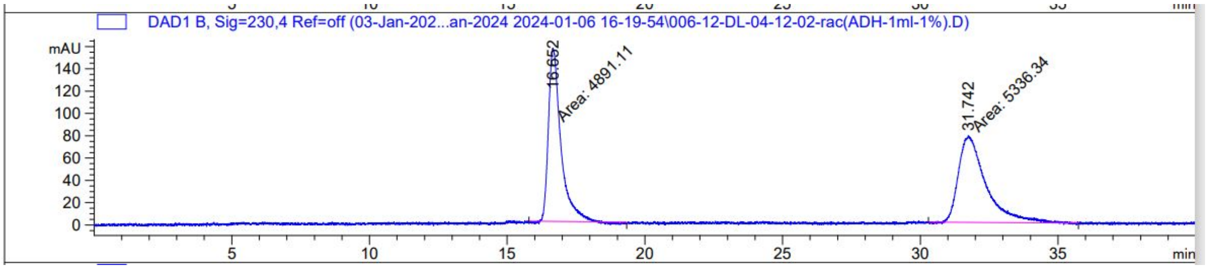

Signal 2: DAD1 B, Sig=230,4 Ref=off

Peak #	RetTime [min]	Type	Width [min]	Area [mAU*s]	Height [mAU]	Area %
1	16.652	MM	0.5252	4891.11182	155.21062	47.8234
2	31.742	MM	1.1434	5336.33691	77.78252	52.1766
Totals :				1.02274e4	232.99313	

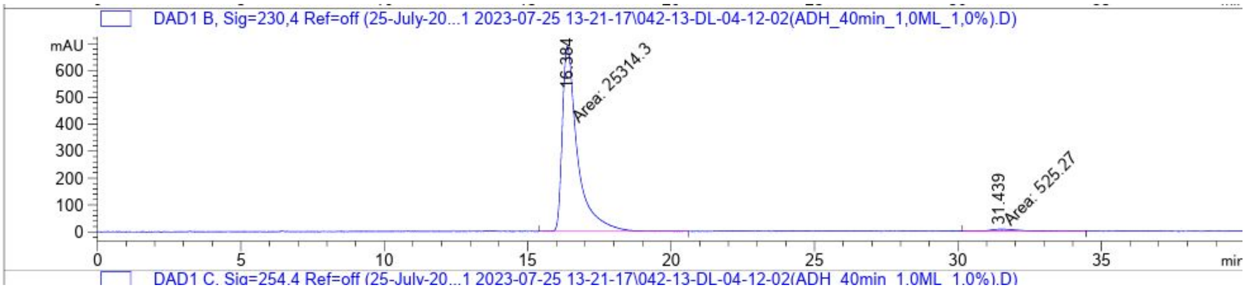

Signal 2: DAD1 B, Sig=230,4 Ref=off

Peak #	RetTime [min]	Type	Width [min]	Area [mAU*s]	Height [mAU]	Area %
1	16.384	MM	0.6132	2.53143e4	688.06653	97.9672
2	31.439	MM	1.0571	525.26996	8.28154	2.0328
Totals :				2.58395e4	696.34807	

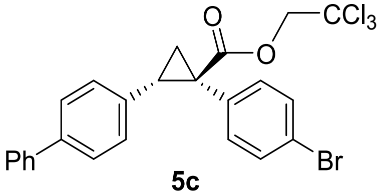

5c

2,2,2-trichloroethyl (1R,2S)-2-([1,1'-biphenyl]-4-yl)-1-(4-bromophenyl)cyclopropane-1-carboxylate (3c)

Prepared according to general procedure A for bench-scale cyclopropanation. 4-vinyl-1,1'-biphenyl (162 mg, 1.0 mmol, 5.0 equiv), 2,2,2-trichloroethyl 2-(4- bromophenyl)-2-diazoacetate (74.5 mg, 0.2 mmol, 1.0 equiv), and Rh_2(*S*-TPPTTL)$_4$ (5.0 mg, 1 mol%, in benzoylated cellulose membrane) were used. Purification by flash column chromatography (0% hexanes/diethyl ether, 0-10% hexanes/diethyl ether) afforded **3c** as a crystalline solid (71.5 mg, 68%, 97% ee). Spectra matched the literature precedent.[1]

^{1}H NMR (400 MHz, $CDCl_3$) δ δ 7.56 – 7.49 (m, 2H), 7.44 – 7.26 (m, 7H), 6.98 (d, *J* = 8.5 Hz, 2H), 6.86 (d, *J* = 8.2 Hz, 2H), 4.84 (d, *J* = 11.9 Hz, 1H), 4.65 (d, *J* = 11.9 Hz, 1H), 3.25 (dd, *J* = 9.4, 7.4 Hz, 1H), 2.32 (dd, *J* = 9.4, 5.2 Hz, 1H), 1.99 (dd, *J* = 7.5, 5.2 Hz, 1H).

HPLC: The enantiopurity was determined to be 97% ee by chiral HPLC analysis (Chiracel AD-H, 1.0% IPA/Hexanes, 1.0 mL/min, λ=230 nm, retention time of 9.81 min (major) and 14.8 min (minor).

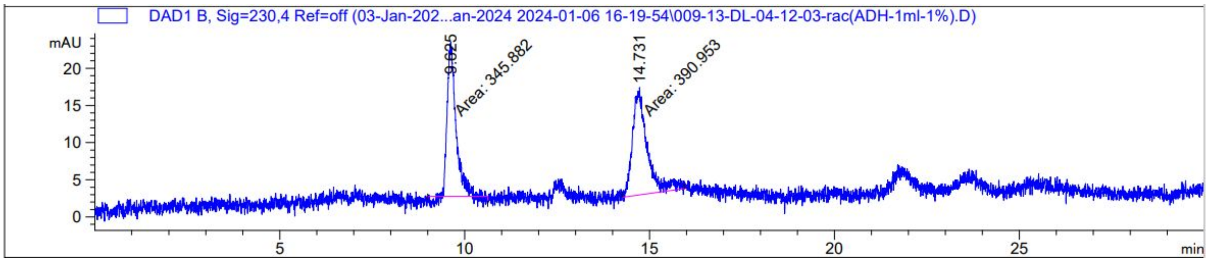


```
Signal 2: DAD1 B, Sig=230,4 Ref=off

Peak RetTime Type  Width     Area       Height      Area
  #   [min]        [min]   [mAU*s]      [mAU]        %
----|-------|----|-------|----------|----------|--------|
   1   9.625 MM    0.2783  345.88153   20.71578  46.9415
   2  14.731 MM    0.4497  390.95303   14.48946  53.0585

Totals :                   736.83456   35.20525
```

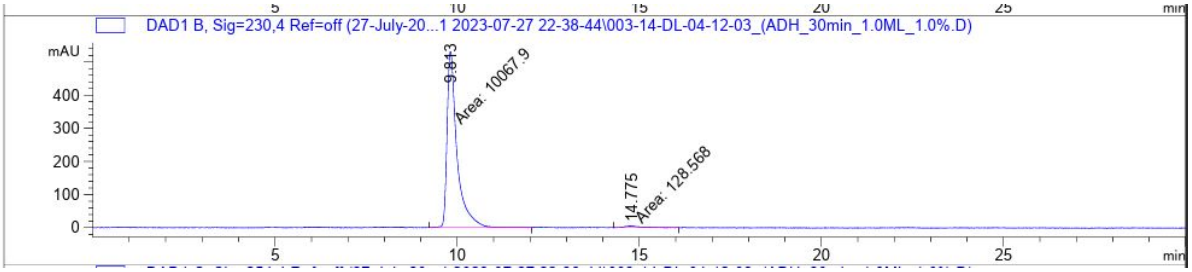


```
Signal 2: DAD1 B, Sig=230,4 Ref=off

Peak RetTime Type  Width     Area       Height      Area
  #   [min]        [min]   [mAU*s]      [mAU]        %
----|-------|----|-------|----------|----------|--------|
   1   9.813 MM    0.3160 1.00679e4  531.07440  98.7391
   2  14.775 MM    0.4065  128.56764    5.27185   1.2609

Totals :                  1.01964e4  536.34625
```

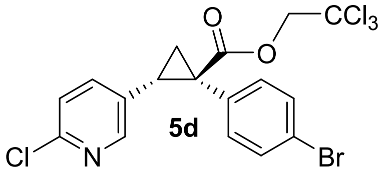

2,2,2-trichloroethyl (1R,2S)-1-(4-bromophenyl)-2-(6-chloropyridin-3-yl)cyclopropane-1-carboxylate (3d)

Prepared according to general procedure A for bench-scale cyclopropanation. 2-chloro-5-vinylpyridine (140 mg, 1.0 mmol, 5.0 equiv), 2,2,2-trichloroethyl 2-(4- bromophenyl)-2-diazoacetate (74.5 mg, 0.2 mmol, 1.0 equiv), and Rh_2(*S*-TPPTTL)$_4$ (5.0 mg, 1 mol%, in benzoylated cellulose membrane) were used. Purification by flash column chromatography (0% hexanes/diethyl ether, 0-10% hexanes/diethyl ether) afforded **3d** as a clear oil (56.3 mg, 58%, 95% ee). Spectra matched the literature precedent.[3]

^{1}H NMR (400 MHz, $CDCl_3$) δ 8.05 (d, *J* = 2.5 Hz, 1H), 7.33 (d, *J* = 8.5 Hz, 2H), 7.03 (d, *J* = 8.3 Hz, 1H), 6.95 (d, *J* = 8.4 Hz, 2H), 6.83 (dd, *J* = 8.3, 2.6 Hz, 1H), 4.84 (d, *J* = 11.9 Hz, 1H), 4.64 (d, *J* = 11.9 Hz, 1H), 3.18 (dd, *J* = 9.4, 7.3 Hz, 1H), 2.34 (dd, *J* = 9.4, 5.4 Hz, 1H), 1.93 (dd, *J* = 7.3, 5.4 Hz, 1H).

HPLC: The enantiopurity was determined to be 95% ee by chiral HPLC analysis (Chiracel OD-H, 1.0% IPA/Hexanes, 1.0 mL/min, λ=230 nm, retention time of 28.54 min (minor) and 35.65 min (major).

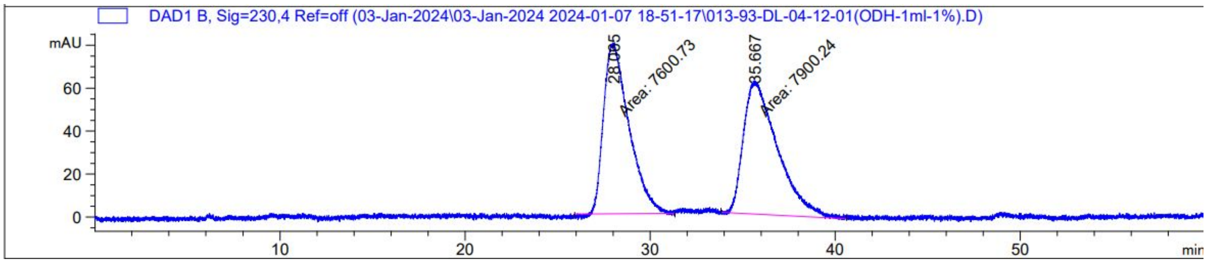

Signal 2: DAD1 B, Sig=230,4 Ref=off

Peak #	RetTime [min]	Type	Width [min]	Area [mAU*s]	Height [mAU]	Area %
1	28.065	MM	1.5878	7600.73438	79.78114	49.0339
2	35.667	MM	2.1232	7900.23975	62.01577	50.9661
Totals :				1.55010e4	141.79691	

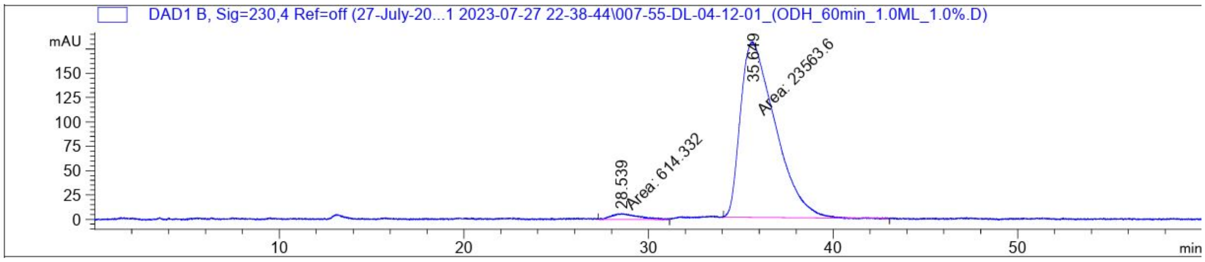

Peak #	RetTime [min]	Type	Width [min]	Area [mAU*s]	Height [mAU]	Area %
1	28.539	MM	1.6398	614.33209	6.24384	2.5409
2	35.649	MM	2.1731	2.35636e4	180.72054	97.4591
Totals :				2.41780e4	186.96438	

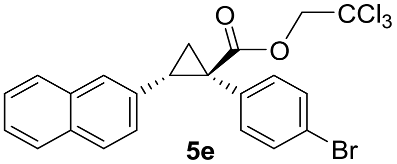

5e

2,2,2-trichloroethyl (1R,2S)-1-(4-bromophenyl)-2-(naphthalen-2-yl)cyclopropane-1-carboxylate (3e)

Prepared according to general procedure A for bench-scale cyclopropanation. 2-vinylnaphthalene (154 mg, 1.0 mmol, 5.0 equiv), 2,2,2-trichloroethyl 2-(4- bromophenyl)-2-diazoacetate (0.2 mmol, 74.5 mg, 1.0 equiv), and Rh_2(*S*-TPPTTL)$_4$ (5.0 mg, 1 mol%, in benzoylated cellulose membrane) were used. Purification by flash column chromatography (0% hexanes/diethyl ether, 0-10% hexanes/diethyl ether) afforded **3e** as a crystalline (64.6 mg, 65%, 95% ee). Spectra matched the literature precedent.[4]

^{1}H NMR (400 MHz, CDCl$_3$) δ 7.75 – 7.70 (m, 1H), 7.68 – 7.62 (m, 1H), 7.57 (d, *J* = 8.5 Hz, 1H), 7.45 – 7.38 (m, 2H), 7.38 – 7.35 (m, 1H), 7.22 (d, *J* = 8.4 Hz, 2H), 6.98 (d, *J* = 8.4 Hz, 2H),

6.84 (dd, J = 8.5, 1.8 Hz, 1H), 4.85 (d, J = 11.9 Hz, 1H), 4.67 (d, J = 11.9 Hz, 1H), 3.39 (dd, J = 9.4, 7.4 Hz, 1H), 2.37 (dd, J = 9.3, 5.2 Hz, 1H), 2.11 (dd, J = 7.5, 5.2 Hz, 1H).

HPLC: The enantiopurity was determined to be 95% ee by chiral HPLC analysis (Chiracel OD-H, 1.0% IPA/Hexanes, 1.0 mL/min, λ=230 nm, retention time of 13.90 min (minor) and 15.97 min (major).

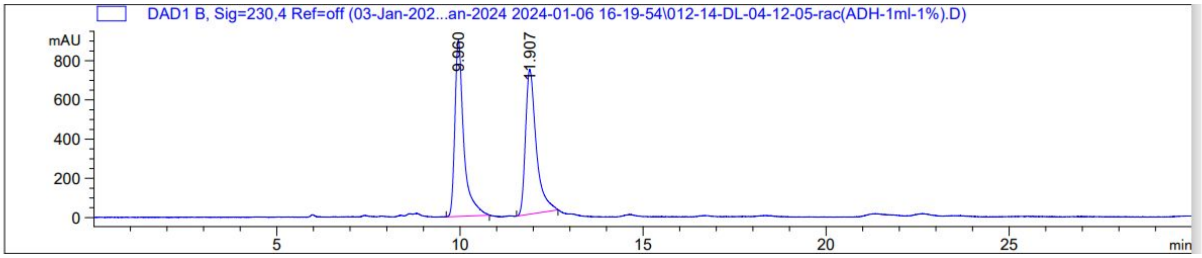

Signal 2: DAD1 B, Sig=230,4 Ref=off

Peak #	RetTime [min]	Type	Width [min]	Area [mAU*s]	Height [mAU]	Area %
1	9.960	BV R	0.1982	1.51306e4	899.53882	50.1674
2	11.907	BV R	0.2383	1.50297e4	740.37073	49.8326
Totals :				3.01603e4	1639.90955	

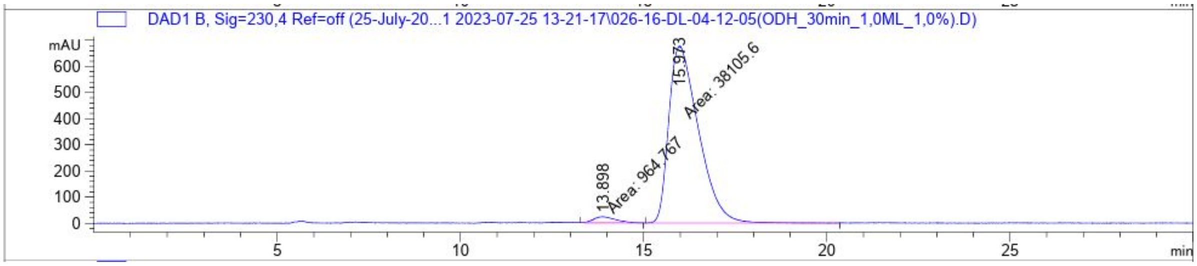

Signal 2: DAD1 B, Sig=230,4 Ref=off

Peak #	RetTime [min]	Type	Width [min]	Area [mAU*s]	Height [mAU]	Area %
1	13.898	MM	0.6781	964.76678	23.71084	2.4693
2	15.973	MM	0.9353	3.81056e4	679.01514	97.5307
Totals :				3.90703e4	702.72598	

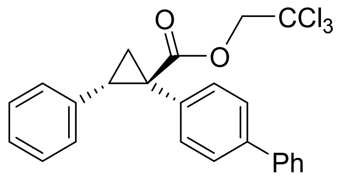

5f

2,2,2-trichloroethyl (1R,2S)-1-([1,1'-biphenyl]-4-yl)-2-phenylcyclopropane-1-carboxylate (3f)

Prepared according to general procedure A for bench-scale cyclopropanation. Styrene (115 μL, 1.0 mmol, 5.0 equiv), 2,2,2-trichloroethyl 2-([1,1'-biphenyl]-4-yl)-2-diazoacetate (73.9 mg, 0.200 mmol, 1.0 equiv), and Rh_2(*S*-TPPTTL)$_4$ (5.0 mg, 1 mol%, in benzoylated cellulose membrane) were used. Purification by flash column chromatography (0% hexanes/diethyl ether, 0-10% hexanes/diethyl ether) afforded **3f** as a crystalline solid (68.1 mg, 76%, 83% ee). Spectra matched the literature precedent.[4]

^{1}H NMR (400 MHz, $CDCl_3$) δ 7.55 – 7.48 (m, 2H), 7.43 – 7.36 (m, 4H), 7.33 – 7.28 (m, 1H), 7.13 (d, *J* = 8.3 Hz, 2H), 7.08 (dd, *J* = 5.1, 1.9 Hz, 3H), 6.88 – 6.81 (m, 2H), 4.86 (d, *J* = 11.9 Hz, 1H), 4.67 (d, *J* = 11.9 Hz, 1H), 3.24 (dd, *J* = 9.4, 7.4 Hz, 1H), 2.32 (dd, *J* = 9.4, 5.1 Hz, 1H), 2.04 (dd, *J* = 7.4, 5.1 Hz, 1H).

HPLC: The enantiopurity was determined to be 83% ee by chiral HPLC analysis (Chiracel AD-H, 1.0% IPA/Hexanes, 1.0 mL/min, λ=230 nm, retention time of 8.48 min (major) and 9.62 min (minor).

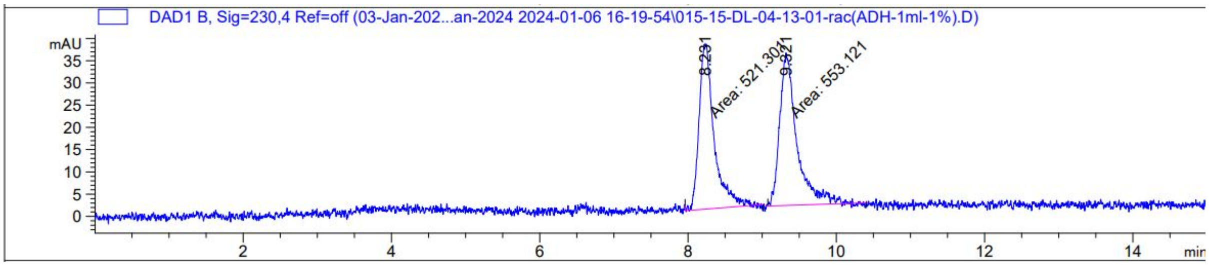

Signal 2: DAD1 B, Sig=230,4 Ref=off

Peak #	RetTime [min]	Type	Width [min]	Area [mAU*s]	Height [mAU]	Area %
1	8.231	MM	0.2334	521.30139	37.22338	48.5192
2	9.321	MM	0.2683	553.12146	34.36429	51.4808
Totals :				1074.42285	71.58767	

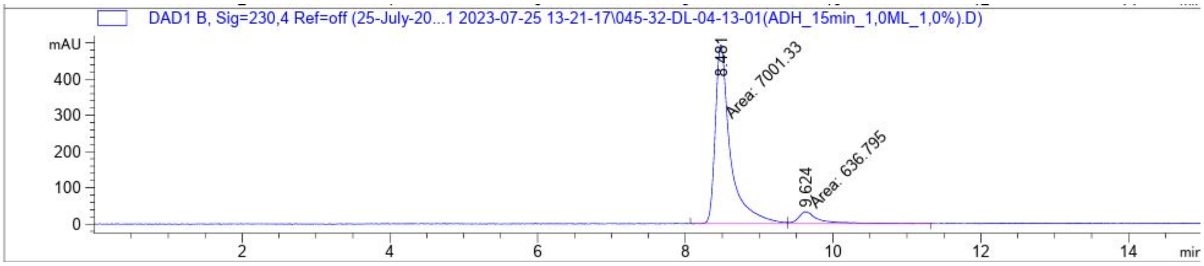

Signal 2: DAD1 B, Sig=230,4 Ref=off

Peak #	RetTime [min]	Type	Width [min]	Area [mAU*s]	Height [mAU]	Area %
1	8.481	MF	0.2364	7001.33252	493.71082	91.6629
2	9.624	FM	0.3200	636.79468	33.16869	8.3371
Totals :				7638.12720	526.87951	

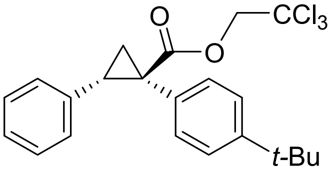

5g

2,2,2-trichloroethyl (1R,2S)-1-(4-(tert-butyl)phenyl)-2-phenylcyclopropane-1-carboxylate (3g)

Prepared according to general procedure A for bench-scale cyclopropanation. Styrene (115 µL, 1.0 mmol, 5.0 equiv), 2,2,2-trichloroethyl 2-(4-(tert-butyl)phenyl)-2-diazoacetate (69.9 mg, 0.200 mmol, 1.0 equiv), and Rh_2(*S*-TPPTTL)$_4$ (5.0 mg, 1 mol%, in benzoylated cellulose membrane) were used. Purification by flash column chromatography (0% hexanes/diethyl ether, 0-10% hexanes/diethyl ether) afforded **3g** as a crystalline solid (53.3 mg, 63%, 76% ee). Spectra matched the literature precedent.[1]

^{1}H NMR (400 MHz, $CDCl_3$) δ 7.13 (d, J = 8.4 Hz, 2H), 7.09 – 7.02 (m, 3H), 6.97 (d, J = 8.4 Hz, 2H), 6.82 – 6.74 (m, 2H), 4.83 (d, J = 11.9 Hz, 1H), 4.65 (d, J = 11.9 Hz, 1H), 3.18 (dd, J = 9.4, 7.4 Hz, 1H), 2.28 (dd, J = 9.4, 5.1 Hz, 1H), 1.97 (dd, J = 7.4, 5.1 Hz, 1H), 1.22 (s, 9H).

HPLC: The enantiopurity was determined to be 76% ee by chiral HPLC analysis (RRWhelk, 1.0% IPA/Hexanes, 1.0 mL/min, λ=230 nm, retention time of 5.57 min (major) and 7.11 min (minor).

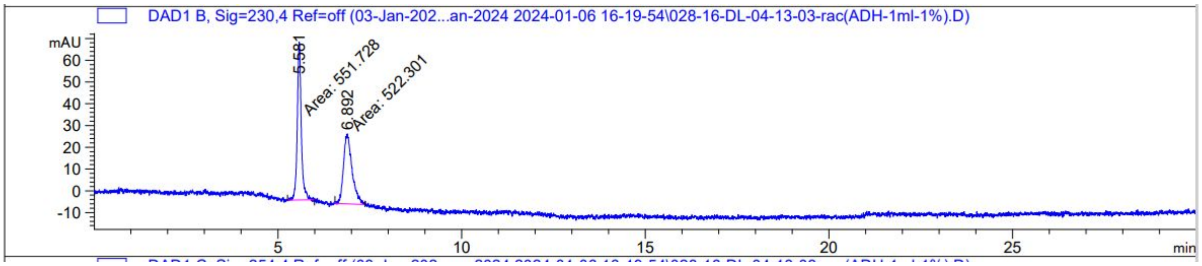

Signal 2: DAD1 B, Sig=230,4 Ref=off

Peak #	RetTime [min]	Type	Width [min]	Area [mAU*s]	Height [mAU]	Area %
1	5.581	MM	0.1268	551.72766	72.50870	51.3699
2	6.892	MM	0.2702	522.30115	32.22279	48.6301
Totals :				1074.02881	104.73149	

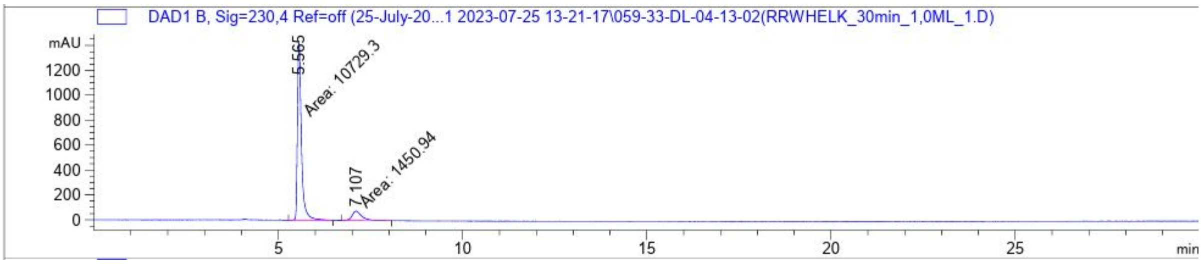

Signal 2: DAD1 B, Sig=230,4 Ref=off

Peak #	RetTime [min]	Type	Width [min]	Area [mAU*s]	Height [mAU]	Area %
1	5.565	MM	0.1258	1.07293e4	1421.48950	88.0878
2	7.107	MM	0.3171	1450.93896	76.25286	11.9122
Totals :				1.21802e4	1497.74236	

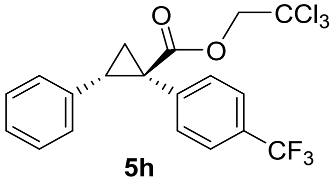

5h

2,2,2-trichloroethyl (1R,2S)-2-phenyl-1-(4-(trifluoromethyl)phenyl)cyclopropane-1-carboxylate (3h)

Prepared according to general procedure A for bench-scale cyclopropanation. Styrene (115 µL, 1.0 mmol, 5.0 equiv), 2,2,2-trichloroethyl 2-diazo-2-(4-(trifluoromethyl)phenyl)acetate (73.2 mg, 0.200 mmol, 1.0 equiv), and Rh_2(*S*-TPPTTL)$_4$ (5.0 mg, 1 mol%, in benzoylated cellulose membrane) were used. Purification by flash column chromatography (0% hexanes/diethyl ether, 0-10% hexanes/diethyl ether) afforded **3h** as a crystalline solid (62.8 mg, 72%, 89% ee). Spectra matched the literature precedent.[4]

^{1}H NMR (400 MHz, CDCl$_3$) δ 7.43 – 7.36 (m, 2H), 7.22 – 7.16 (m, 2H), 7.13 – 7.07 (m, 3H), 6.83 – 6.76 (m, 2H), 4.83 (d, *J* = 11.9 Hz, 1H), 4.65 (d, *J* = 11.9 Hz, 1H), 3.27 (dd, *J* = 9.4, 7.5 Hz, 1H), 2.33 (dd, *J* = 9.4, 5.3 Hz, 1H), 2.04 (dd, *J* = 7.5, 5.3 Hz, 1H).

HPLC: The enantiopurity was determined to be 89% ee by chiral HPLC analysis (Chiracel AD-H, 1.0% IPA/Hexanes, 1.0 mL/min, λ=230 nm, retention time of 5.83 min (major) and 7.46 min (minor).

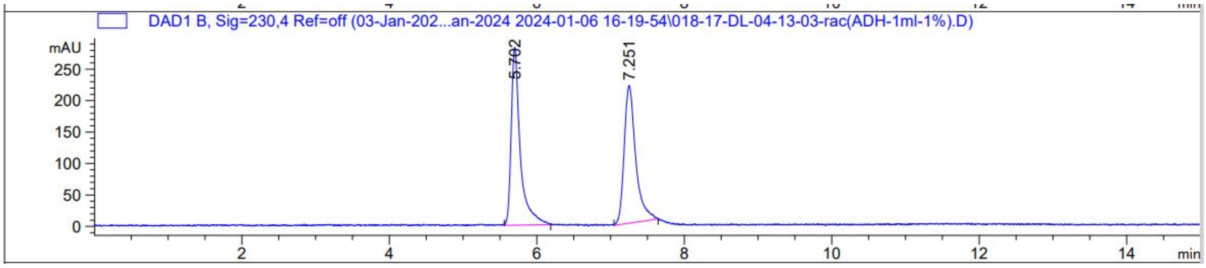

Signal 2: DAD1 B, Sig=230,4 Ref=off

Peak #	RetTime [min]	Type	Width [min]	Area [mAU*s]	Height [mAU]	Area %
1	5.702	BV R	0.1175	2405.47144	282.60422	50.3857
2	7.251	BV R	0.1284	2368.64771	219.16422	49.6143
Totals :				4774.11914	501.76843	

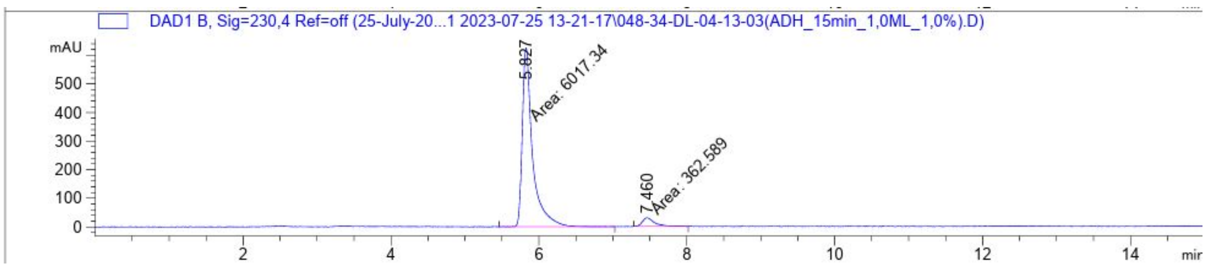

Signal 2: DAD1 B, Sig=230,4 Ref=off

Peak #	RetTime [min]	Type	Width [min]	Area [mAU*s]	Height [mAU]	Area %
1	5.827	MM	0.1604	6017.34473	625.28473	94.3167
2	7.460	MM	0.1990	362.58926	30.36497	5.6833
Totals :				6379.93399	655.64970	

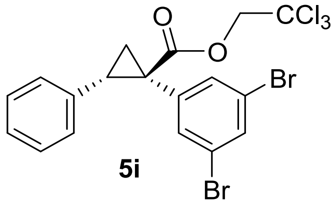

5i

2,2,2-trichloroethyl (1R,2S)-1-(3,5-dibromophenyl)-2-phenylcyclopropane-1-carboxylate (3i)

Prepared according to general procedure A for bench-scale cyclopropanation. Styrene (115 µL, 1.0 mmol, 5.0 equiv), 2,2,2-trichloroethyl 2-diazo-2-(3,5-dibromophenyl)acetate (90.3 mg, 0.200 mmol, 1.0 equiv), and Rh_2(*S*-TPPTTL)$_4$ (5.0 mg, 1 mol%, in benzoylated cellulose membrane) were used. Purification by flash column chromatography (0% hexanes/diethyl ether, 0-10% hexanes/diethyl ether) afforded **3i** as a crystalline solid (67.1 mg, 64%, 20% ee). Spectra matched the literature precedent.[1]

^{1}H NMR (400 MHz, $CDCl_3$) δ 7.43 (t, *J* = 1.7 Hz, 1H), 7.19 – 7.12 (m, 5H), 6.88 – 6.81 (m, 2H), 4.86 (d, *J* = 11.9 Hz, 1H), 4.62 (d, *J* = 11.9 Hz, 1H), 3.23 (dd, *J* = 9.4, 7.5 Hz, 1H), 2.27 (dd, *J* = 9.4, 5.4 Hz, 1H), 2.00 (dd, *J* = 7.6, 5.4 Hz, 1H).

HPLC: The enantiopurity was determined to be 20% ee by chiral HPLC analysis (Chiracel AD-H, 1.0% IPA/Hexanes, 1.0 mL/min, λ=230 nm, retention time of 5.83 min (major) and 7.46 min (minor).

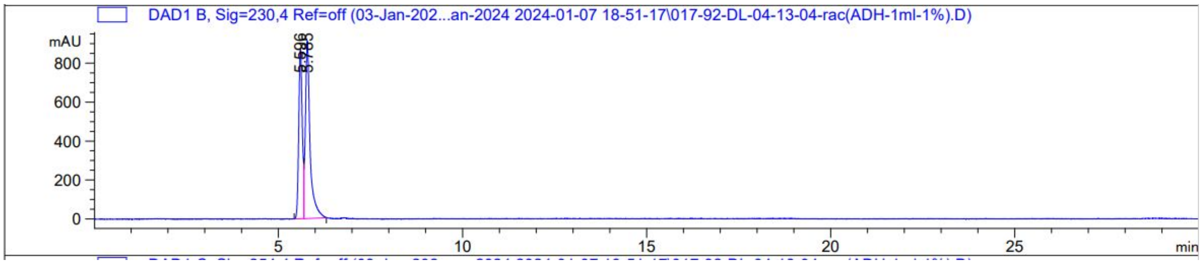

Signal 2: DAD1 B, Sig=230,4 Ref=off

Peak #	RetTime [min]	Type	Width [min]	Area [mAU*s]	Height [mAU]	Area %
1	5.596	BV	0.0932	5825.32764	869.18481	41.6935
2	5.785	VV R	0.1160	8146.47119	909.18353	58.3065

Totals : 1.39718e4 1778.36835

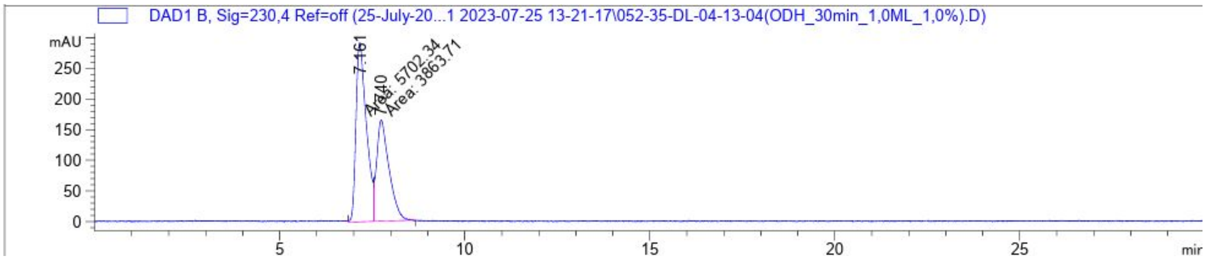

Signal 2: DAD1 B, Sig=230,4 Ref=off

Peak #	RetTime [min]	Type	Width [min]	Area [mAU*s]	Height [mAU]	Area %
1	7.161	MF	0.3245	5702.33838	292.87137	59.6102
2	7.740	FM	0.3888	3863.70581	165.61328	40.3898

Totals : 9566.04419 458.48465

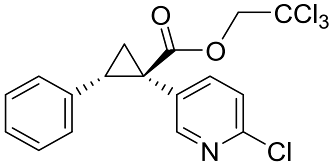

5j

2,2,2-trichloroethyl (1R,2S)-1-(6-chloropyridin-3-yl)-2-phenylcyclopropane-1-carboxylate (3j)

Prepared according to general procedure A for bench-scale cyclopropanation. Styrene (115 µL, 1.0 mmol, 5.0 equiv), 2,2,2-trichloroethyl 2-(6-chloropyridin-3-yl)-2-diazoacetate (65.8 mg, 0.200 mmol, 1.0 equiv), and Rh_2(*S*-TPPTTL)$_4$ (5.0 mg, 1 mol%, in benzoylated cellulose membrane) were used. Purification by flash column chromatography (0% hexanes/diethyl ether, 0-10% hexanes/diethyl ether) afforded **3j** as a crystalline solid (57.2 mg, 71%, **90% ee**). Spectra matched the literature precedent.[1]

^{1}H NMR (400 MHz, CDCl$_3$) δ 8.14 (dd, *J* = 2.5, 0.7 Hz, 1H), 7.30 – 7.25 (m, 1H), 7.14 (dd, *J* = 5.0, 1.9 Hz, 3H), 7.06 (dd, *J* = 8.2, 0.8 Hz, 1H), 6.86 – 6.79 (m, 2H), 4.84 (d, *J* = 11.9 Hz, 1H), 4.65 (d, *J* = 11.9 Hz, 1H), 3.27 (dd, *J* = 9.4, 7.5 Hz, 1H), 2.35 (dd, *J* = 9.4, 5.4 Hz, 1H), 2.05 (dd, *J* = 7.5, 5.4 Hz, 1H).

HPLC: The enantiopurity was determined to be 90% ee by chiral HPLC analysis (Chiracel AD-H, 2.0% IPA/Hexanes, 1.0 mL/min, λ=230 nm, retention time of 13.52 min (major) and 20.45 min (minor).

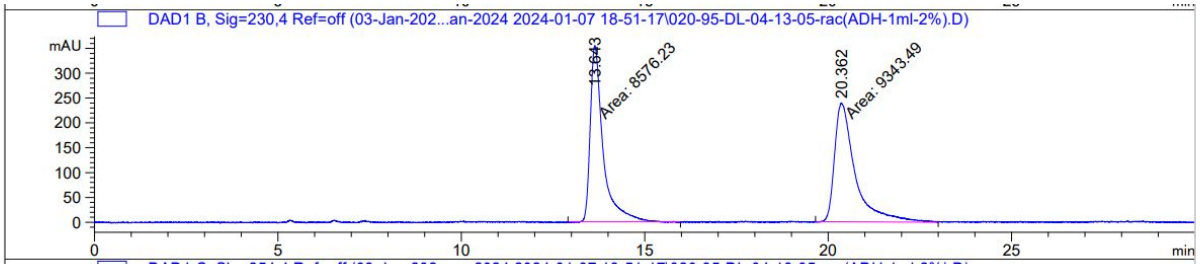

Signal 2: DAD1 B, Sig=230,4 Ref=off

Peak #	RetTime [min]	Type	Width [min]	Area [mAU*s]	Height [mAU]	Area %
1	13.643	MM	0.4031	8576.22949	354.55942	47.8592
2	20.362	MM	0.6505	9343.48730	239.40225	52.1408
Totals :				1.79197e4	593.96167	

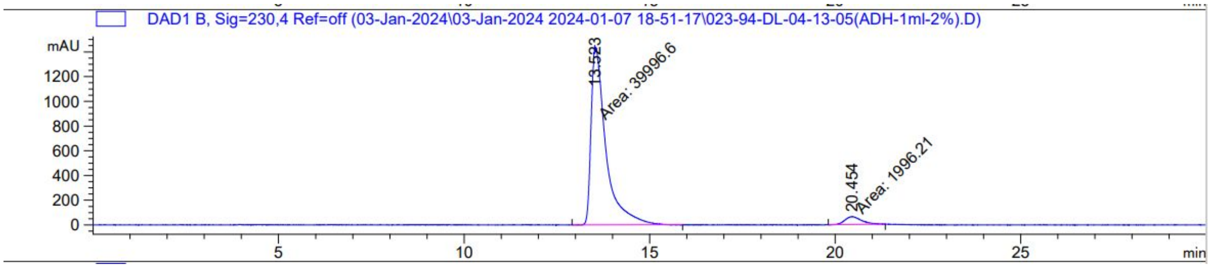

Signal 2: DAD1 B, Sig=230,4 Ref=off

Peak #	RetTime [min]	Type	Width [min]	Area [mAU*s]	Height [mAU]	Area %
1	13.523	MM	0.4596	3.99966e4	1450.40259	95.2463
2	20.454	MM	0.5184	1996.21399	64.17412	4.7537

Totals : 4.19928e4 1514.57671

9. Copies of NMR spectra

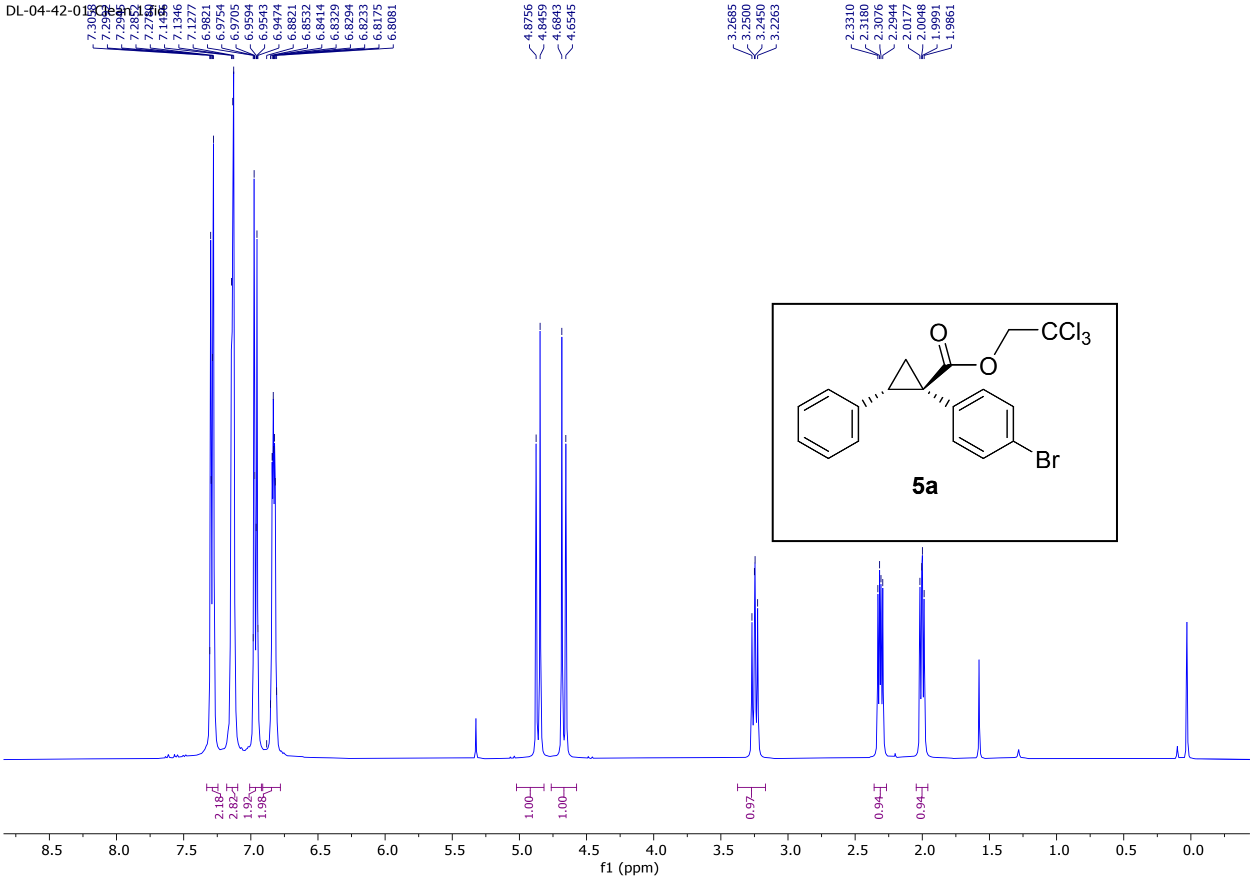

Figure S16. ^{1}H-NMR spectrum of **5a.**

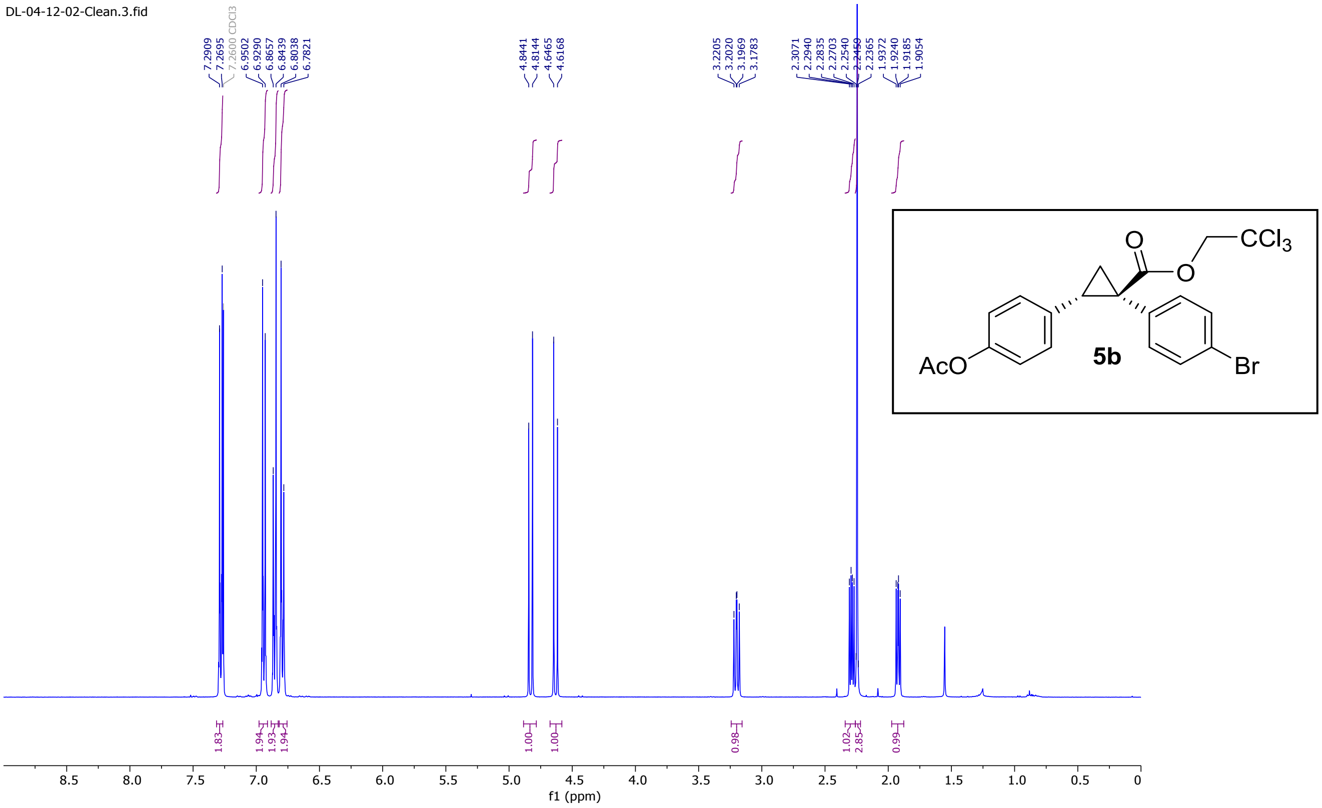

Figure S17. ^{1}H-NMR spectrum of **5b.**

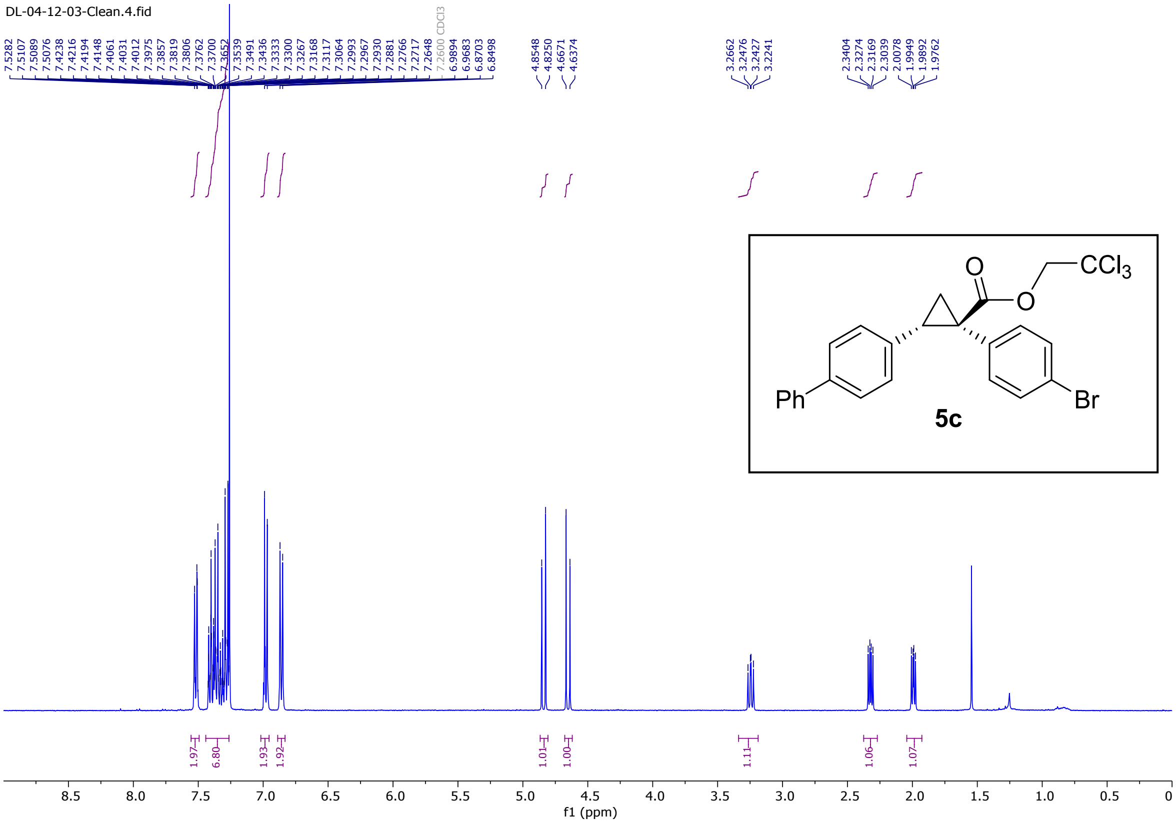

Figure S18. ^{1}H-NMR spectrum of **5c.**

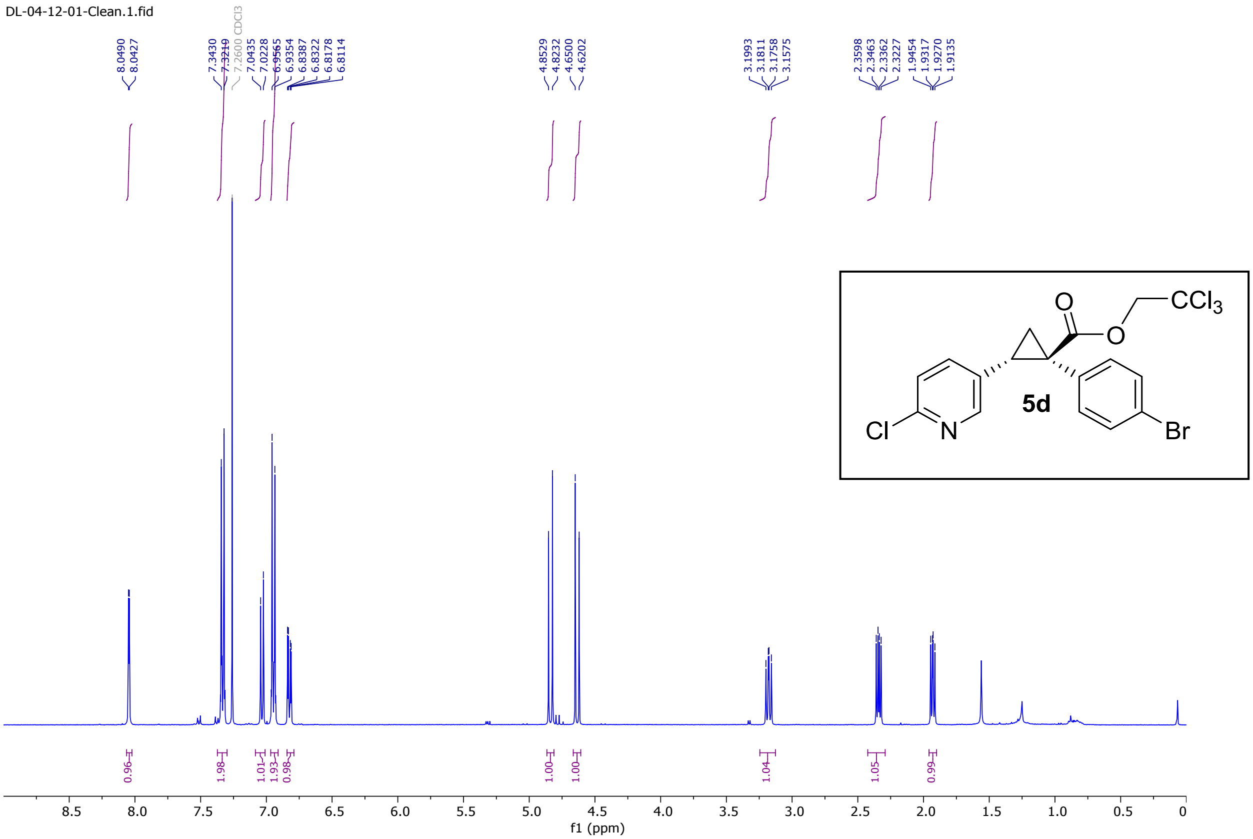

Figure S19. ^{1}H-NMR spectrum of **5d.**

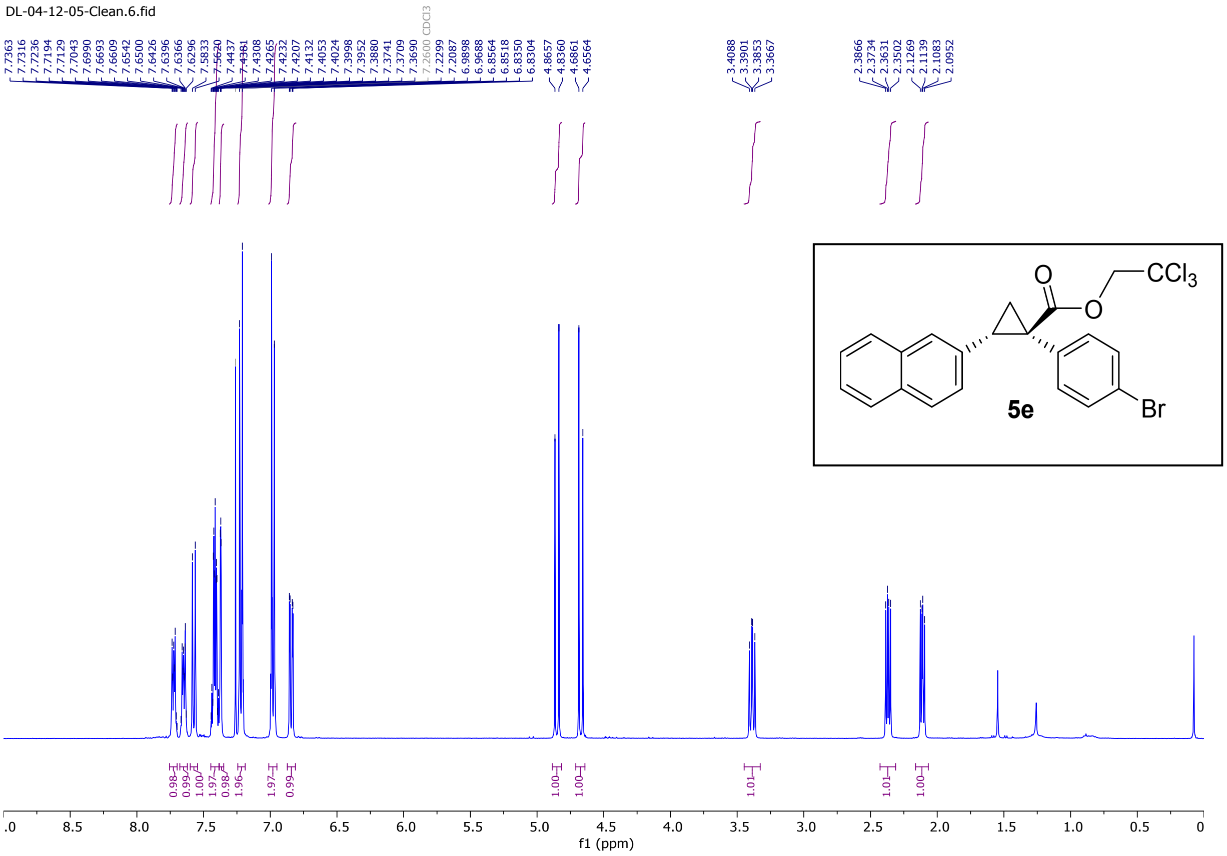

Figure S20. ^{1}H-NMR spectrum of **5e.**

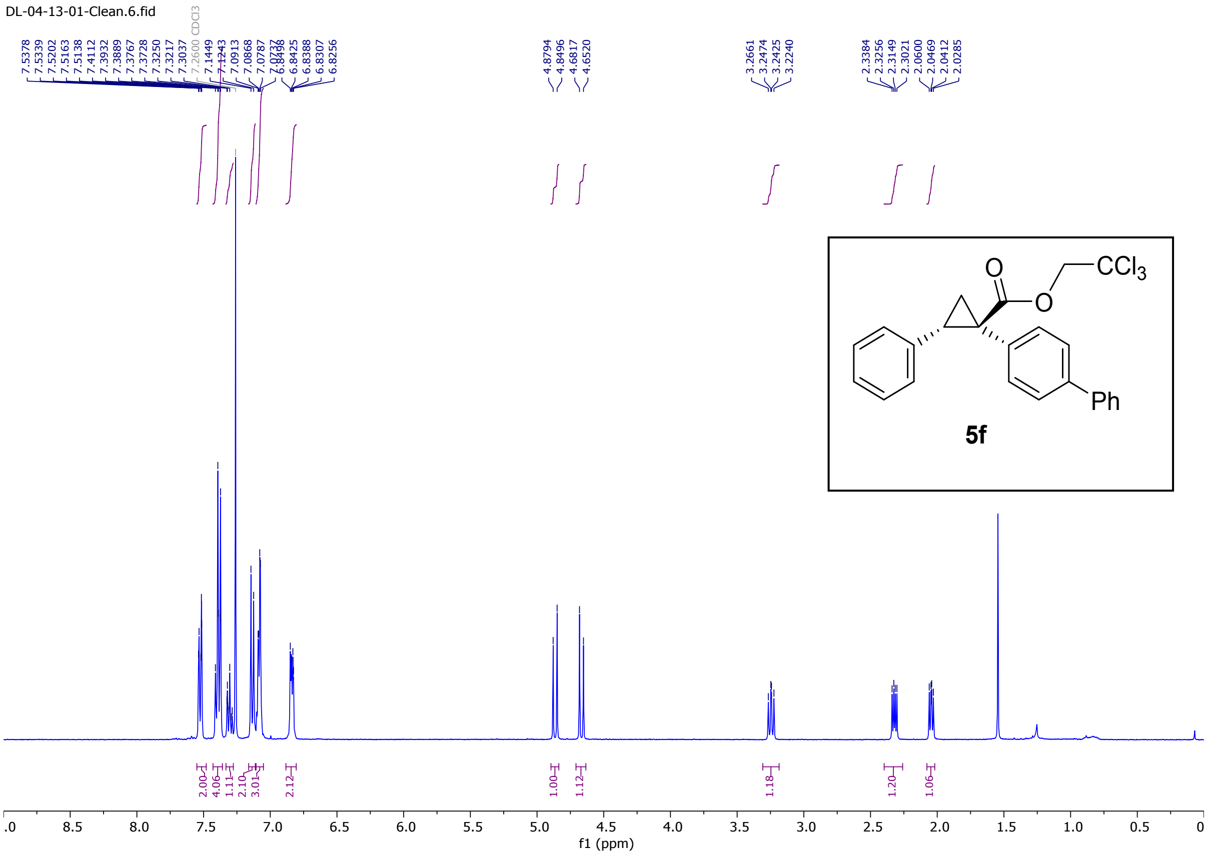

Figure S21. ^{1}H-NMR spectrum of **5f.**

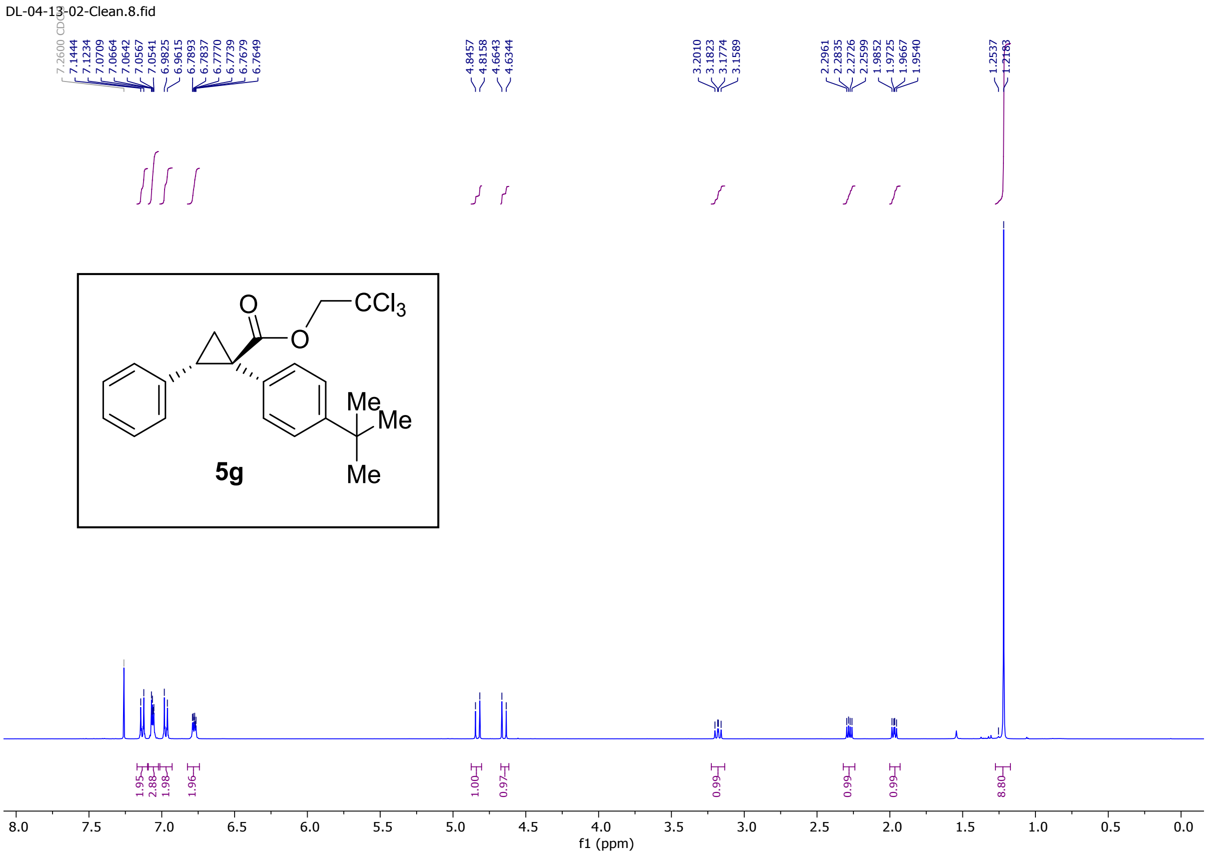

Figure S22. ^{1}H-NMR spectrum of **5g.**

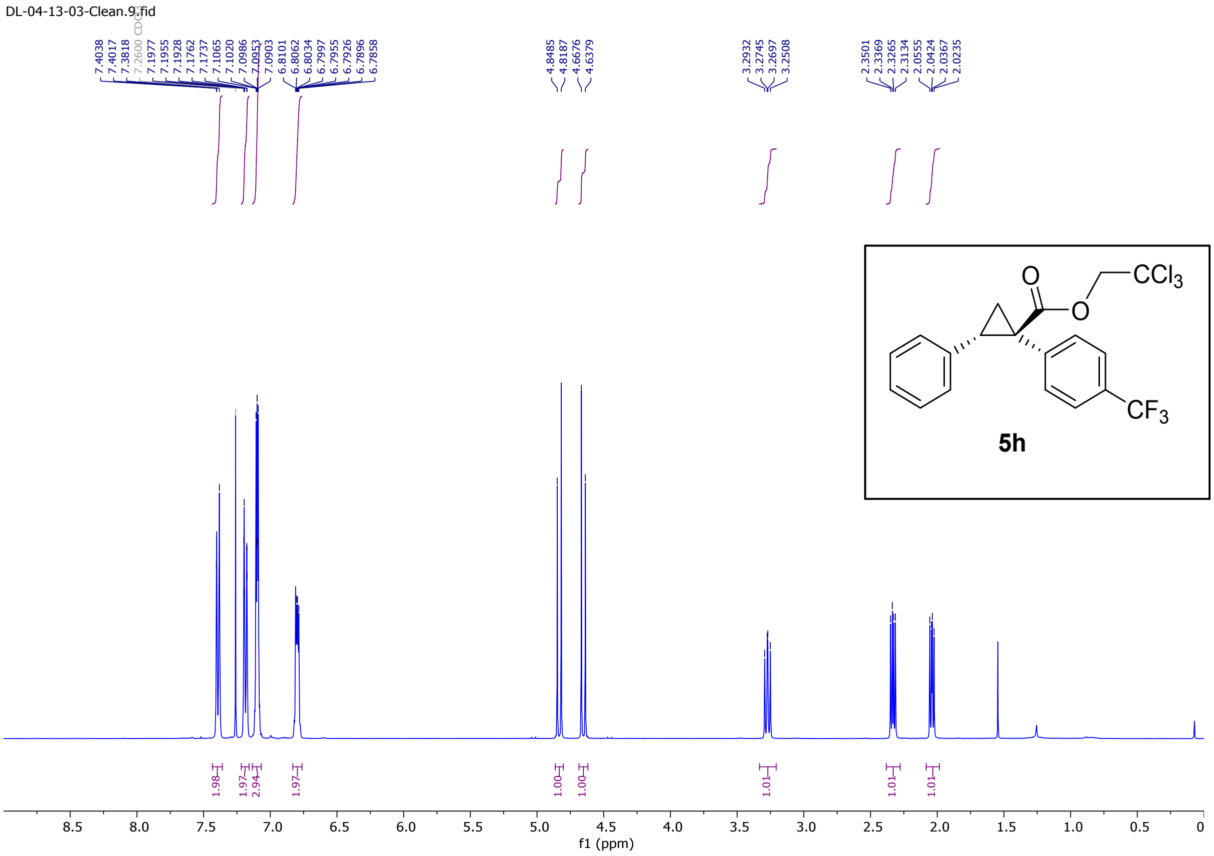

Figure S23. ^{1}H-NMR spectrum of **5h.**

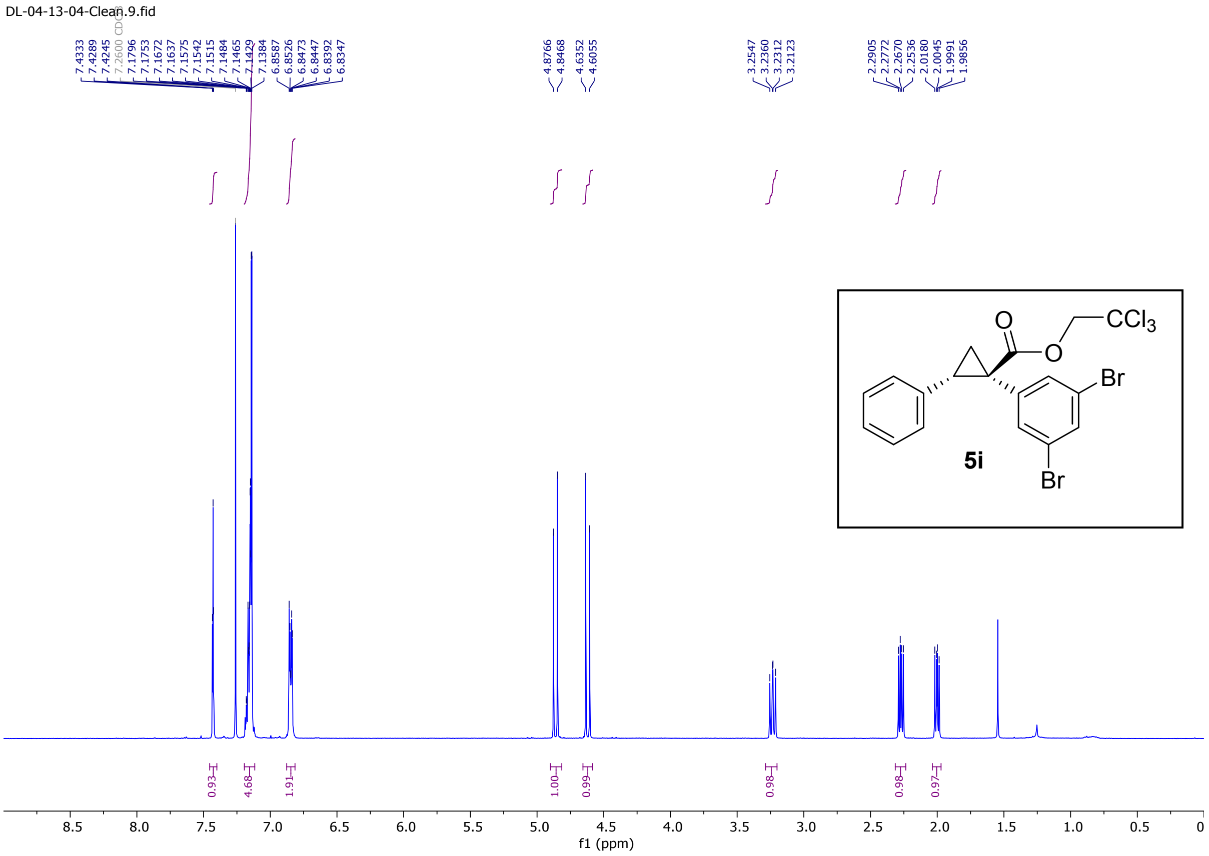

Figure S24. ^{1}H-NMR spectrum of **5i.**

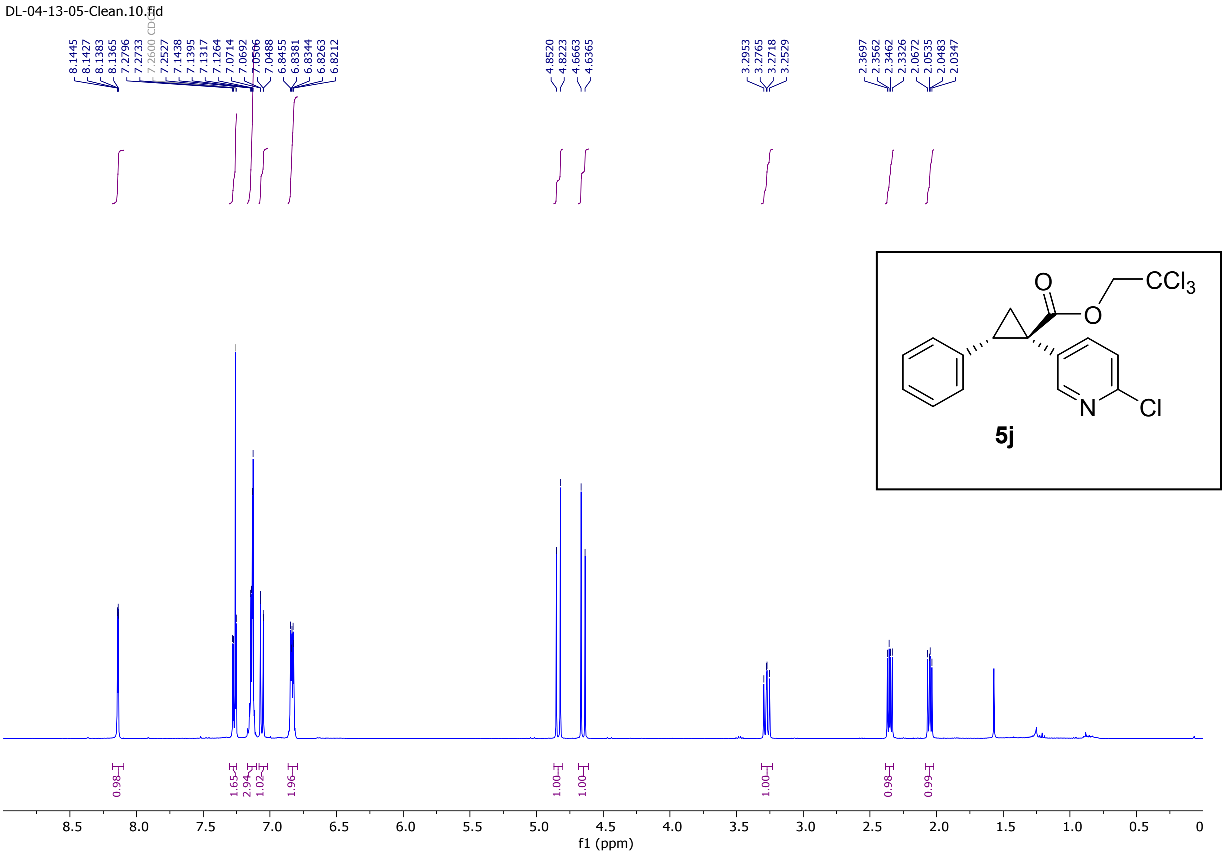

Figure S25. ^{1}H-NMR spectrum of **5j.**

References

(1) Wei, B.; Sharland, J. C.; Lin, P.; Wilkerson-Hill, S. M.; Fullilove, F. A.; McKinnon, S.; Blackmond, D. G.; Davies, H. M. L. In Situ Kinetic Studies of Rh(II)-Catalyzed Asymmetric Cyclopropanation with Low Catalyst Loadings. *ACS Catalysis* **2020**, *10* (2), 1161-1170. DOI: 10.1021/acscatal.9b04595.

(2) Guptill, D. M.; Davies, H. M. L. 2,2,2-Trichloroethyl Aryldiazoacetates as Robust Reagents for the Enantioselective C–H Functionalization of Methyl Ethers. *Journal of the American Chemical Society* **2014**, *136* (51), 17718-17721. DOI: 10.1021/ja5107404.

(3) Sharland, J. C.; Wei, B.; Hardee, D. J.; Hodges, T. R.; Gong, W.; Voight, E. A.; Davies, H. M. L. Asymmetric synthesis of pharmaceutically relevant 1-aryl-2-heteroaryl- and 1,2-diheteroarylcyclopropane-1-carboxylates. *Chemical Science* **2021**, *12* (33), 11181-11190, 10.1039/D1SC02474D. DOI: 10.1039/D1SC02474D.

(4) Sailer, J. K.; Sharland, J. C.; Bacsa, J.; Harris, C. F.; Berry, J. F.; Musaev, D. G.; Davies, H. M. L. Diruthenium Tetracarboxylate-Catalyzed Enantioselective Cyclopropanation with Aryldiazoacetates. *Organometallics* **2023**, *42* (15), 2122-2133. DOI: 10.1021/acs.organomet.3c00268.
